# Practical aspects of teaching a graduate-level small-mol­ecule chemical crystallography course

**DOI:** 10.1107/S2056989025010527

**Published:** 2026-01-01

**Authors:** John F. Berry, Ilia A. Guzei

**Affiliations:** a Department of Chemistry, University of Wisconsin-Madison, 1101 University Ave, Madison, WI 53706, USA; Harvard University, USA

**Keywords:** X-ray single-crystal diffractometry, pedagogy, structure solution and refinement, practical crystallographic exercises

## Abstract

We present the practical curriculum of the graduate Chemical Crystallography course in the Department of Chemistry of the University of Wisconsin–Madison, outlining our favorite resources, texts, and software. The course relies on collaborative in-class student assignments that build up a knowledge base of fundamental crystallography and 37 hands-on structure solution and refinement exercises spanning structures of varying difficulty. Students begin solving and refining structures early in the course, following a ‘do first, understand second’ philosophy. The strong focus on data inter­pretation ensures that students gain the practical skills necessary for independent structural investigation and critical evaluation of the crystallographic literature.

## Abstract 2

We share our pedagogical vision for a graduate-level small-mol­ecule chemical crystallography in-class exercises and list our preferred textbooks, computer programs, and websites.

## Introduction

Modern single-crystal X-ray diffraction is the most powerful and unambiguous analytical method for the absolute structure elucidation of solids. Small-mol­ecule X-ray diffractometry is a well-established analytical technique that provides a wealth of information from a very small amount of crystalline material. Automated in-house X-ray diffractometers, collaborations with synchrotron beamline scientists, and easy-to-use crystallographic software programs make the technique accessible to professional and aspiring crystallographers alike. However, despite an apparent low barrier of entry, single-crystal X-ray analysis is not always straightforward and sufficient expertise and knowledge are necessary to ensure that the results (refined crystal structures) are correct. The ability to evaluate the quality of published structures is also necessary for a chemist because of the sheer number of structural studies published in chemistry journals. For example, among the 2999 articles published in the *Journal of the American Chemical Society* in 2023 as indexed in SciFinder (American Chemical Society, 2025*a* ▸), 837 of these – 28% – reported the results of 3157 small-mol­ecule structural analyses. Just in 2023, 60045 structures were deposited in the Cambridge Structural Database (CSD; Groom *et al.*, 2016 ▸), 15474 into the Protein Data Bank (Berman *et al.*, 2000 ▸), and 21629 into the Inorganic Crystal Structure Database (Zagorac *et al.*, 2019 ▸). Yet, access to X-ray crystallographic facilities is not considered to be a critical infrastructure requirement for an American Chemical Society approved undergraduate program (American Chemical Society, 2025*b* ▸). Crystallography is taught sporadically at the undergraduate level in the USA. Nonetheless, reports are available on crystallography education (Grazulis *et al.* 2015 ▸; Zheng *et al.*, 2025 ▸; Zheng & Parkin, 2025 ▸), promotion and understanding of structural science (Bou-Nader *et al.*, 2025 ▸), courses in macromolecular (Stiers *et al.*, 2016 ▸; Grundwell *et al.*, 1999 ▸) and small-mol­ecule (Aldeborgh *et al.*, 2014 ▸) crystallography and crystal growth (Whelan *et al.*, 2018 ▸; Arkhipov *et al.*, 2022 ▸; Staples, 2025 ▸), case-based learning modules (Dong & Zheng, 2021 ▸), and the significance of databases (Bruno *et al.*, 2017 ▸). Chemical crystallography is more often offered as a graduate level course. At the UW–Madison Department of Chemistry, this biennial 600-level course is designed for individuals who desire to acquire basic crystallographic knowledge, mathematical foundations of diffraction principles, and hands-on experience in the operation of diffractometers, computer software, and crystal structure determination. The course provides the concepts of crystallographic analysis including point/space group symmetry, the use of the reciprocal lattice to understand diffraction by crystals, and crystallographic experiment design. The course is typically taken by 12–20 graduate students in chemistry and related fields, as well as a few intrepid undergraduate students.

Herein we share details of our selection of textbooks used for the class and describe the teaching philosophy of the course, the in-class discussion activities, and the practical examples, which encompass most of the problems a crystallographer encounters during structural refinements. This is a one-semester, three-credit course; it starts with three 50-minute lectures per week and when practical sessions begin, one 50-minute lecture period is replaced with a two-hour hands-on session per week. Students acquire data on their crystals outside of the class hours.

## Literature sources

There are a few popular textbooks used in graduate level crystallography courses, but none covers all aspects of our course materials according to our vision. Thus, a collection of titles is typically necessary to organize the material. We very briefly comment on the books that we have been using for teaching during the last decade. The list is not exhaustive, and our goal is not to provide a comprehensive review of crystallography textbooks; rather, if you were a friend who wanted to create a ‘Chemical crystallography’ course and asked for our recommendations, this is what we would share.

The recommended textbook for the course is Gregory Girolami’s ‘X-ray crystallography’ (Girolami, 2015 ▸), written by a chemist for chemists practicing crystallography. The text covers the practical aspects of single-crystal structural analysis well without being mathematically demanding. The chapters are short, the presentation is clear, and the end-of-chapter exercises are few but well-chosen. If you were to buy just one book to learn crystallography, this would be it.

A popular introductory text is Werner Massa’s ‘Crystal Structure Determination’ (Massa, 2004 ▸), which we recommend to students who want to learn crystallography basics, but not necessarily take an X-ray course.

Walter Borchardt-Ott’s ‘Crystallography’ (Borchardt-Ott, 1993 ▸) offers the best introduction to stereographic projections (when was the last time you used the Wulff Net?), symmetry elements, point groups with mol­ecular symmetry examples, space groups, and *hkl* indices. The derivation of the reciprocal space and Ewald sphere construction are particularly good. Most of the 13 chapters have many exercises with an answer key in the back of the book.

The classic ‘Crystal Structure Analysis for Chemists and Biologists’ by Jenny P. Glusker *et al.* (1994 ▸) is a go-to source for topics such as diffraction by crystals, wave combinations, visual representation of Fourier transformations and atomic vibration effects, and a source of useful formulae that may not be found elsewhere. The sections on mol­ecular structural features teach the reader how to discuss crystal structures.

Another good source on the theory of structure factors and analysis of measured intensities is ‘X-ray Structure Determination: a Practical Guide’ by Stout & Jensen (1989 ▸).

Maureen Julian’s ‘Foundations of Crystallography with Computer Applications’ (Julian, 2008 ▸) has an excellent introduction to point groups, vectors, matrix transformations, multiplication tables, and basic crystallographic computing. The book follows the IUCr convention in the unit-cell discussions. Students find the illustrated material easy to absorb, which makes the introduction of space groups later in the course easier.

Many crystallographers and chemists used Ladd and Palmer’s ponderous ‘Structure Determination by X-ray Crystallography’ (Ladd & Palmer, 1993 ▸) during the 90s. We primarily use this book as a source of problem sets.

Students who are excited about growing crystals are addressed to the informative ‘Crystals and Crystal Growing’ by Holden & Morrison (1982 ▸), which is also recommended to the participants of the Wisconsin statewide crystal-growing competition (Guzei, 2014*b* ▸), whereas those fascinated by crystal inter­action with light are recommended the delightful ‘Crystals and Light (an introduction to optical crystallography)’ by Wood (1977 ▸). Experimentalists who desire to learn more about their crystals from the diffraction patterns are referred to ‘Atlas of Optical Transforms’ by Harburn *et al.* (1975 ▸).

A very useful and concise text on refinement and the use of the *SHELXL* software (Sheldrick, 2015*b* ▸) is ‘Crystal Structure Refinement’ by Mueller *et al.* (2006 ▸).

We also use online crystallographic resources at Otterbein University (Johnston, 2022 ▸) and the symmetry and space group tutorial hosted by Brandeis University (Jasinski & Foxman, 2015 ▸).

There are two time-tested texts ‘Fundamentals of Crystallography’ (Giacovazzo *et al.*, 1992 ▸) and Jack Dunitz’s ‘X-ray Analysis and the Structure of Organic Molecules’ (1995 ▸). The former is comprehensive and very mathematical; the latter is a classic crystallography text with a large portion allocated to the application of the technique to organic chemistry.

Of course, our curriculum would be incomplete without the Inter­national Tables for Crystallography volume A (Inter­national Tables, 2002 ▸) that finds extensive application in the practical portion of the course.

The course materials and lectures are constructed to optimally meet our objectives by extracting relevant sections from the aforementioned books.

## Teaching philosophy

### ‘Do first, understand second’

For better or for worse, one does not need to know much about crystallography to solve a routine structure using modern software. We capitalize on this fact and introduce students to structure solution very early in the course. This accomplishes several important things. First, it allows us to work with students who have software installation issues and to get these issues addressed as soon as possible. Thus, we maximize the time during the course in which students are able to use the crystallographic software, described in more detail in section 5.2, as a tool. Second, students are introduced to the vocabulary of crystallography as early as possible, and are shown how to use the vocabulary in context. Students may not yet know what a space group is, or reflection conditions, but they learn quickly that the space group must be determined using reflection conditions before a structure can be solved. Third, solving their first crystal structure provides students with an undeniable sense of wonder and accomplishment, both of which serve to further motivate them to better understand the science of how crystallography works.

To prepare students for the ‘do first, understand second’ philosophy, in the first class meeting, groups are given a set of note cards with step-by-step instructions for how to complete a crystallographic experiment. Steps include ‘Mount the Crystal on the Diffractometer’, ‘Use *checkCIF*/*PLATON* to Validate the Structure’, ‘Refine the Structure’, ‘Assign the Lattice Type’, among many others (see the supporting information). The cards are out of order, and the groups are tasked with proposing a correct order for the steps. Without any in-depth knowledge of the process, and some help from instructors who provide brief descriptions of any unknown terms (*e.g.*, ‘CIF’ or ‘Absorption Correction’), groups propose a mostly correct logical sequence. The sequences of the class groups are presented, compared, and discussed. As a reading assignment, we provide the students with C. F. Campana’s excellent and concise summary of the crystallographic experimental process (Campana, 2014 ▸).

### Collaborative discussion

The most impactful applications of crystallography are close collaborations with other fields. Thus, one of the goals for the class is to teach students how to collaborate and how to have productive scientific discussions. In the classroom, students are seated around tables in groups of four-to-six to facilitate discussion. This course highly leverages ideas and concepts from ‘The Discussion Project’ (Cramer, 2025 ▸) a teaching workshop taken by one of us that is offered on the UW–Madison campus. The core concepts are derived from literature studies that find that discussion and peer instruction provide superior learning outcomes in students as compared to traditional lectures (Agwu & Nmadu, 2023 ▸; Deslauriers *et al.*, 2019 ▸; Freeman *et al.*, 2014 ▸). During the 50-minute periods, new student knowledge is generated by the students themselves *via* a series of exercises that the students work together to solve. These are contextualized puzzles, beginning with abstract illustrations of symmetry principles and culminating with analysis of real data sets. Similar types of exercises have been developed in our department to teach spectroscopic methods (Esselman *et al.*, 2025 ▸). Students are given time in class to work on these exercises together with their groups while course instructors circulate to provide guidance and encouragement. The exercises are designed to be difficult but surmountable. Often, they illustrate a concept that has not yet been discussed in class (*e.g*., that a twofold axis perpendicular to a mirror plane generates an inversion center). Once all groups have worked through the exercises, a whole-class discussion of their results ensues.

## Crystallography exercises

### Symmetry exercises

The course begins with symmetry concepts, and we have created a series of exercises that are designed to allow students to discover increasingly complex symmetry concepts, Table 1 ▸.

Mastery of symmetry requires strong visuospatial skills. In order to facilitate development of these skills, we created a set of publicly available 3D models that are utilized in several of the symmetry exercises described in Table 1 ▸ (Aristov *et al.*, 2022 ▸). Other exercises involve identification of symmetry elements in artwork such as ribbons or wallpaper. For space groups, we use diagrams from Volume A of the Inter­national Tables, and 3D models inspired by Meier’s ‘14 Space Group Problems’ pamphlet (Meier, 2001 ▸). By using 3D models, we avoid the ambiguity in these exercises pointed out by Suescun & Nespolo (2012 ▸) derived from the inter­pretation of footprints as being flat objects with inherent mirror symmetry. These exercises lead to productive discussions among the students as they are challenged to convince their peers or their instructors of the symmetry elements that they identify.

### Diffraction exercises

We have additionally developed a second set of exercises to guide students through crystallographic topics beyond symmetry, Table 2 ▸.

**Figure 1:**
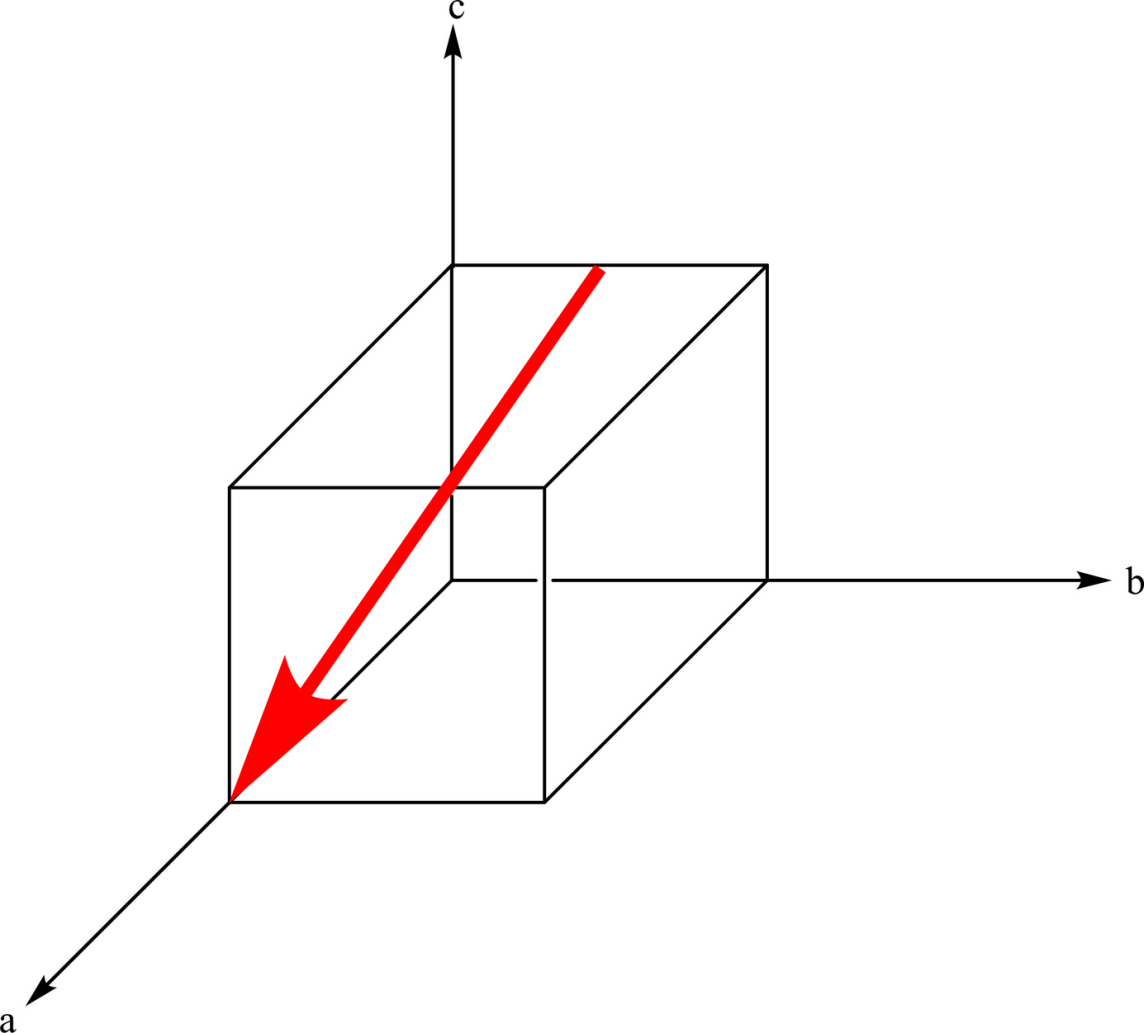
Example problem from the directions and planes exercise. The students are given this figure and asked to determine the direction of the red arrow [2
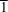

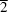
].

The directions and planes exercise provides students with direction indices [*uvw*] or plane indices (*hkl*), and asks them to draw the associated direction or plane within a generic unit cell. Additional problems have directions or planes already drawn (*e.g*., a direction is shown in Fig. 1 ▸), and the students are asked to determine indices for these. These exercises range in difficulty from simple indices with values of ±1 or 0 to more complicated directions with higher indices. During this exercise, the students are able to discover that directions and planes having the same set of indices are normal to each other.

The diffraction exercises include the introduction of the ‘Inter­active Structure Factor Tutorial’ (Cowtan, 2025 ▸). It illustrates the relationship between the direct and reciprocal spaces and how the *hkl* indices, amplitudes, and phases of reflections are defined with the Argand diagram and how they specify electron density waves and affect electron density maps.

For the powder diffraction exercise, the students are either given a list of diffraction angles, 2θ, or are provided with a diffractogram from which they must determine the 2θ values. Importantly, real data sets for cubic structures are used, typically pulled from the Inter­national Center for Diffraction Data. Students are asked to determine the lattice constant and work together to compare their results along the way.

The structure factor exercises mostly consist of problems from Ladd & Palmer, and require students to calculate structure factors for simple structures mathematically. Notably, in Exercise #3, students derive Friedel’s law without having been previously introduced to it.

**Figure 2:**
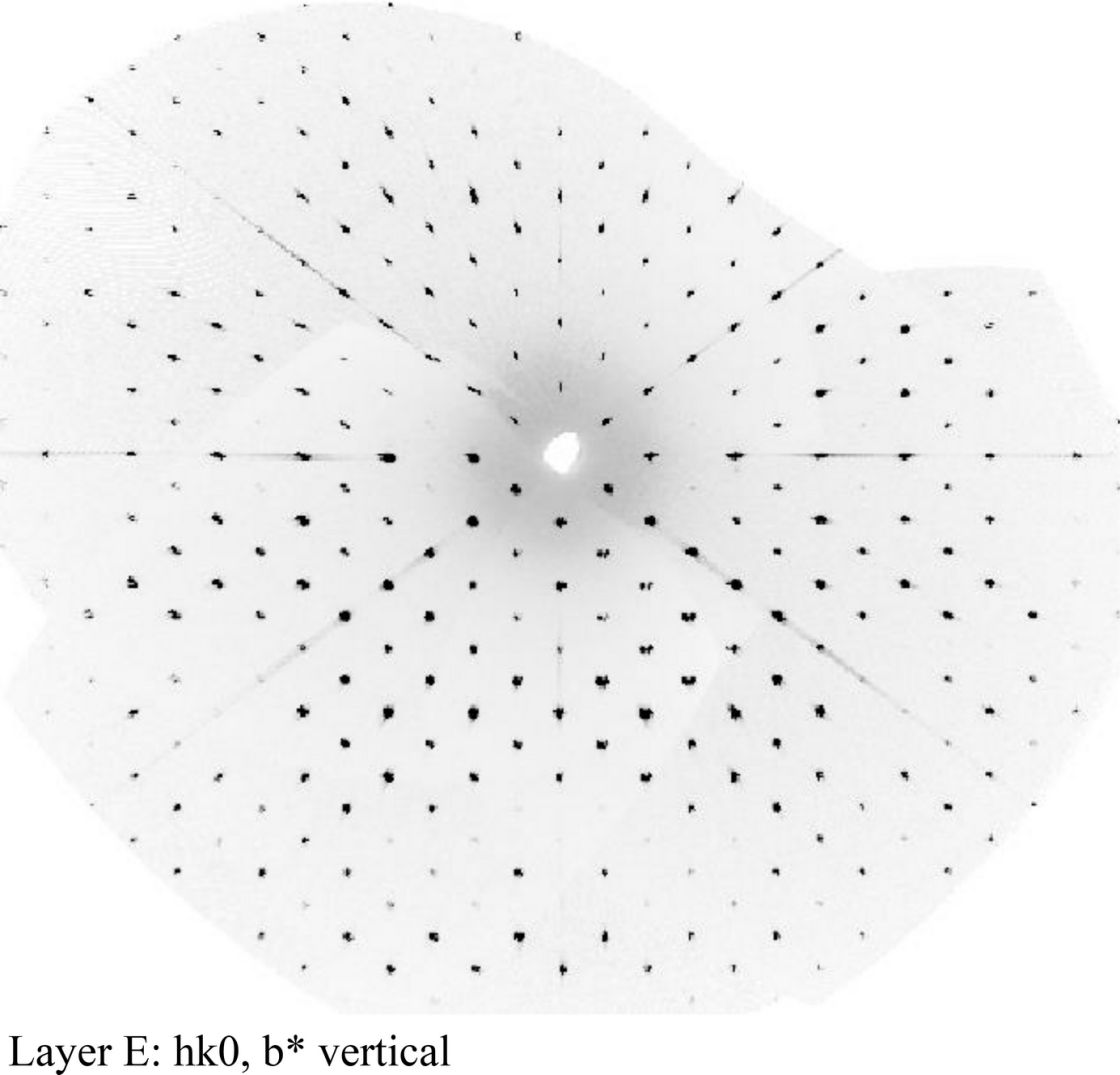
Example of a crystallographic zone image from the data-analysis exercise.

The data analysis exercise uses real data sets collected in our lab. Students are provided with a series of zone photos that are reconstructed from diffraction data such as the one shown in Fig. 2 ▸. By examining the diffraction pattern in the *0kl, 1kl, h0l, h1l, hk0, and hk1* layers, students are able to determine the relationship between the reciprocal constants and reflection conditions, and thereby the likely space group candidates.

The direct methods exercise tasks students with phasing reflections from a real dataset given *hkl* indices and *E* values. We follow closely the procedure as outlined in Girolami’s text.

### Mathematical Preparation

Students taking the chemical crystallography course have ranged in experience from freshman undergraduates to second or third–year graduate students. The only course requisite is graduate standing, which is waived for undergraduates so long as they show sufficient motivation and enthusiasm for the course. We do not specify math prerequisites, but the course obviously deals with mathematical topics. To bring students up to speed, or to provide a welcome review, we supply students with ‘cheat sheets’ on the two most important advanced math topics: vectors & matrices, and complex numbers. We briefly review the contents of these ‘cheat sheets’ in class, but do not work problems that deal with these concepts outside of the context of a crystallographic problem. Copies of these ‘cheat sheets’ are included in the supporting information.

### General observations on peer learning

The above exercises generally lend themselves well to classroom discussions because complete solutions require extracting many pieces of information from the available data sets. Instructors focus students’ attention on what they observe, whether their peers agree with their observations, and the greater meaning behind what has been observed. Students enter the class with a broad range of visuospatial skills: some students see symmetry elements right away while other require more time. We ask groups to work together to develop a consensus on their observations among all the students in the group. Our observations are that these peer learning exercises have been successful, however, the class size is insufficiently large for a more rigorous, qu­anti­tative, and statistical study.

## Practical sessions

### Data acquisition

The practical sessions include experimental and computational parts. The former requires the students to obtain a crystal of their choice and characterize it by single-crystal X-ray diffractometry. The class is introduced to crystal growing, optical microscopy with polarized light, crystal evaluation and selection techniques, and experiment planning and design. After an appropriate X-ray radiation wavelength is chosen, a diffraction experiment either on a Cu *K*α or on a Mo *K*α radiation Bruker Photon III diffractometer equipped with an Oxford low-temperature device follows. At this junction two free reciprocal space visualization programs, *EwaldSphere* (Barbour, 2018*a* ▸) and *XRayView* (Phillips, 1995 ▸), become extremely handy for demonstrating the application of the Ewald construction. Students learn about data-acquisition methods as well as about absorption correction and data-reduction strategies. A student may become an independent diffractometer user after taking a radiation safety course and passing formal instrument training. Those who have enough crystalline material are encouraged to also obtain a powder diffraction pattern and compare it to the simulated powder pattern computed for the single-crystal structure.

### Structure solution and refinement

Computational practical sessions require the students to solve, refine, validate, and prepare a brief report on a number of structures with a progressive degree of difficulty (Guzei, 2025*c* ▸). We use the free versions of *SHELXL* (Sheldrick, 2008*b* ▸), *OLEX2* (Dolomanov *et al.*, 2009 ▸), and *PLATON* (Spek, 2009 ▸) suites of programs as well as the invaluable reciprocal space exploration program *XPREP* (Sheldrick, 2013 ▸). The latter is proprietary, but a free analogue, the *Zürich Space Group Helper* (Solar *et al.*, 2023 ▸) is available for download and is seamlessly integrated with *OLEX2*. All one needs for space-group determination are the unit-cell parameters and HKL file, thus the space-group determination process can be agnostic relative to the hardware used for data acquisition. Whereas *XPREP* was developed for Bruker AXS Inc., two other major single-crystal X-ray diffractometer manufacturers offer tools for space-group determination: GRAL module in *CrysAlis PRO* by Rigaku Corporation (2025 ▸) and *X-RED32* by Stoe & Cie (2025 ▸). Programs *PLATON* and *Jana2020* (Petříček *et al.*, 2024 ▸) can be used with data acquired on a variety of diffractometers, and of course one can identify space group choices by a visual inspection of several precession photographs. The dual-space recycling structure-solution program *SHELXT* (Sheldrick, 2015*a* ▸) is ‘not allowed’ for structural solutions for didactic reasons – only the direct and Patterson methods as implemented in *SHELXS* are ‘allowed’, with an occasional use of *OLEX2.solve* that uses a charge-flipping algorithm but does not perform atom assignments. *SHELXT* is an exceptionally powerful program that conducts tests for space-group symmetry, solves the structure, assigns atomic identities, and refines the model; yet the goal of the class is not to show the shortest path to the result, but to teach students how to arrive at a robust and correct structural model and to evaluate determined structures independently and critically. A popular living practical user guide for *OLEX2* (Guzei, 2025*d* ▸) is freely available online. Both PC and Mac personal laptops are used in the class, with the PC users reporting a smoother experience.

**Figure 3:**
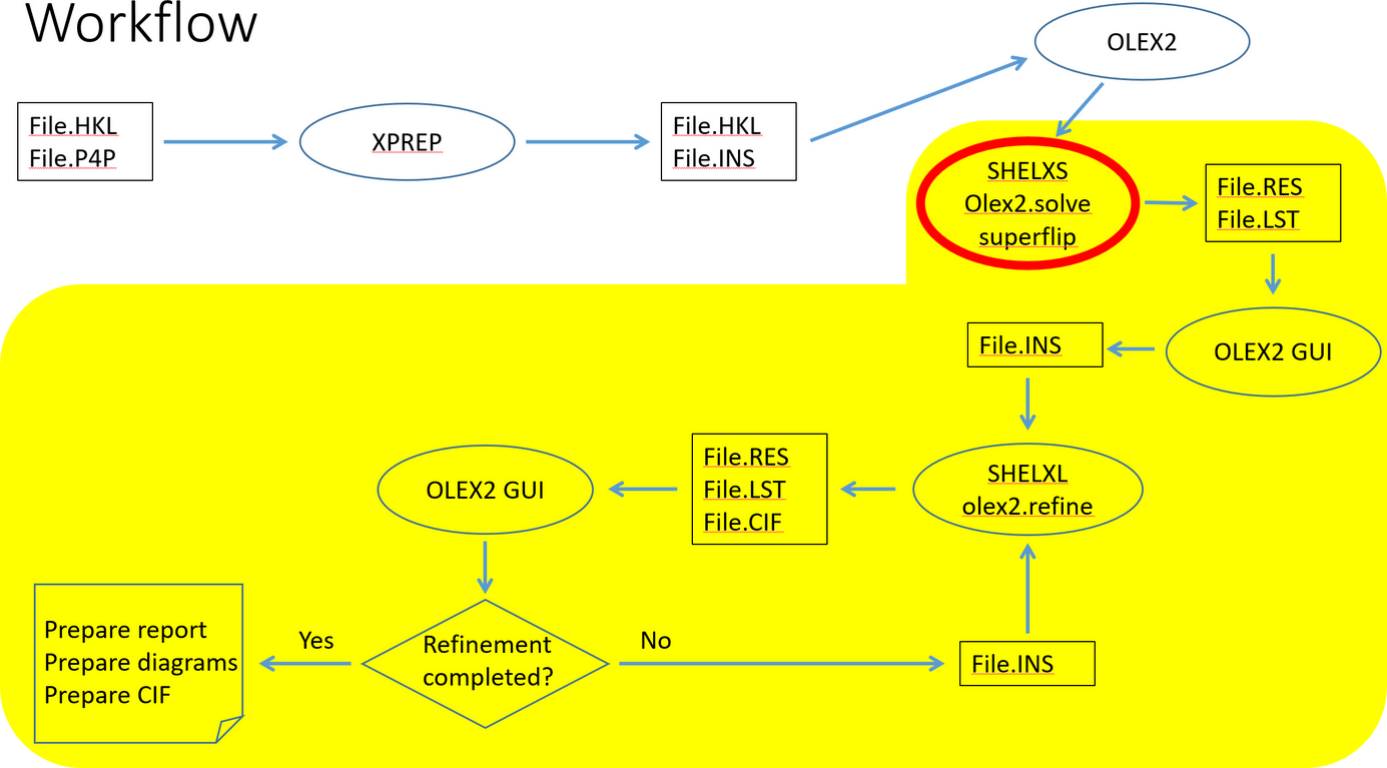
Structure solution and refinement workflow. The yellow portion corresponds to the part executed within *OLEX2*. File·INS indicates a new or revised structural model in the INStruction file. The P4P file contains the unit cell parameters, HKL – diffraction data, INS – *SHELXS*/*SHELXL* instructions for structure solution and then refinement, LST – a detailed structure solution/refinement program output, CIF – the structural data in the IUCr-specified format.

About a month into the course students begin practical sessions, even before learning about systematic absences and physics of diffraction. This follows the adage ‘Do first, understand second’. We use a hands-on approach to instruction. First, the instructor demonstrates a procedure while students observe, then the instructor repeats the demonstration as students follow along on their own laptops. All structural solutions follow the same workflow, Fig. 3 ▸, thus students gain a deeper understanding of the steps as the course progresses. The exercises are based on actual datasets collected in our department or donated by colleagues in order to prepare students for ‘real life’ structural investigations.

**Figure 4:**
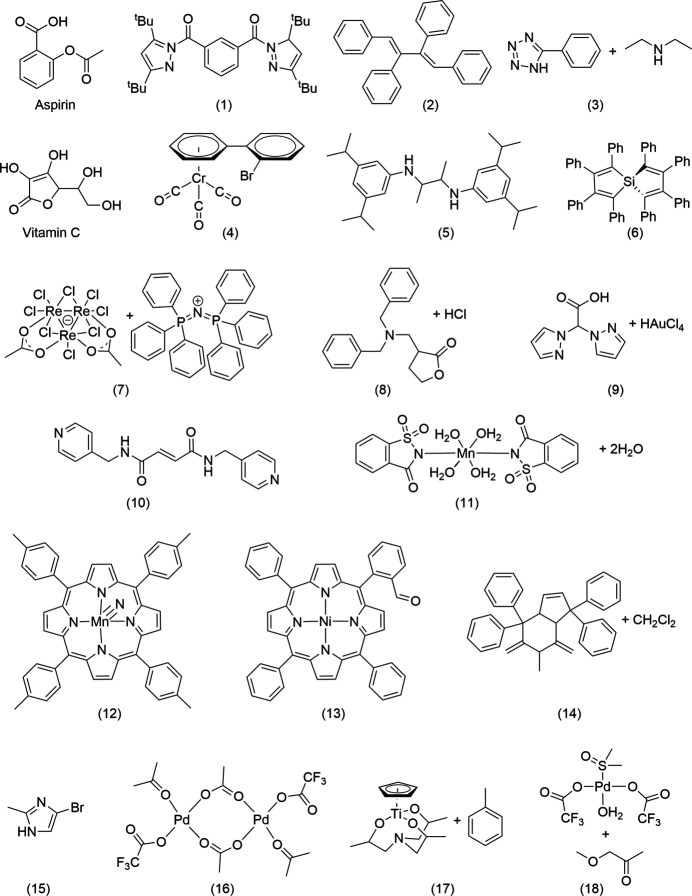
Chemical drawings of the proposed structures for the Chemical Crystallography course (see text). Many of these are ‘incorrect’ – either because the actual structure differs, or because the chemist made an incorrect drawing, or because a wrong vial was submitted.

The structures are free to download and use and if you would like to add to this collection, please contact IAG. For each exercise there is a chemical diagram of the *proposed* structure (see Figs. 4 ▸ and 5 ▸), diffraction data and unit-cell information files, and the answer key folder containing files with a complete structural model, explanation of the structure’s ‘lesson’, and relevant publication when available. The students are asked not to consult the answer key until after they have refined the structure in order to maximally benefit from the activities. The answer keys are particularly important to those who want to work on the structures independently outside of the course.

Each structure solution and refinement exercise are followed by a group discussion of the structure’s crystallographic lesson, structural features, and its chemical significance in order to foster students’ independent critical thinking. However, we usually do not finish all 37 exercises during the course due to the time limitations, but students are offered extra credit for finishing any structure not processed in the class. In the first practical session, students take a guided tour of the *OLEX2* feature-rich GUI and learn useful commands and shortcuts.

For each structure, the students are asked to:

**·** Characterize the mol­ecular symmetry of the proposed compound using the Schönflies and Hermann–Mauguin symbols.

**·** Estimate the mol­ecular volume. In the class we use the average of ‘atom count × 10’ and ‘non-H atom count × 18’ rules. [Guzei *et al.* reviewed several mol­ecular volume estimation techniques (Dolinar *et al.*, 2018 ▸) and wrote a freely available program *G1* (Guzei, 2022 ▸) that estimates mol­ecular volumes and linear absorption coefficients. The students use it later in the X-ray laboratory when they prepare to run their data acquisition experiments.]

**·** Run *XPREP* to assign the space group and prepare files for structural solution. During the program execution we discuss how many mol­ecules are present in the unit cell, the crystallographic symmetry and its relation to the mol­ecular symmetry, systematic absences, whether the mol­ecule of inter­est may reside on a special position, and data quality.

**·** Solve the structure employing direct methods (*SHELXS*).

**·** Build a chemically reasonable and computationally stable model.

**·** Finish and validate the structural refinement (Spek, 2018 ▸, 2020 ▸).

**·** Discuss the most inter­esting structural features of the final model.

**·** Complete a brief structural report including a publication-quality figure of the structure. We prefer images generated with *OLEX2*, however proprietary *XP* (Sheldrick, 1997 ▸), *Diamond* (Putz & Brandenburg, 2025 ▸), *CrystalMaker* (CrystalMaker, 2025 ▸), free or licensed Mercury (Macrae *et al.*, 2020 ▸), and free *ORTEP* (Farrugia, 2012 ▸) are viable alternatives.

The culmination of this work and ultimate test of students’ crystallographic prowess are students’ ten-minute presentations during the last week of the course, when everyone reports the results of their independent single-crystal investigations (see section 5.1) in the style of an *Acta Crystallographica E* paper. Most students are comfortable presenting their findings and speaking in front of the peer audience.

Our collection of 37 structures, Figs. 4 ▸ and 5 ▸, offers lessons and challenges with routine refinements for organic, inorganic, and organometallic compounds. This includes positional and compositional disorder, unexpected composition, mol­ecules residing on special positions, restraints, constraints, idealized fragment geometries, good and poor-quality datasets, and different types of twinning. In each discussion below, we comment on non-routine aspects of the structural studies, or ‘lessons’ each structure offers. Herein we do not discuss *all* features of each structure because they will be obvious to an experienced crystallographer and because full refinement details are available in the answer key on the web site. However, we do not want to rob those who will decide to work through the examples of the joy of finding the correct answer. In the class we introduce the structures mostly in the order shown below.

**Aspirin.** This straightforward structure has few atoms and offers a chance to discuss hydrogen-bonding inter­actions. We examine how incorrect atom assignment visually manifests itself and what strategies can be used to confirm atomic identity.

**Structure 1.** This structure features an uncomplicated structure solution. The proposed structure is *meta*-substituted whereas the actual mol­ecule is *ortho*-substituted (Guzei *et al.*, 2003 ▸). Lessons: (*a*) pay attention and trust your data; (*b*) a structure can be solved even if the proposed mol­ecular drawing is incorrect.

**Structure 2.** The mol­ecule resides on an inversion center. This becomes evident while running *XPREP*, as there are only two mol­ecules in the unit cell, but the space group is *P*2_1_/*c*. Lesson: important structural information may be available before a structure is solved. The structure matches the one reported by Karle & Dragonette (1965 ▸).

**Structure 3.** Due to the high data quality the students are asked to locate the H atoms from the difference-Fourier map. The proposed structure contains two neutral mol­ecules whereas the actual structure is ionic, resulting from a proton transfer from 5-phenyl­tetra­zole to di­ethyl­amine (Guzei & Bikzhanova, 2002 ▸). Lessons: (*a*) proton transfers are truly observed; (*b*) achiral structures can crystallize in non-centrosymmeric non-enanti­omorphous space groups (*Pna*2_1_ in this case); (*c*) it is important to learn to correctly inter­pret the Flack *x*, Hooft *y*, and Parsons’ *z* parameters given their standard uncertainties (Flack & Bernardinelli, 2000 ▸).

**Vitamin C.** The space-group assignment requires the students to take the chiral nature of the mol­ecule into account because statistical indicators favor *P*2_1_/*m* over the correct *P*2_1_ and *XPREP* does not correctly suggest the space group. We discuss enanti­omorphous, non-enanti­omorphous, and Sohncke groups and why space group *P*2_1_ is not chiral. During the structure refinement, the automatic hydrogen-atom placement routine typically fails with a few hy­droxy hydrogen atoms being oriented in wrong directions, and the students must choose the correct proton positions based on the hydrogen-bonding pattern. Atom repositioning is performed with the *OLEX2* FIT tools, which is a gateway to future disorder modeling.

**Structure 4.** Direct methods fail to solve the structure in the space group *P*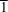 and we demonstrate a common structure solution technique that requires lowering the symmetry to *P*1, solving and partially refining the structure, investigating whether the structure should be centrosymmetric, converting the space group to *P*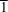 and completing the refinement (Czerwinski *et al.*, 2003 ▸). The students are asked to determine the Cr formal oxidation state and examine the inter­atomic distances in the phenyl rings to confirm that the η^6^-coordination to the Cr^0^ center results in C—C bond elongation. More adventurous students quickly discover that the charge-flipping *OLEX2.solve* correctly solves the structure in *P*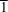.

**Structure 5.** The mol­ecule contains two chiral centers and can exist as *SS*, *RR*, or *meso* forms (Dolinar *et al.*, 2018 ▸). Use of *XPREP* straightforwardly determines the space group *P*2_1_/*n* with *Z* = 6 (*Z*′= 1.5). Thus, one mol­ecule must reside on an inversion center as the *meso* stereoisomer. The structural solution is uneventful but requires paying attention to the amino protons. The mol­ecule on the general position is either *SS* or *RR*, depending on how the particular computer configuration affects the calculations, and typically an enlightening discussion ensues regarding how many different stereoisomers (three) are present in the crystal and in what *SS*:*RR*:*SR* ratio (1:1:1).

**Structure 6.** The structural work is preceded by an explanation that the organosilicon chemist characterized the compound by NMR spectroscopy and mass spectrometry prior to submitting crystals for structural analysis in an NMR tube. The experimentally established structure is a structural isomer of the proposed structure with an Si—H bond, but with the exact same mass and a proton NMR spectrum that *should* have been found inconsistent with the proposed structure. Once the structure of the chloro­form-*d* solvate of the naphthyl­silole is established (Timokhin *et al.*, 2006 ▸), we discuss how the NMR spectrum should look taking into account the presence of the hydro­silane proton: ^1^H NMR (CDCl_3_): δ = 4.56 (*s*, 1H, Si-H), 6.73–7.45 (*m*, 39H, Ar).

**Structure 7.** This project (Guzei & Treichel, 2025 ▸) is the first example in the course when the mol­ecular structure is not revealed in its entirety directly upon structure solution. Some of the atoms are assigned by the program, but the remainder of the structure must be built by identifying recognizable fragment geometries and differentiating between P, Cl, and O atoms over the course of several least-squares cycles. Additionally, the ionic pair crystallizes as a solvate with two chloro­form mol­ecules and solvent identification presents challenges. Most students have difficulty establishing the formal charge on the Re atoms, thus we reiterate the general procedure for this calculation: (*a*) remove all the ligands with the shared electron pairs from the metal atom(s); (*b*) calculate the total charge on the ligand(s) and counter-ion(s); (*c*) balance the charge by assigning appropriate positive charge(s) to the metal atom(s). This technique and the 18-electron rule are commonly used by organometallic chemists.

**Structure 8.** This structure serves as our gateway into positional disorder and the application of restraints and constraints (Guzei, 2025*a* ▸). Once the students identify the missing methyl group and the ammonium proton, they are challenged by a *SHELXL* message

**Possible inversion twin or centrosymmetric space group **

and its ramification for the relative abundance of the two stereoisomers sharing the same crystallographic site. One of the two chiral carbon atoms is disordered over two positions that correspond to different absolute configurations: *S* – 87%, *R* – 13%. The Hooft *y* parameter refines to 0.39 and therefore there are four different stereoisomers in the crystal with different relative abundances: *SS*: 0.87×0.61 = 0.53; *SR*: 0.13×0.61 = 0.08; *RR*: 0.87×0.39 = 0.34; *RS*: 0.13×0.39 = 0.05.

**Structure 9.** This is the most challenging structure of the set due to the completely incorrect proposed structure that resulted from a vial mix-up during the original sample submission. The mol­ecule contains no gold or oxygen atoms and resides on a crystallographic twofold axis, a fact that is easily missed. The structure refines well with Cl as the heaviest element, yet the full mol­ecule (with the second half generated by a 180° rotation) displays a Cl—Cl bond, and student crystallographers must think ‘like chemists’ to resolve this dilemma and identify a di­sulfide link. Due to the high data quality, all atomic identities can be reliably established (Keter *et al.*, 2009 ▸).

**Quartz.** This straightforward mineral example is used for a discussion of a high-symmetry structure (*P*3_2_21) and the difference between the absolute configuration and absolute structure.

**Structure 10.** This example simulates a situation when a colleague asks for help with a problematic structure. Thus, the *SHELXL* input and diffraction data files (RES and HKL) are already available and there is no need to solve the structure. We demonstrate the *OLEX2*’s SPLIT functionality for rotating an entire chemical group about an inter­atomic bond to model the second pyridine ring position and dive deeper into the use of restraints and free variables (Barbour, 2018*b* ▸).

**Structure 11.** An easy structure that gives the students a mid-semester boost in their confidence of independent structural analysis. This Mn^II^ complex is used to discuss the nature and typical geometrical parameters of hydrogen-bonding inter­actions, graph-set notation (Bernstein *et al.*, 1995 ▸), and to explore the rather remarkable mol­ecular packing. Our data matched the previously reported structure (Naumov *et al.*, 2008 ▸).

**Structure 12.** The Mn^V^ complex (Shields *et al.*, 2014 ▸) resides on an inversion center, a fact that can be deduced prior to the structure solution from the unit cell and mol­ecular volume calculations and the space-group selection. As the crystal symmetry exceeds the mol­ecular symmetry, the nitrido nitro­gen atom must be disordered over two positions. The students learn to handle elongated atomic displacement ellipsoids and partially occupied atoms on either side of the porphyrinato ring, and to refine disorder of both the nitride and metal atoms.

**Structure 13.** This is one of the few examples of a higher symmetry space group *I*
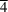
2*d* that students find particularly inter­esting. The Ni complex resides on a fourfold rotoinversion axis resulting in an aldehyde disorder (Spencer *et al.*, 2012 ▸) as the crystal symmetry exceeds the mol­ecular symmetry. The positional disorder of the aldehyde group manifests itself as a compositional disorder in the asymmetric unit. The challenge in modeling this disorder is to not only assign a 25% occupancy to the C, O, and H atoms of the aldehyde, but also not to forget about the 75% presence of the phenyl proton. The packing diagram generates O—O[sym] bonds between aldehyde groups that cannot be present at the same time in close proximity. We discuss why *OLEX2* and other programs draw bonds between atoms and how one can adjust atomic connectivity.

**Structure 14.** In the proposed structure all non-H atoms are carbon atoms. However in the actual structure some of them are oxygen and nitro­gen atoms (Guzei & Zimmerman, 2017 ▸). The high quality of the data enables the students to use their general chemical knowledge to establish the atomic identities and refine atomic occupancy factors for atoms that stand out in the difference-Fourier map.

**Structure 15.** The seemingly simple structure is an introduction to the atomic positional parameter (EXYZ) and atomic displacement parameter (EADP) constraints. The students must identify the special position on which the molecule resides, notice that there are Br atoms at the 4 and 5 imidazole positions, that these Br atoms are not fully occupied, discern a Cl/Br compositional disorder, examine C—Cl and C—Br distances, decide whether the use of EXYZ is warranted, and then propose what compounds may be present in the crystal. The Cl/Br disorder of the mol­ecule makes it possible for the Cl/Cl, Cl/Br, and Br/Br-substituted 2-methyl­imidazolidine to be present (Owczarek *et al.*, 2016 ▸). We discuss analytical methods to recommend to the crystal grower to determine the identity of the species present.

**Structure 16.** The objective is to learn when it is appropriate to use computational techniques for excluding certain parts of the structure from the structural model and compensating for their presence by modifying the structure factors [SQUEEZE procedure in *PLATON (*Spek, 2015 ▸) and *OLEX2.mask* procedure (Rees *et al.*, 2005 ▸) in *OLEX2*]. The students are presented with an incomplete structural model (RES file) of a Pd complex and asked to complete the refinement. The first challenge is to correctly assign the missing coordinated atom that appears to be the second oxygen atom of an acetate ligand, but is in fact a carbon atom. Several residual electron density peaks represent the potential solvent, and when modeling this mol­ecule proves too challenging the SQUEEZE and *OLEX2.mask* are employed and their results compared (Spencer *et al.*, 2025 ▸). We believe that it is important for students to understand what SQUEEZE and *OLEX2.mask* are, how they can be used most appropriately, and their limitations and pitfalls. Students use the mask to create a chemically reasonable and computationally stable model – in this case, a mol­ecular model is either not possible, or unjustifiably time-consuming. The CSD contains many structures with ‘squeezed out’ molecules: on October 27, 2025, a CSD search with the word ‘SQUEEZE’ returned 60,872 hits and a search with ‘MASK’ 22,197. We insist on documenting the use of these programs in the structural reports.

**Structure 17.** This example has two symmetry-independent Ti complexes and 0.25 of a solvent toluene molecule. This Ti complex was previously reported by Zaitsev *et al.* (2005 ▸). The toluene molecule is disordered over an inversion center and is most conveniently refined with a negative PART number. The negative number (*e.g.* PART −1) instructs the program to ignore the connectivity of the fragment to its symmetry-related mate, which makes H-atom placement straightforward. The students learn to use fragments from the Idealized Mol­ecular Geometry Library (IMGL; Guzei, 2025*b* ▸) and refine the solvent occupancy. Next, we employ the SQUEEZE and *OLEX2.mask* routines to exclude the solvent from the model and verify whether the electron count for the ‘squeezed’ solvent matches the count obtained for the model with the partially occupied toluene and discuss reasons for the differences.

**Structure 18.** This structural study was the focus of a paper describing the IMGL (Guzei, 2014*a* ▸). The solvated Pd complex is an excellent example of a PART −1 refinement for a hard-to-identify solvent mol­ecule. The difference-Fourier map contains four peaks in the asymmetric unit close to an inversion center, thus there are eight atomic positions for the solvent. The solvent is ethyl acetate with six non-H atoms, therefore only six of the Q peak positions are fully occupied, and the two terminal peaks are occupied 50% of the time. The suggested strategy is to use an idealized ethyl acetate mol­ecule from the IMGL to refine it with PART −1 and then remove the geometric constraint to allow the solvent geometry to refine.

**Structure 19.** The students are now ready to model disordered groups over more than two positions. The *SHELXL* commands SAME (geometrical restraints) and SUMP (linear restraint in an equation form) are introduced to achieve a stable refinement of the disordered CF_3_ group geometries and occupancies. The application of the SAME command is sometimes confusing and learning to use it correctly is imperative, because it is powerful when applied correctly and equally destructively powerful when applied incorrectly. This Cd complex (Metz *et al.*, 2004 ▸) is also a good candidate for an enhanced rigid-bond restraint, RIGU (Thorn *et al.*, 2012 ▸).

**Structure 20.** This host–guest structure requires the students to refine a *p*-methyl­phenol molecule disordered over a crystallographic inversion center using an idealized geometry and distinguishing between the hy­droxy and methyl groups. Similarly to Structure 18, an idealized mol­ecular geometry in PART −1 should be employed, but unlike Structure 18 this is a more troublesome case that usually requires more trials before the best structural model is built (Barbour, 2018*b* ▸).

**Structure 21.** This *p*-*tert*-butyl­calix[4]arene (Atwood *et al.*, 2004 ▸) with four disordered *tert*-butyl groups warrants the use of geometrical restraints (SAME) and atomic displacement parameter restraints (RIGU and possibly SIMU). The disorder model is created with convenient FIT and SPLIT tools in *OLEX2*. There is also a hydrogen-bonding motif formed by the four hy­droxy groups that can be refined with different hy­droxy hydrogen-atom settings. A discussion of the appropriate handling of these hydrogen atoms and hydrogen-bonding pattern nomenclature follows.

**Structure 22.** The structure of this Ru^II^/Ru^III^ complex (Roy *et al.*, 2022 ▸) solves easily and refines well provided the space group is chosen correctly – but this choice is far from clear-cut. The first step is to recognize that the unit cell is primitive rather than body-centered, and then to test three space groups consistent with the systematic absences: *P*4/*mnc*, *P*4*nc*, and *P*4/*nnc*. The *E*-statistics are misleading, which is not unusual when heavy elements occupy special positions. The students verify their space group choices and structural models by examining the correlation matrix element table in the *SHELXL* listing file (LST) and *PLATON*’s symmetry checks.

**Ferrocene.** The low-quality dataset offers mild space-group assignment challenges (the lattice is primitive rather than centered) and the direct methods fail to solve the structure in the correct space group *P*2_1_/*n*. However, prior to solving the structure one can deduce that only one half of the mol­ecule is symmetry-independent based on the unit cell and mol­ecular volume calculations, therefore the Fe atom must occupy an inversion center. As is sometimes the case with simple mineral structures in high-symmetry space groups, one does not *have to* solve the structure because the location of some atoms is known – the structure can be solved based on the known composition, symmetry elements, and inter­atomic distances. Placing the Fe atom at an inversion center obviates the need to solve the structure and the carbon atoms can be found subsequently in the difference-Fourier map after several least-squares cycles. The symmetry-independent cyclo­penta­dienyl ring is disordered over two positions, and a computationally stable refinement is achieved with an appropriate application of a geometrical constraint (penta­gon) and atomic displacement parameter restraints. The eclipsed (*D*_5*h*_, 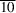
*m*2) and staggered (*D*_5*d*_, 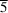

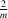
) ferrocene mol­ecules are excellent examples for demonstrating that deriving Hermann-Mauguin symbols is possible for ‘non-crystallographic’ point groups if one identifies three unique axial directions.

**Structure 23.** This Co complex (Hillbrands & Berry, 2019 ▸) structure contains three ‘catches’. The space group is non-centrosymmetric despite statistical indicators favoring a centrosymmetric one: it is *Cc* rather than the obvious *C*2/*c*. Secondly, the solvent water mol­ecules must be oriented correctly to optimize hydrogen-bonding inter­actions. The third one is less obvious – one must question the nature of the metal. Cobalt centers can be either Co^2+^ or Co^3+^, and under oxidative aqueous conditions they should be the latter. There are three symmetry-independent metal complexes and six chloride anions in the asymmetric unit, thus the formal metal oxidation state is 2+, which conflicts with the chemical expectation. A refinement of the metal-atom occupation factors indicates that the Co atoms should be replaced with a more electron-rich metal, Co’s neighbor in the periodic table, Ni. The high quality of the dataset makes it possible to identify the true nature of the metal. We discuss the use of statistical tools available in *ConQuest* (Bruno *et al.*, 2002 ▸) and *Mercury* (Macrae *et al.*, 2008 ▸) to effectively search the CSD to analyze and compare Co—N distances for six-coordinate Co^2+^ and Co^3+^ metal centers.

**Ylid twin.** The students are supplied with a non-merohedrally twinned dataset for 2-di­methyl­sufuranyl­idene-1,3-indanedione (ylid; Guzei *et al.*, 2008 ▸) and are advised on how to scale and correct the data for absorption with the program *TWINABS* (Sheldrick, 2012 ▸). Twin components are visualized with the Reciprocal Viewer plugin in program *APEX6* (Bruker, 2025 ▸). In this case, the contribution of the minor twin component is small and may be ignored.

**Structure 24.** The students are supplied with a good quality non-merohedrally twinned dataset, but in this case the minor component contribution is ∼35%. It is imperative to test two HKL files, one with a single-crystal dataset (HKLF 4) and one with diffraction data for both twin components (HKLF 5). During the *XPREP* run the space group cannot be found with the default settings; thus, the unit cell must be transformed to the standard setting for the space group *Pbcn*, either by inputting a manually derived transformation matrix, or by changing tolerances within *XPREP*. [For an example of tolerance adjustment in *XPREP* for a twinned crystal see, *e.g*. Guzei *et al.* (2012 ▸).] The structure solution and refinement against the HKLF 4 file proceed smoothly and reveal a starting material BPh_3_ characterized by Zettler *et al.* (1974 ▸). To refine the structure against the HKLF 5 file, a unit-cell transformation matrix is added to the HKLF 5 line, as the file was generated for the untransformed unit-cell setting. Unit-cell transformations are always good exercises for students, as are modifications to the INS file.

**Structure 25.** The monoclinic crystals of this Fe complex are pseudo-merohedrally twinned with *β* = 90°. The students are shown how to adjust tolerances in XPREP to obtain a unit cell list for lower symmetry crystal systems and how to select the likely unit cell by examining statistical indicators. Once the correct space group, *P*2_1_/*c*, is established, the structure may be solved but it does not refine well. We review the typical signs of twinning (Herbst-Irmer & Sheldrick, 1998 ▸), use of *PLATON* and *OLEX2* to check for twinning, and discuss the very similar axial lengths of *a* and *b* that make a 180° rotation about [001] a possible twin law. Once the twinning is accounted for, the improvement in the structural refinement indicators is dramatic. The actual structure with Fe^II^ and Fe^III^ centers in the cation and anion differs from the proposed one and matched a previously reported one by Giese *et al.* (2016 ▸).

**Structure 26.** This structure (Yakushev, 2025 ▸) introduces three important concepts. The first one is the unit-cell parameter precision. Only the HKL file is supplied, thus the user must type in the unit-cell parameters into *XPREP* manually. *XPREP* will generate unit-cell parameter uncertainties, but of course the experimentally determined ones should be used. We have previously reported that the realistic precision for axial length measurements is no better than 5 parts in 10,000, and 64 cycles of Monte Carlo simulations in *APEX6* produce very reasonable s.u.’s on the unit-cell parameters (Guzei *et al.*, 2008 ▸). Secondly, this dataset was collected at a synchrotron facility with a non-standard wavelength of 0.80246 Å, which must be specified in *XPREP*. Later, custom DISP cards with atomic scattering factors corresponding to this wavelength must be manually added to the INS file, because structure factors for non-typical wavelengths are not stored in *SHELXL*. Although making these entries is not difficult, they are not routine and warrant a demonstration as users may find them useful in the future. The third concept is the refinement of a ‘whole mol­ecule disorder’ due to the entire mol­ecule (all atoms in the asymmetric unit) being disordered over a crystallographic inversion center. The complete mol­ecule is refined with PART −1.

**Structure 27.** The proposed structure shows a four-coordinate Cr^I^ complex rather than the correct complex *L*_2_Cr^II^(THF) where *L* = 2,5-bis­{[(2,6-diiso­propyl­phen­yl)imino]­meth­yl}pyrrol-1-ide) with an octa­hedrally coordinated Cr^II^ center that is suspect right off the bat. The space-group selection and structural solution are routine, whereas the final model contains five electron density peaks overlapping with the coordinated THF. In order to identify the ‘mystery substituent’ it is advisable to read an accompanying paper (Salisbury *et al.*, 2022 ▸) that presents synthetic routes to similar compounds, where one of the chemicals used in such syntheses was LiCH_2_SiMe_3_. The five peaks would be consistent with –CH_2_SiMe_3_, and that is how the structure is reported to the CSD. Note that the presence of this ligand changes the oxidation state of the Cr center to Cr^III^. Alkyl­ation would oxidize the starting material, but it was originally thought that the oxidation did not occur at all. The structure proves that it does, but not qu­anti­tatively. It is educational to discuss the possibility of the ligand being –NHSiMe_3_, –OSiMe_3_, or HOSiMe_3_ and to examine the Cr—C/N/O and *E*—SiMe_3_ distances and compare them to the corresponding distances mined from the CSD. The coordinated *E* atom refines best as *E* = O, yet the Cr—*E* and *E*—Si distances match *E* = N best, whereas synthetically, *E* = C makes the most sense. This dataset demonstrates the limitations of modeling electron density and analyzing bond lengths in regions of compositional disorder.

**Structure 28.** This is a straightforward example of a non-merohedral twin with the two twin components related by a 180° rotation about [110] (Guzei *et al.*, 2025 ▸). The supplied data (the raw frames) are excellent, and the structure can be successfully refined either as a single crystal against an HKLF 4 file or as a twin against an HKLF 5 reflection file. The initial unit-cell determination with *CELL_NOW* (Sheldrick, 2008*a* ▸), a program for indexing (twinned) diffraction patterns, is a little thorny for two reasons: (*a*) the smallest unit cell dimension (4.91 Å) is shorter than the default axial lower limit of 5 Å, requiring a manual entry of the unit cell range; (*b*) the correct cell is listed as solution #24 with a figure of merit of 0.346 and the lowest percentage of fitting reflections (67.9%) among the 29 solutions printed in the DOS window. Most of the time the correct choice is the cell with the smallest volume regardless of the figure of merit, and this solution (*V* = 270 Å^3^) is a standout as other unit-cell volumes are close to 2157 Å^3^. This example expands the student experience with twinned structures and programs other than *SHELXL*.

**Structure 29.** This solvated Ru^I^ complex structure (Guzei & Casey, 2025 ▸) requires the use of statistical analysis tools available in *Mercury*. The structural solution and refinement are routine; however, the final difference-Fourier map indicates that one of the *ortho*-fluorine atoms is not fully occupied. Is it possible that it is an oxygen atom? The students then use CSD search tools to inter­rogate the database and statistically analyze C—F and C—OH bond distances, and to discuss what differences are statistically significant and whether it is sensible for this atom to be oxygen (which would be unprecedented based on the CSD searches). In the absence of other analytical information, the atom at the site of the partially occupied fluorine is modeled as a F/H compositional disorder. There is also a solvent mol­ecule of toluene disordered over two positions in a ∼62:38 ratio. The IMGL is instrumental for imposing constrained geometries on these mol­ecules. We also use the *OLEX2.mask* procedure to ‘squeeze’ the solvent out to estimate the number of electrons at the solvent site. The procedure calculates 46 electrons for the diffusely diffracting species rather than the expected 50 and we discuss whether the solvent occupancy should be adjusted to 92%.

**Structure 30.** The Ni^II^ complex structure exemplifies a positional disorder manifested as compositional disorder between the propyl chain and the O/OH atoms due to the mol­ecule residing on a mirror plane (Chang *et al.*, 2022 ▸). It is possible to locate the electron density peaks corresponding to all five non-hydrogen atoms and model the disorder using restraints. We take the opportunity to explain that the space group *Cmca* assigned in *XPREP* is displayed as *Cmce* in *OLEX2* due to the 1992 IUCr change of symbol for glide planes with translation components along two axes to ***e***.

**Structure 31.** This hexa­nuclear Pd^0^ complex (Ivanov *et al.*, 2023 ▸) requires the crystallographer to explore possible twinning with *PLATON* and then properly account for it. The catch is that identifying the twin law in *PLATON* and inserting the

TWIN −1 0 0 0 −1 0 0.085 0 1

BASF 0.12

cards into the INS file does not substanti­ally affect the refinement. Instead, an HKLF 5 file should be generated from within *PLATON*. A refinement against the twinned HKLF 5 file improves the residual *R*1 from ∼0.055 to ∼0.039 and the largest peak in the difference-Fourier map is reduced from ∼4.1 to ∼1.5 e Å^−3^. We also use this example to explore and compare twin identification tools available in *OLEX2*.

**Structure 32.** The students are supplied with a CIF and are shown how to extract the diffraction data and instruction file inside *OLEX2*. The observed structure has nothing in common with the reaction scheme supplied by the chemist, and does not make chemical sense with the main mol­ecule refined as O=BrPh_3_. The students must establish the identity of the ‘bromine’ atom, and whereas most chemists are familiar with tri­phenyl­phosphine oxide, few have encountered tri­phenyl­arsine oxide, hence the challenge. The structure refines very well with a compositional As/P 0.88/0.12 disorder. There is also a solvent water mol­ecule present in the asymmetric unit and we revisit the hydrogen-bonding-pattern notation, which in this case is a ring *R*^2^_4_(8).

### The two pillars of crystal analysis: description and discussion

Throughout the practical sessions we emphasize the core distinction between structural description (the ‘what’) and structural discussion (the ‘why’ and ‘why it matters’). The former focuses on the objective reporting of features including unit-cell parameters, mol­ecular symmetry, mol­ecular conformation (*e.g.*, ring puckering, metal coordination geometry), twinning, disorder/solvent occupancy, and supra­molecular features such as hydrogen bonding or π-stacking inter­actions. We adhere to IUCr journal guidelines on reporting all numerical values with standard uncertainties (s.u.) or estimated standard deviation (e.s.d.) including strict application of the 2–19 rule (see *Notes for Authors*). Structural discussion focuses on chemical inter­pretation and critical evaluation. It aims to explain important structural features (*e.g.*, proton transfer or presence of stereoisomers), rationalize geometric variations (*e.g*., ligand *trans* effects or deviations from ideal bond angles), and correlate these features with physical properties (*e.g.* stability, reactivity, solubility). Ultimately, the discussion assesses the significance of the study, determining whether synthetic expectations were met, an unexpected transformation occurred, or a new chemical insight was obtained. In instances where a definite answer cannot be deduced, we discuss supplementary structural or analytical techniques that could provide an explanation. Over the course of these sessions, students participate in numerous ‘post structure determination’ analyses and develop the vocabulary and skills necessary to competently present their independent structural studies. To drive home the necessary convergence of chemical and crystallographic inter­pretation, one of the final 50-minute lectures explores case studies in which crystallographic data contained mistakes or were used to support chemically inaccurate results, using examples from Baur & Kassner (1992 ▸), Burrell *et al.* (1995 ▸), Cotton *et al.* (2001 ▸), Harlow (1996 ▸), Kersten *et al.* (1992 ▸), Marsh & Clemente (2007 ▸), O’Halloran *et al.* (2012 ▸), Parkin (1993 ▸), and Raymond & Girolami (2023 ▸). These examples serve as cautionary tales to remind students to always think critically about their results.

## Conclusions

We present our vision for the practical component of the graduate-level Chemical Crystallography course at the University of Wisconsin–Madison. We have outlined our preferred resources, including essential crystallographic texts, software, websites, in-class problem sets, and home assignments. A significant focus is placed on 37 hands-on weekly exercises involving structures of varied difficulty. This heavy emphasis on X-ray single-crystal diffractometry and data inter­pretation equips students with the skills needed to perform independent structural investigations and critically evaluate crystallographic literature.

**Conflicts of interest** The authors declare no conflict of inter­est.

The diffraction data and concomitant materials for the 37 structures outlined in Figs. 4 ▸ and 5 ▸ are available for download free of charge from https://xray.chem.wisc.edu/crystallographic-problems/. Other materials are available as outlined in the text and references.

## Supplementary Material

Diffraction data for the exercises. DOI: 10.1107/S2056989025010527/oi2027sup2.zip

Supporting information file. DOI: 10.1107/S2056989025010527/oi2027sup3.zip

Crystallographic experiment index cards. DOI: 10.1107/S2056989025010527/oi2027sup4.pdf

## Figures and Tables

**Figure 5:**
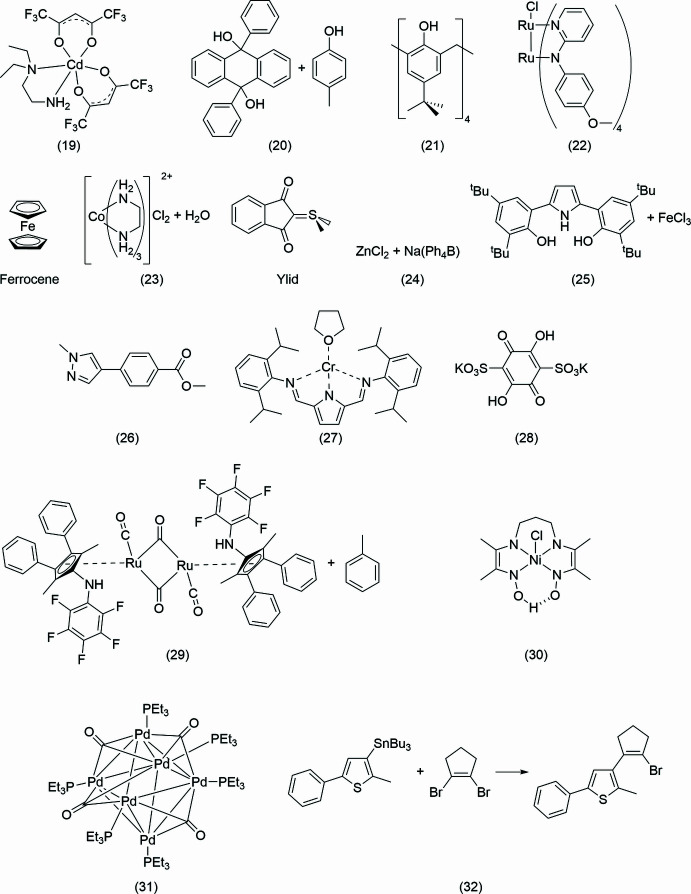
More chemical drawings of the proposed structures for the Chemical Crystallography course.

**Table 1:** Description of symmetry exercises for the Chemical Crystallography class. The exercises used in Spring 2025 are included in the supporting information

Exercise	Topic	Comments
Symmetry 1	Symmetry elements	Students are asked to identify symmetry elements in objects.
Symmetry 2	Point groups	Students identify the point group depicted by a set of objects arranged around a central point.
Symmetry 3	Ribbon patterns	Students categorize ribbons according to their frieze symmetry, which introduces them to translations and compound symmetry elements.
Symmetry 4	Wallpaper patterns	Students are asked to determine the plane groups present in wallpaper patterns.
Symmetry 5	Transformations	The operation of various symmetry elements on a general point (*xyz*) is explored.
Symmetry 6	Space groups	Given either a symmetry element diagram or an equivalent point diagram for a particular space group, students are asked to generate the other type of diagram.
Symmetry 7	More space groups	Students determine the space group represented by a group of objects arranged within a unit cell.
Symmetry 8	More transformations	Students are explicitly asked to use matrices to solve symmetry problems.

**Table 2:** Diffraction and reciprocal space exercises for the Chemical Crystallography class. The exercises used in Spring 2025 are included in the supporting information

Exercise	Topic	Comments
Directions and Planes	Crystallographic directions [*uvw*] and planes (*hkl*)	Familiarizes students with the notation of directions and planes in crystallography.
Powder Diffraction	Pattern indexing	Given a set of diffraction angles, students are asked to index a cubic powder pattern.
Structure Factors 1	Calculation of structure factors from a known structure	Students are given atomic coordinates and form factors for a simple structure and are asked to calculate *F* for a specific *hkl* reflection.
Structure Factors 2	Relationships between structure factors	Students are asked to determine the relationship between structure factors of atoms related by certain symmetry elements.
Structure Factor 3	Reflection conditions	Students are asked to determine which classes of *F*(*hkl*) are absent when certain symmetry elements are present.
Data Analysis	Space-group determination	Students are provided with zone images from real datasets from which they are asked to determine the space group of the crystal.
Direct Methods	Phasing of reflections	Students are provided with a set of reflections for a centrosymmetric structure and their *E* (normalized structure factors) values, and are asked to determine phases for as many reflections as possible.
