# Supplementary material for: Practical aspects of teaching a graduate-level small-mol­ecule chemical crystallography course
Source: Acta Crystallogr E Crystallogr Commun. 2026 Jan 1;82(Pt 1):107–20. doi: 10.1107/S2056989025010527 (PMC12810306; doi:10.1107/S2056989025010527)

checkCIF/PLATON report

Structure factors have been supplied for datablock(s) o088

THIS REPORT IS FOR GUIDANCE ONLY. IF USED AS PART OF A REVIEW PROCEDURE FOR PUBLICATION, IT SHOULD NOT REPLACE THE EXPERTISE OF AN EXPERIENCED CRYSTALLOGRAPHIC REFEREE.

No syntax errors found. CIF dictionary Interpreting this report

Datablock: o088

```
Bond precision:  C-C = 0.0041 A         Wavelength=1.54184

Cell:           a=9.45574(7)    b=16.26721(10)      c=11.06992(8)
                alpha=90        beta=113.5838(8)    gamma=90
Temperature:    126 K

                Calculated                Reported
Volume          1560.54(2)                1560.54(2)
Space group     P 21/n                    P 1 21/n 1
Hall group      -P 2yn                    -P 2yn
Moiety formula  C18 H15 Br O, H2 O        C18 H15 Br O, H2 O
Sum formula     C18 H17 Br O2             C18 H17 Br O2
Mr              345.22                    345.22
Dx,g cm-3       1.469                     1.469
Z               4                         4
Mu (mm-1)       3.598                     3.598
F000            704.0                     704.0
F000'           702.92
h,k,lmax        11,19,13                  11,19,13
Nref            2777                      2774
Tmin,Tmax       0.446,0.587               0.832,0.889
Tmin'           0.404

Correction method= # Reported T Limits: Tmin=0.832 Tmax=0.889
AbsCorr = ANALYTICAL

Data completeness= 0.999          Theta(max)= 66.766

R(reflections)= 0.0286( 2736)     wR2(reflections)= 0.0895( 2774)

S = 1.112                  Npar= 193
```

The following ALERTS were generated. Each ALERT has the format
test-name_ALERT_alert-type_alert-level.
Click on the hyperlinks for more details of the test.

Alert level C

```
PLAT230_ALERT_2_C Hirshfeld Test Diff for    Br01   --  O00     ..        5.9 su
PLAT230_ALERT_2_C Hirshfeld Test Diff for    Br01   --  C006    ..        5.4 su
PLAT911_ALERT_3_C Missing # FCF Refl Between THmin & STh/L=  0.596          3 Report
```

Alert level G

```
PLAT007_ALERT_5_G Number of Unrefined Donor-H Atoms .............          2 Report
PLAT142_ALERT_4_G su on b - Axis Small or Missing ...............    0.00010 Ang.
PLAT143_ALERT_4_G su on c - Axis Small or Missing ...............    0.00008 Ang.
PLAT720_ALERT_4_G Number of Unusual/Non-Standard Labels ..........        38 Note
PLAT909_ALERT_3_G Percentage of Observed Data at Theta(Max) still         97 %
PLAT961_ALERT_5_G Dataset Contains no Negative Intensities .......     Please Check
```

```
   0 ALERT level A = Most likely a serious problem - resolve or explain
   0 ALERT level B = A potentially serious problem, consider carefully
   3 ALERT level C = Check. Ensure it is not caused by an omission or oversight
   6 ALERT level G = General information/check it is not something unexpected

   0 ALERT type 1 CIF construction/syntax error, inconsistent or missing data
   2 ALERT type 2 Indicator that the structure model may be wrong or deficient
   2 ALERT type 3 Indicator that the structure quality may be low
   3 ALERT type 4 Improvement, methodology, query or suggestion
   2 ALERT type 5 Informative message, check
```

It is advisable to attempt to resolve as many as possible of the alerts in all categories. Often the minor alerts point to easily fixed oversights, errors and omissions in your CIF or refinement strategy, so attention to these fine details can be worthwhile. In order to resolve some of the more serious problems it may be necessary to carry out additional measurements or structure refinements. However, the purpose of your study may justify the reported deviations and the more serious of these should normally be commented upon in the discussion or experimental section of a paper or in the "special_details" fields of the CIF. checkCIF was carefully designed to identify outliers and unusual parameters, but every test has its limitations and alerts that are not important in a particular case may appear. Conversely, the absence of alerts does not guarantee there are no aspects of the results needing attention. It is up to the individual to critically assess their own results and, if necessary, seek expert advice.

Publication of your CIF in IUCr journals

A basic structural check has been run on your CIF. These basic checks will be run on all CIFs submitted for publication in IUCr journals (*Acta Crystallographica*, *Journal of Applied Crystallography*, *Journal of Synchrotron Radiation*); however, if you intend to submit to *Acta Crystallographica Section C* or *E*, you should make sure that full publication checks are run on the final version of your CIF prior to submission.

Publication of your CIF in other journals

Please refer to the *Notes for Authors* of the relevant journal for any special instructions relating to CIF submission.

PLATON version of 21/06/2015; check.def file version of 21/06/2015

Datablock o088 - ellipsoid plot

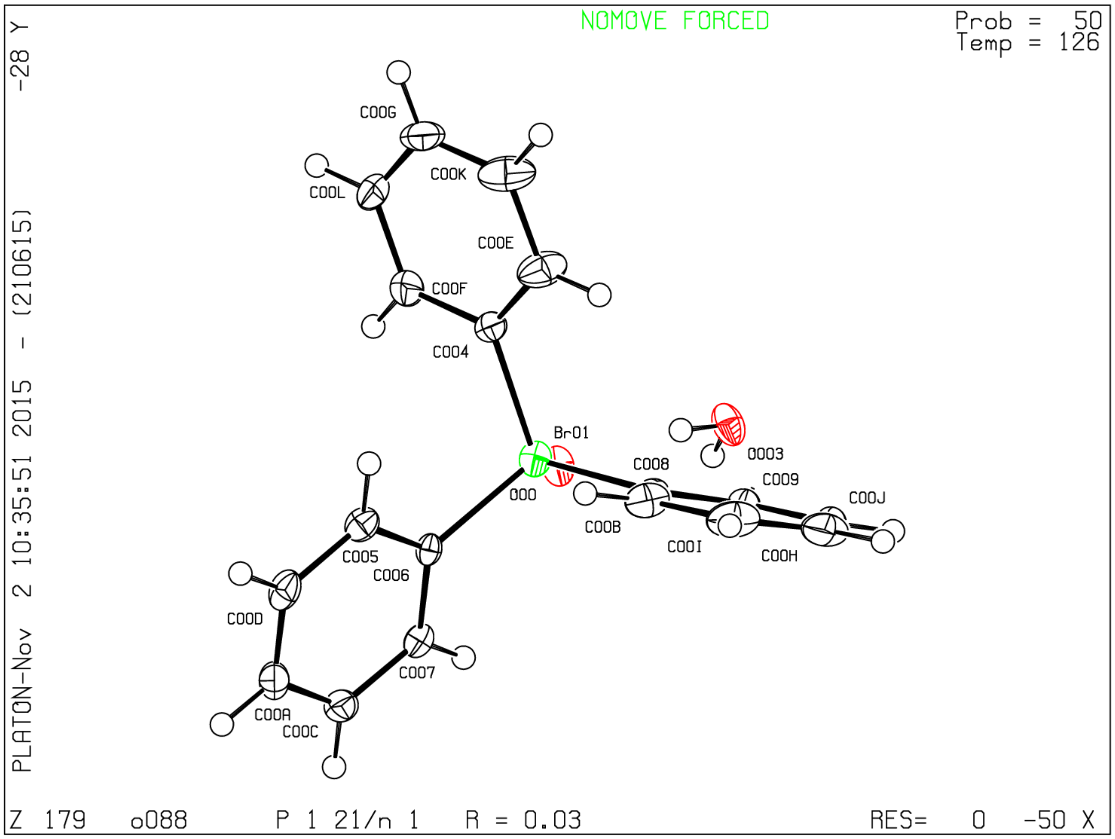

Supplement: Supplementary file 1 [file e-82-00107-sup2.zip › Berry_Guzei_Structures/Structure 32/Structure32.pdf]
