# Supplementary figures and images for: Practical aspects of teaching a graduate-level small-mol­ecule chemical crystallography course
Source: Acta Crystallogr E Crystallogr Commun. 2026 Jan 1;82(Pt 1):107–20. doi: 10.1107/S2056989025010527 (PMC12810306; doi:10.1107/S2056989025010527)

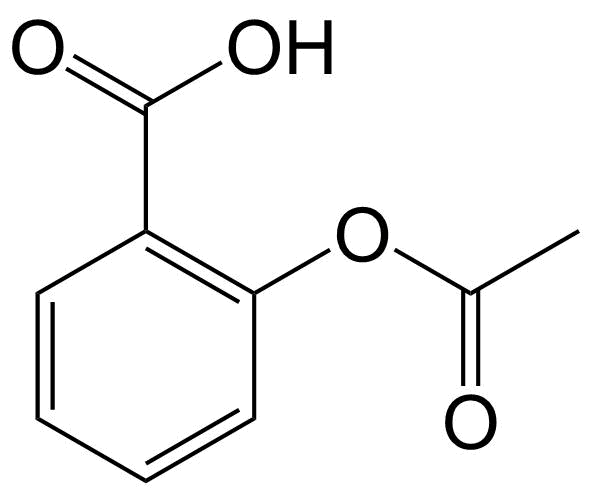

Supplement: Supplementary file 1 [file e-82-00107-sup2.zip › Berry_Guzei_Structures/Aspirin/Aspirin.png]

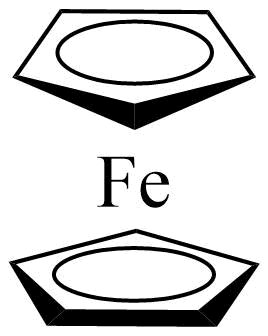

Supplement: Supplementary file 1 [file e-82-00107-sup2.zip › Berry_Guzei_Structures/Ferrocene/Ferrocene.png]

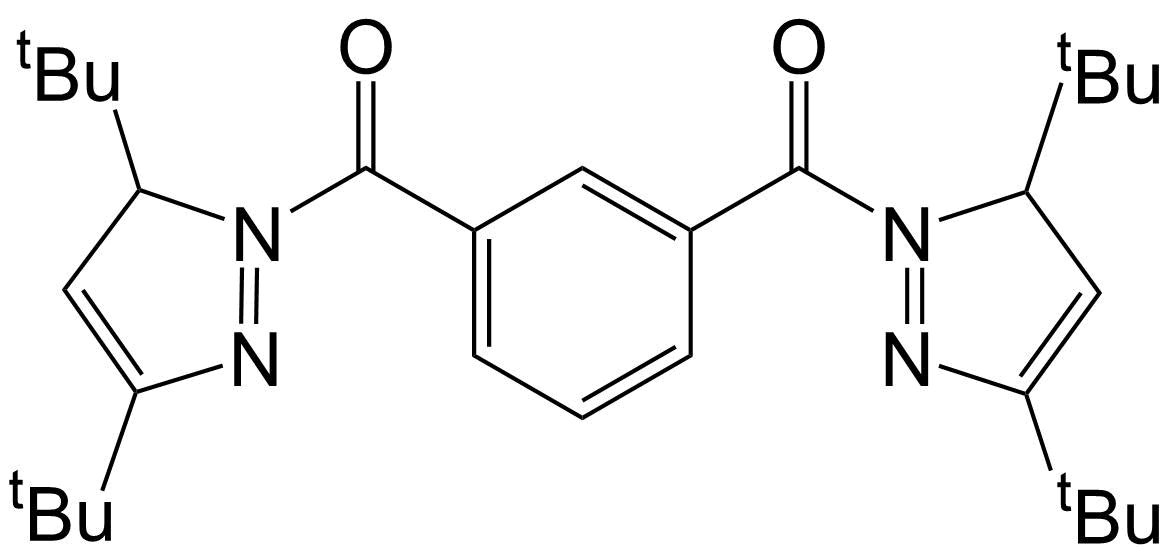

Supplement: Supplementary file 1 [file e-82-00107-sup2.zip › Berry_Guzei_Structures/Structure 1/Structure1.png]

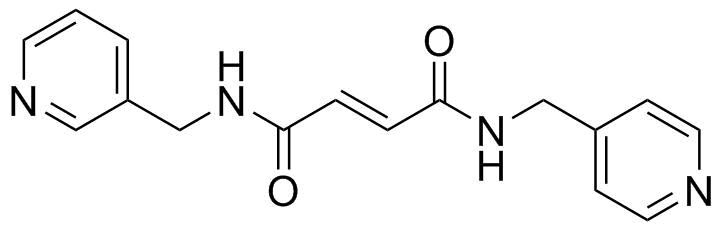

Supplement: Supplementary file 1 [file e-82-00107-sup2.zip › Berry_Guzei_Structures/Structure 10/structure10.png]

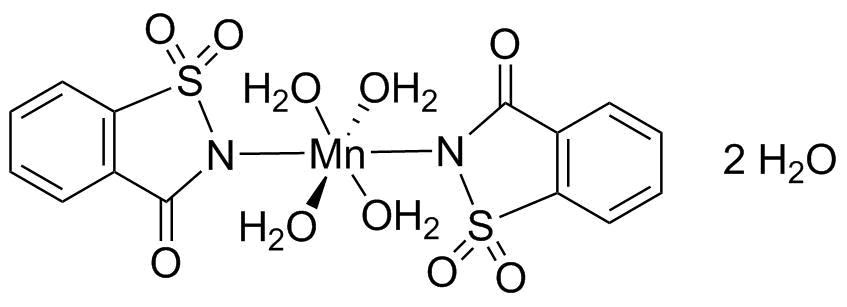

Supplement: Supplementary file 1 [file e-82-00107-sup2.zip › Berry_Guzei_Structures/Structure 11/structure11.png]

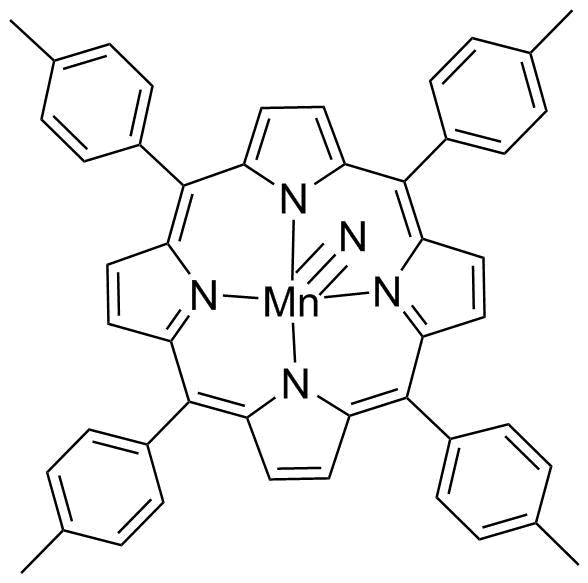

Supplement: Supplementary file 1 [file e-82-00107-sup2.zip › Berry_Guzei_Structures/Structure 12/structure12.png]

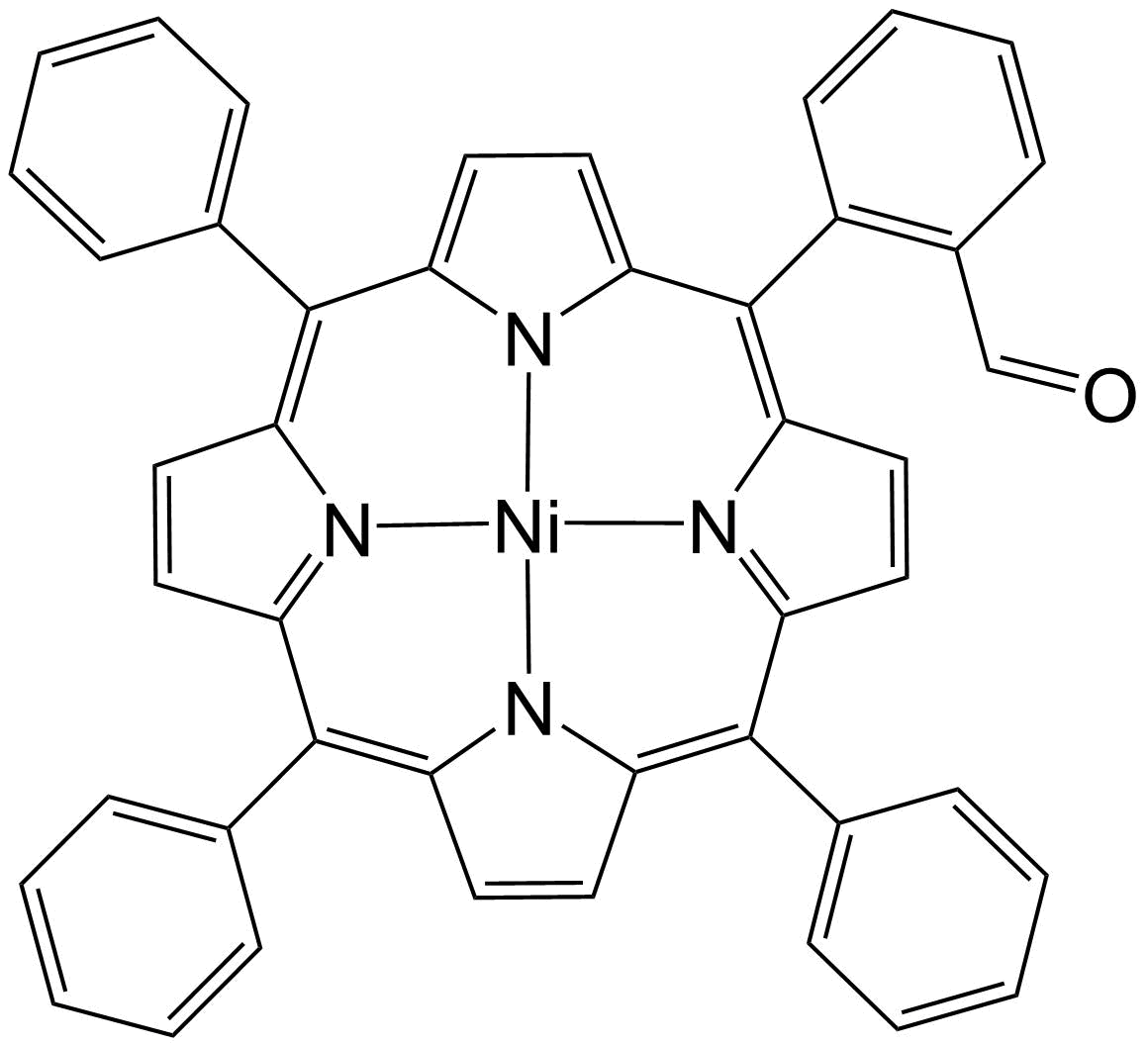

Supplement: Supplementary file 1 [file e-82-00107-sup2.zip › Berry_Guzei_Structures/Structure 13/Structure13.png]

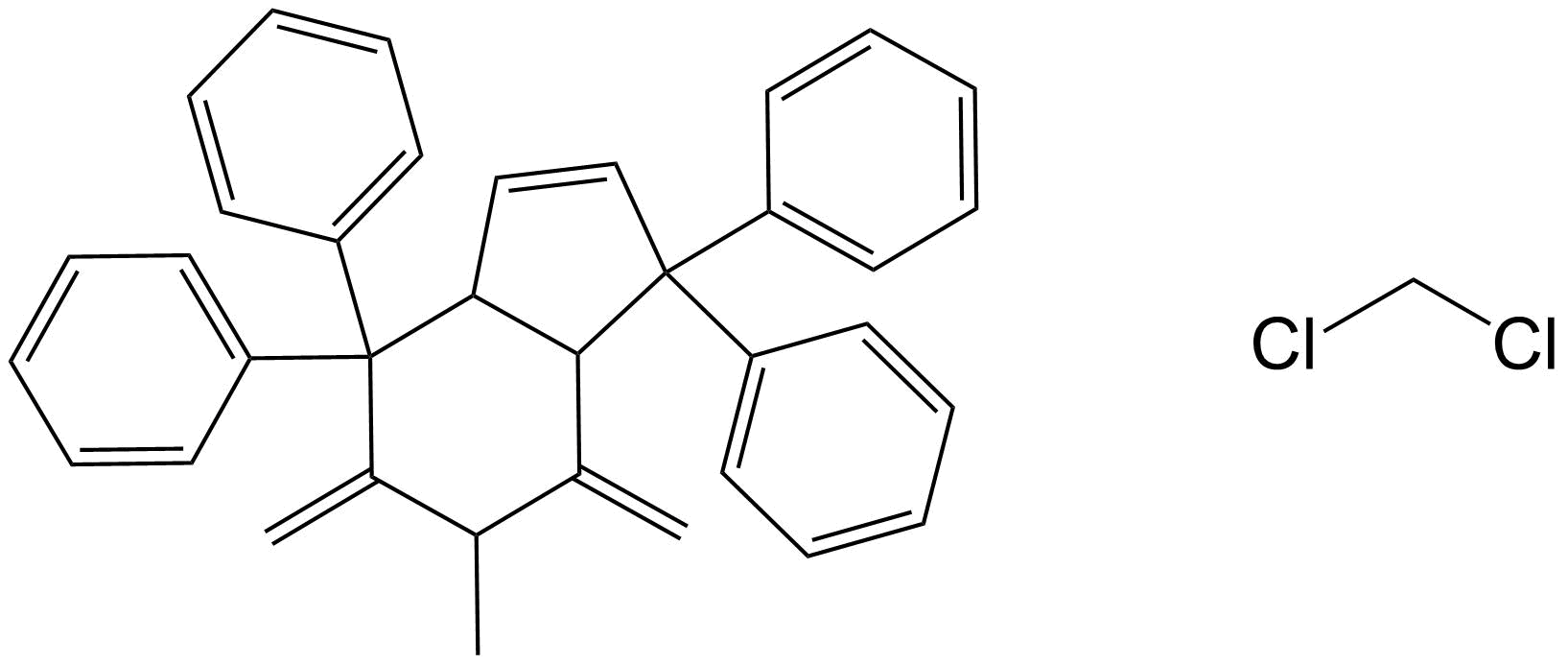

Supplement: Supplementary file 1 [file e-82-00107-sup2.zip › Berry_Guzei_Structures/Structure 14/structure14.png]

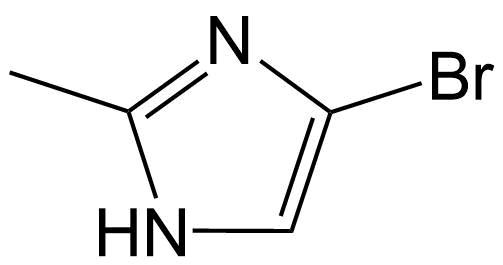

Supplement: Supplementary file 1 [file e-82-00107-sup2.zip › Berry_Guzei_Structures/Structure 15/structure15.png]

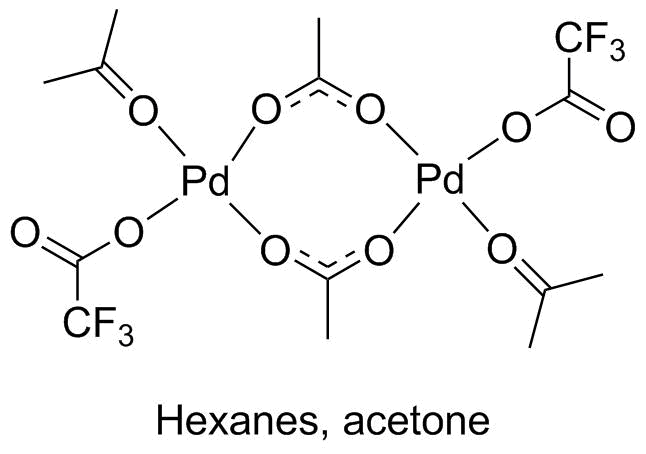

Supplement: Supplementary file 1 [file e-82-00107-sup2.zip › Berry_Guzei_Structures/Structure 16/structure16.png]

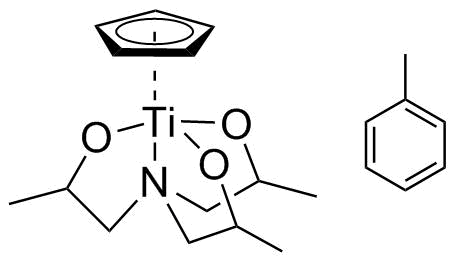

Supplement: Supplementary file 1 [file e-82-00107-sup2.zip › Berry_Guzei_Structures/Structure 17/structure17.png]

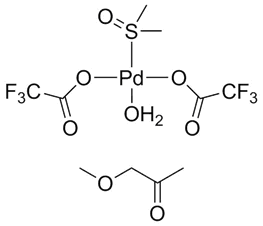

Supplement: Supplementary file 1 [file e-82-00107-sup2.zip › Berry_Guzei_Structures/Structure 18/structure18.png]

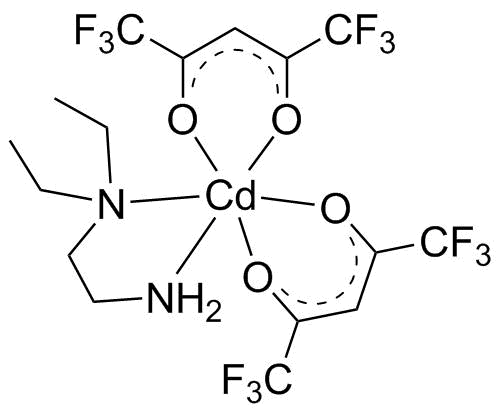

Supplement: Supplementary file 1 [file e-82-00107-sup2.zip › Berry_Guzei_Structures/Structure 19/structure19.png]

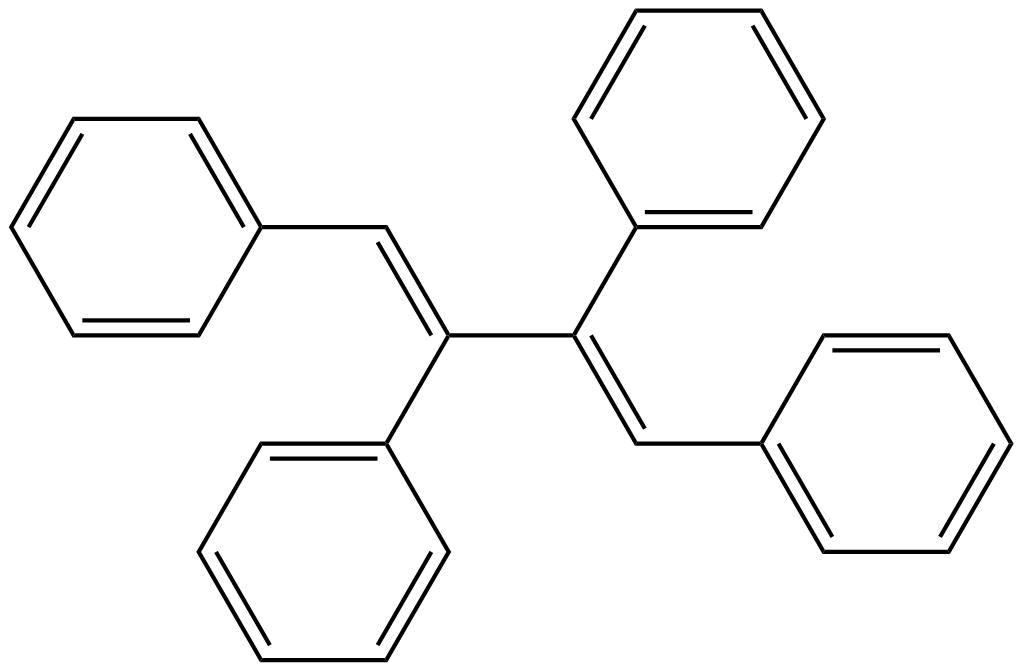

Supplement: Supplementary file 1 [file e-82-00107-sup2.zip › Berry_Guzei_Structures/Structure 2/Structure2.png]

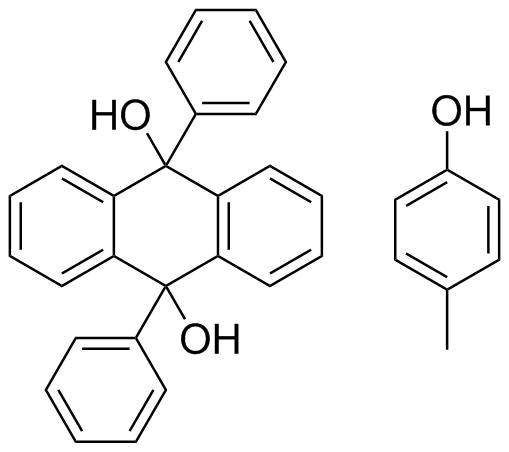

Supplement: Supplementary file 1 [file e-82-00107-sup2.zip › Berry_Guzei_Structures/Structure 20/structure20.png]

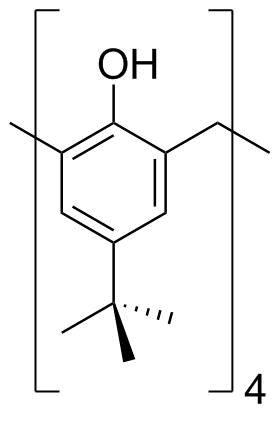

Supplement: Supplementary file 1 [file e-82-00107-sup2.zip › Berry_Guzei_Structures/Structure 21/structure21.png]

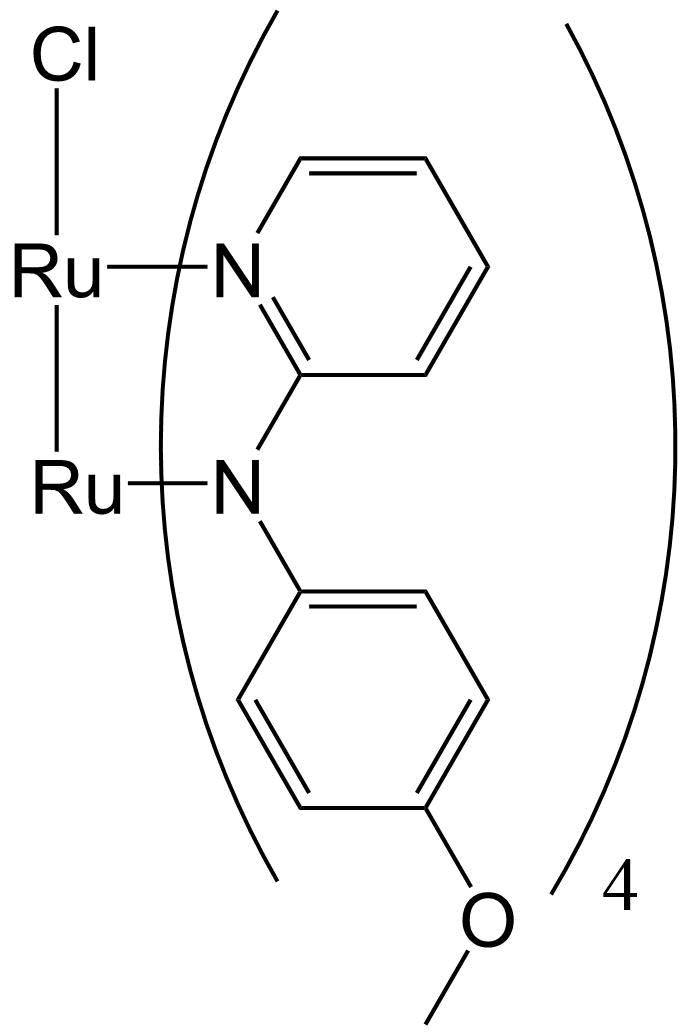

Supplement: Supplementary file 1 [file e-82-00107-sup2.zip › Berry_Guzei_Structures/Structure 22/structure22.png]

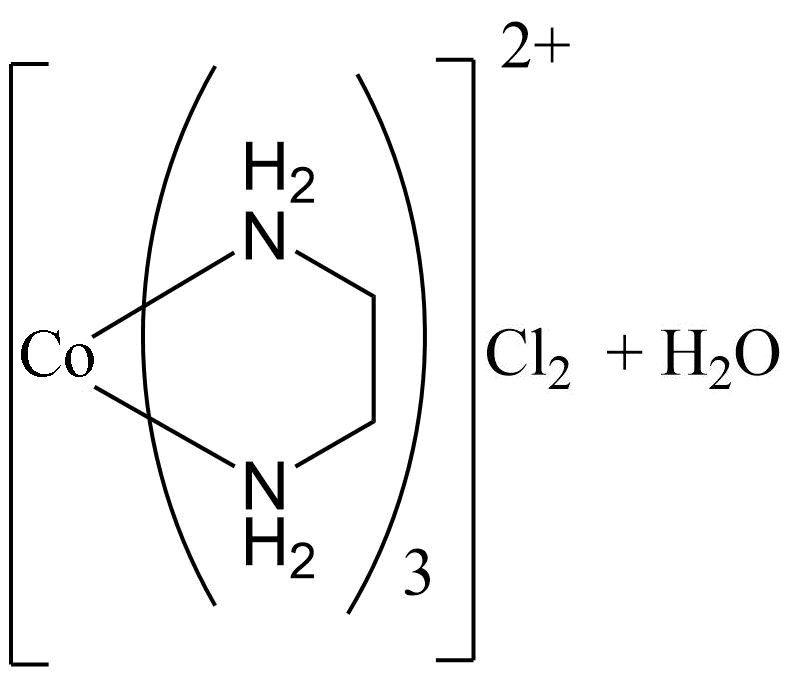

Supplement: Supplementary file 1 [file e-82-00107-sup2.zip › Berry_Guzei_Structures/Structure 23/structure23.png]

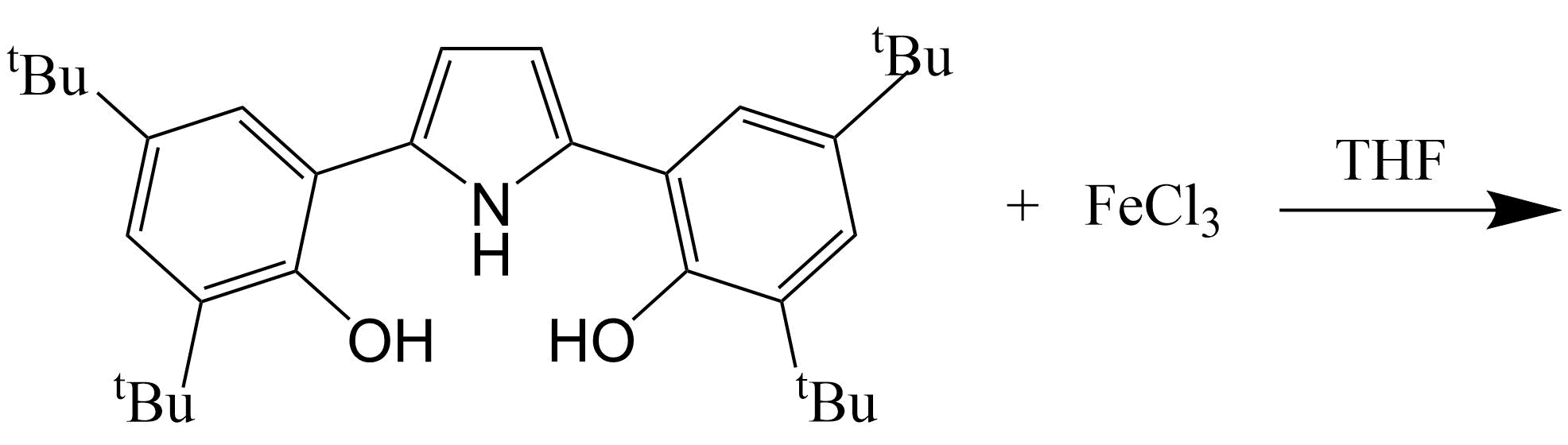

Supplement: Supplementary file 1 [file e-82-00107-sup2.zip › Berry_Guzei_Structures/Structure 25/Structure25.png]

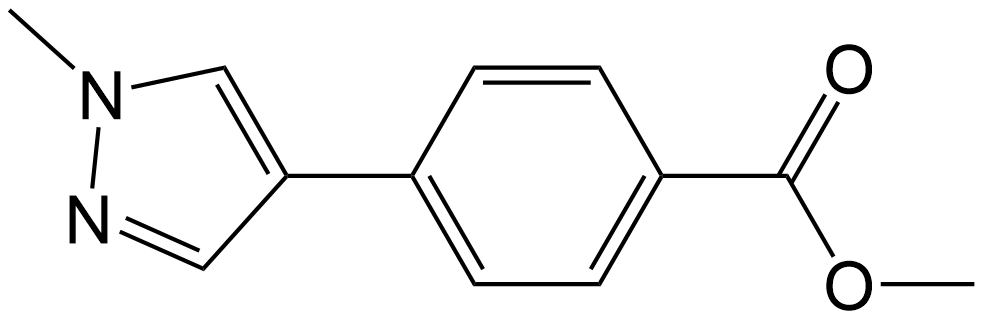

Supplement: Supplementary file 1 [file e-82-00107-sup2.zip › Berry_Guzei_Structures/Structure 26/Structure26.png]

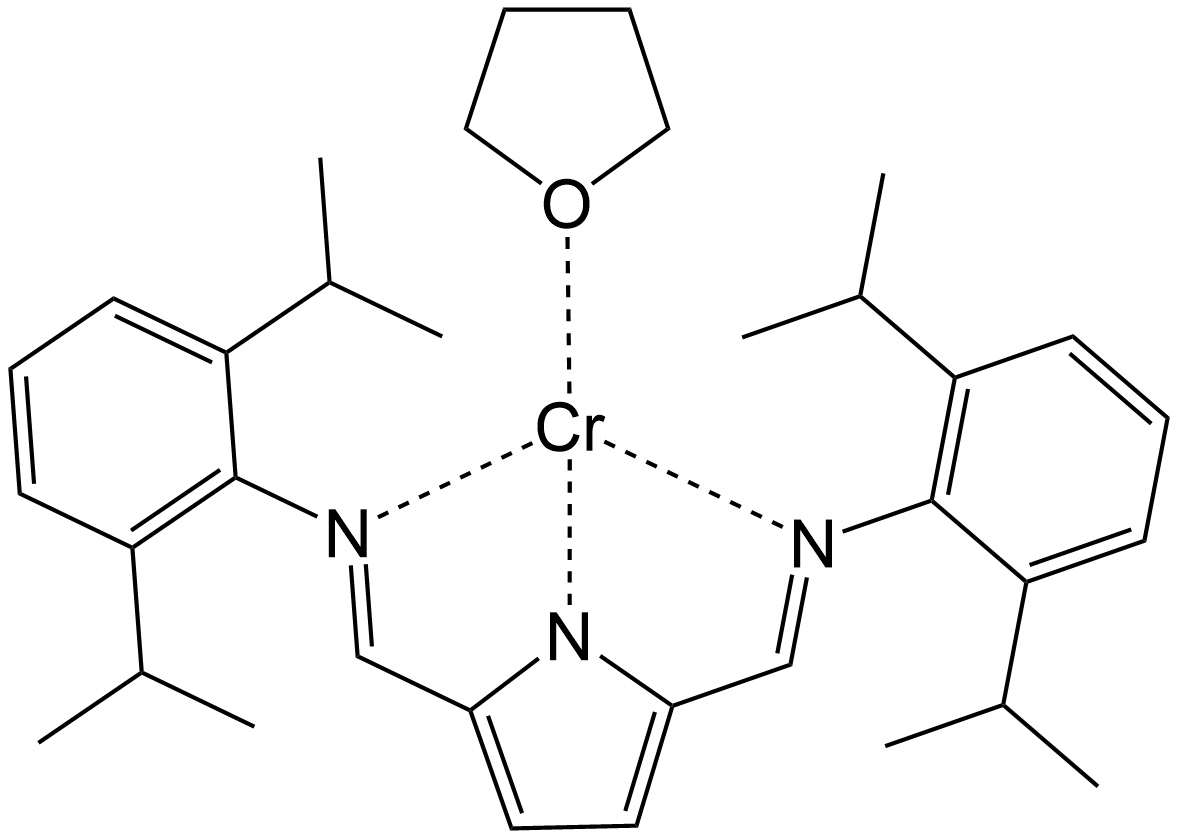

Supplement: Supplementary file 1 [file e-82-00107-sup2.zip › Berry_Guzei_Structures/Structure 27/Structure27.png]

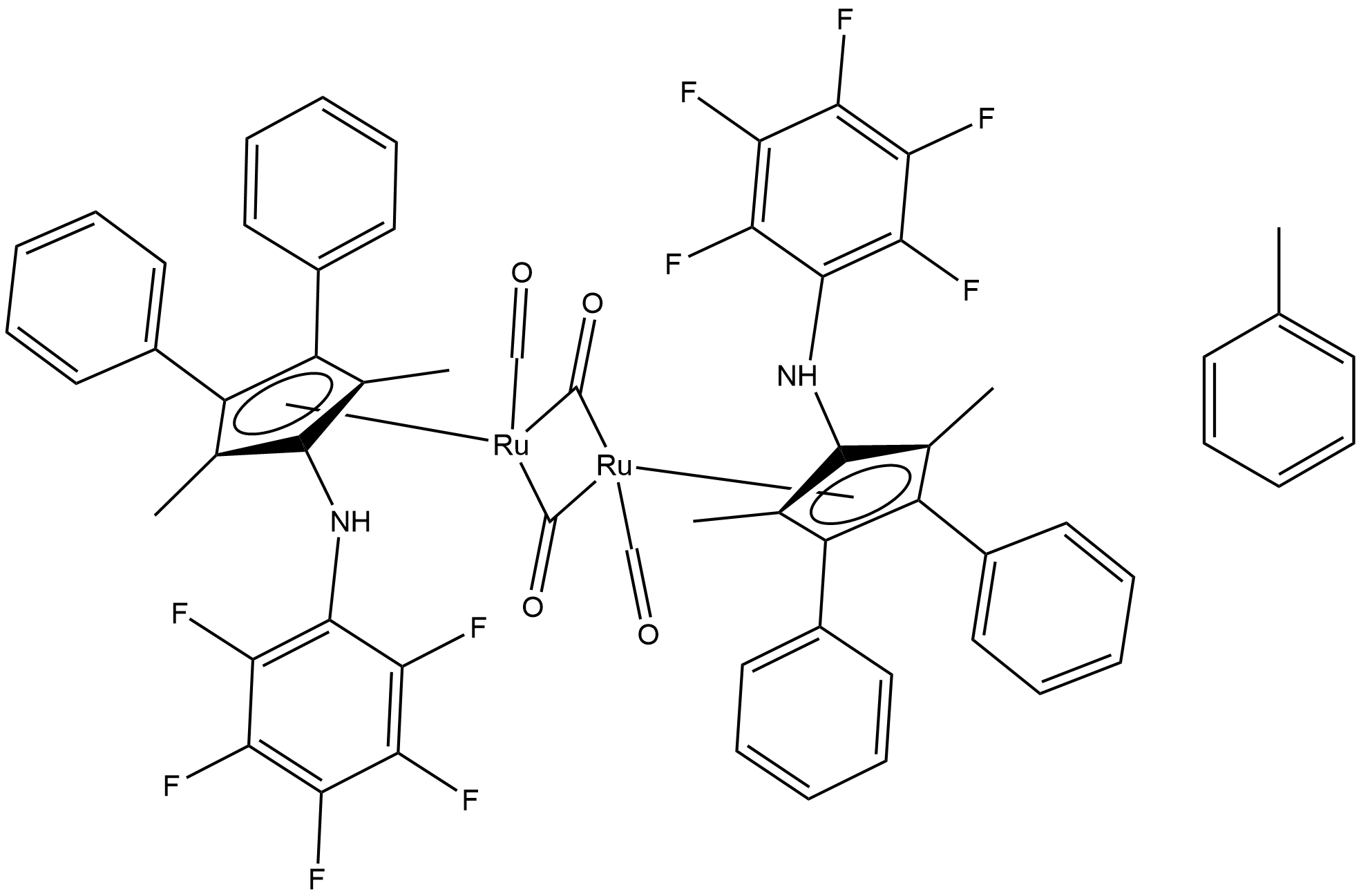

Supplement: Supplementary file 1 [file e-82-00107-sup2.zip › Berry_Guzei_Structures/Structure 29/Structure29.png]

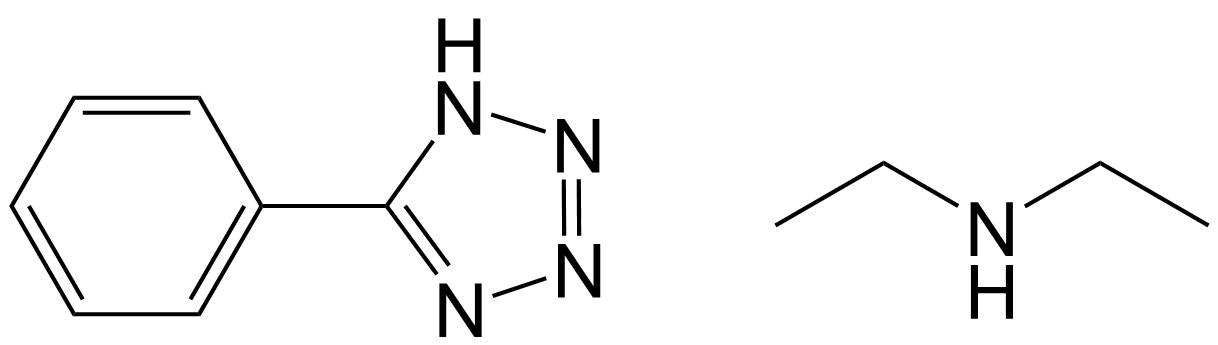

Supplement: Supplementary file 1 [file e-82-00107-sup2.zip › Berry_Guzei_Structures/Structure 3/Structure3.png]

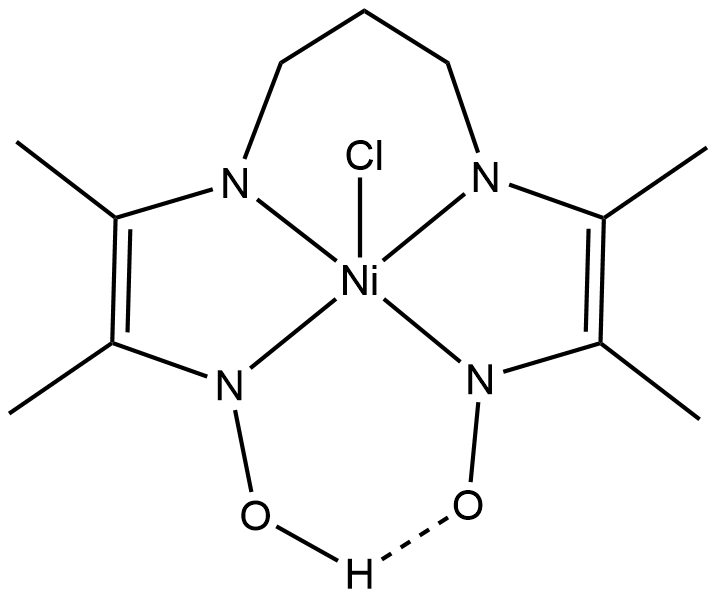

Supplement: Supplementary file 1 [file e-82-00107-sup2.zip › Berry_Guzei_Structures/Structure 30/Structure30.png]

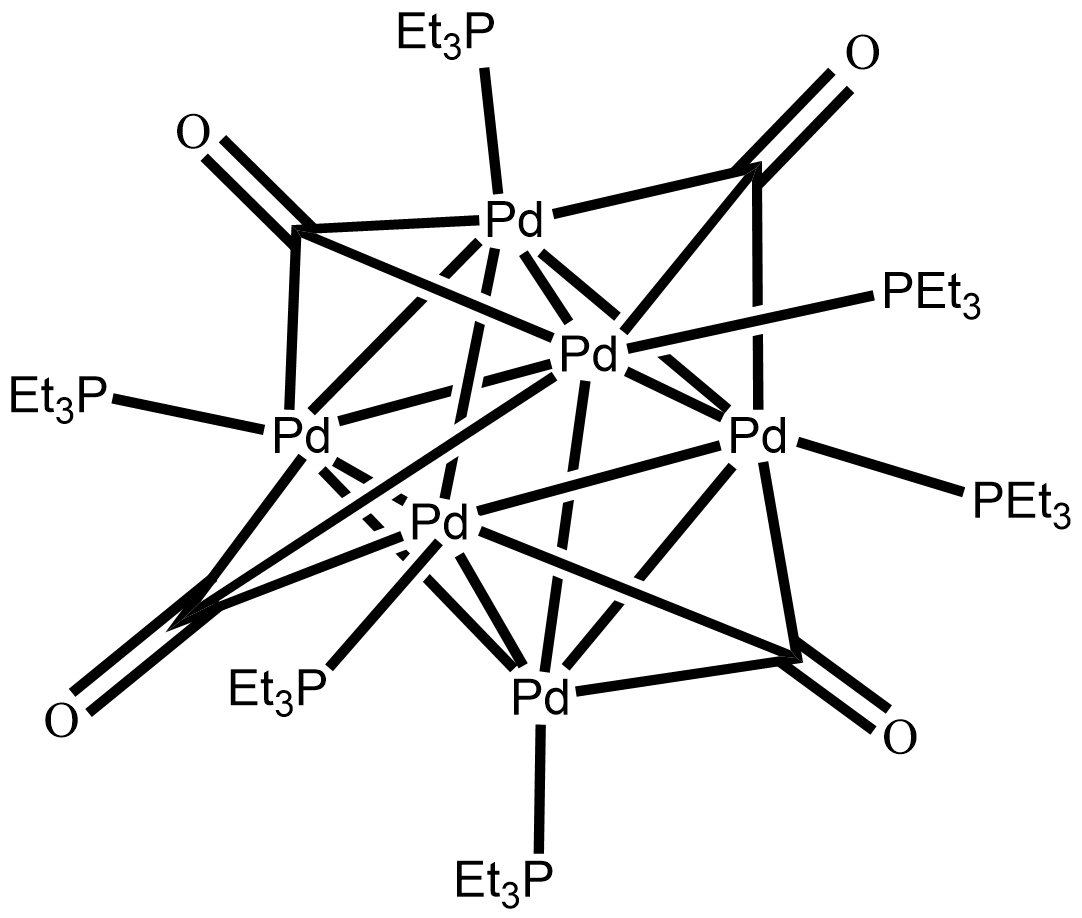

Supplement: Supplementary file 1 [file e-82-00107-sup2.zip › Berry_Guzei_Structures/Structure 31/Structure31.png]

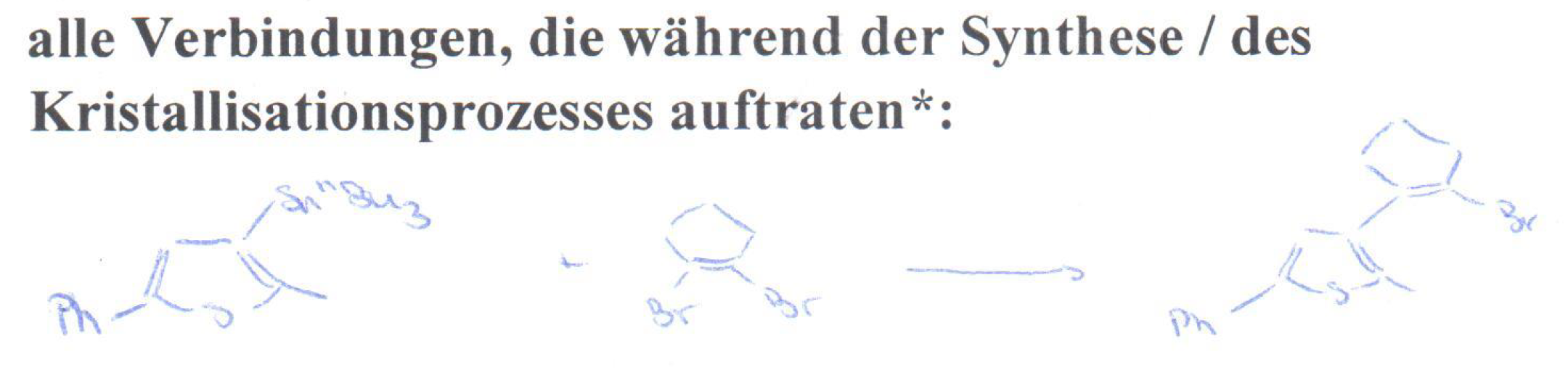

Supplement: Supplementary file 1 [file e-82-00107-sup2.zip › Berry_Guzei_Structures/Structure 32/Structure32.PNG]

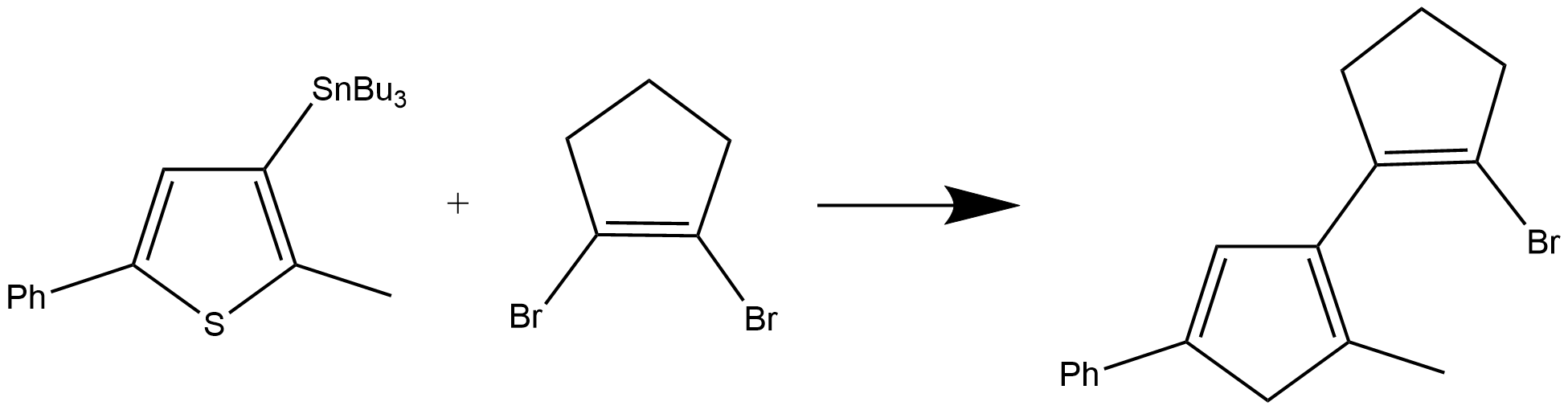

Supplement: Supplementary file 1 [file e-82-00107-sup2.zip › Berry_Guzei_Structures/Structure 32/Structure32_reaction.PNG]

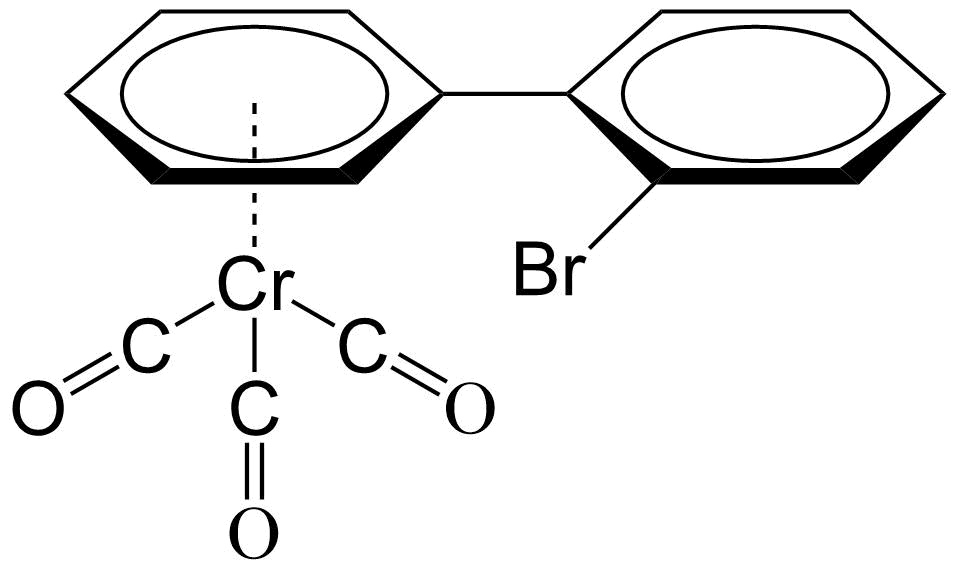

Supplement: Supplementary file 1 [file e-82-00107-sup2.zip › Berry_Guzei_Structures/Structure 4/Structure4.png]

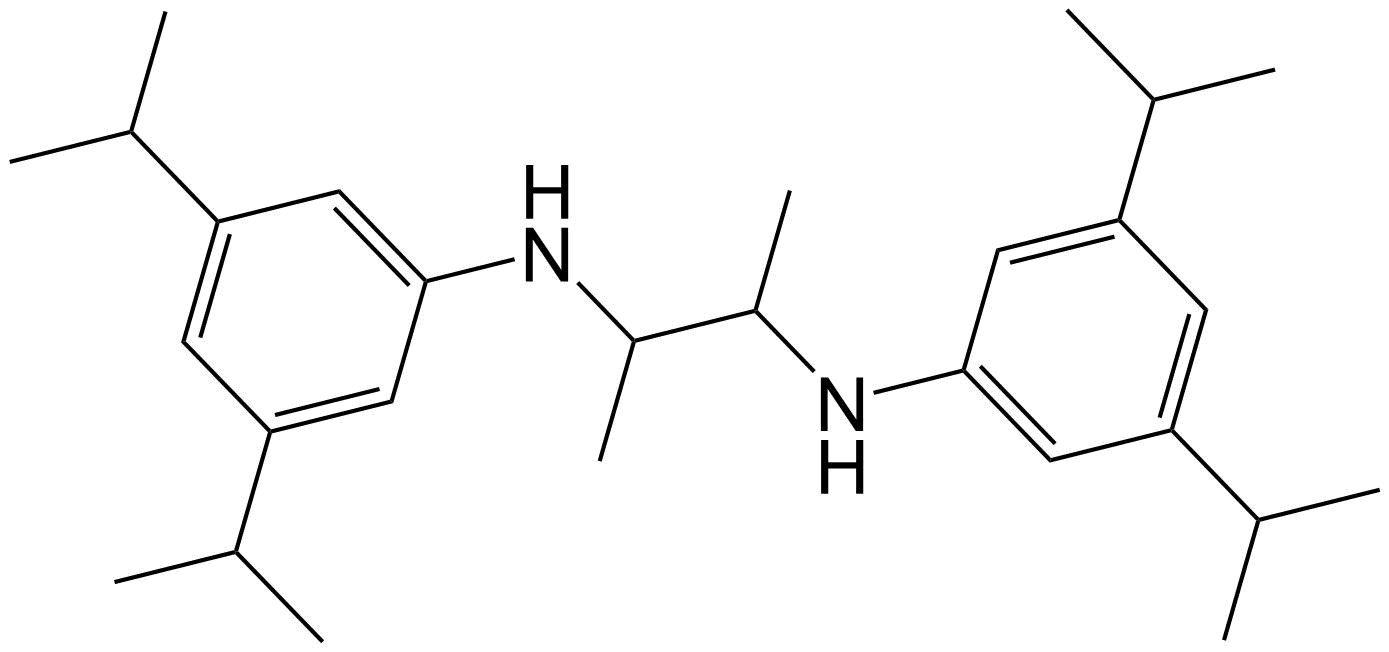

Supplement: Supplementary file 1 [file e-82-00107-sup2.zip › Berry_Guzei_Structures/Structure 5/Structure5.png]

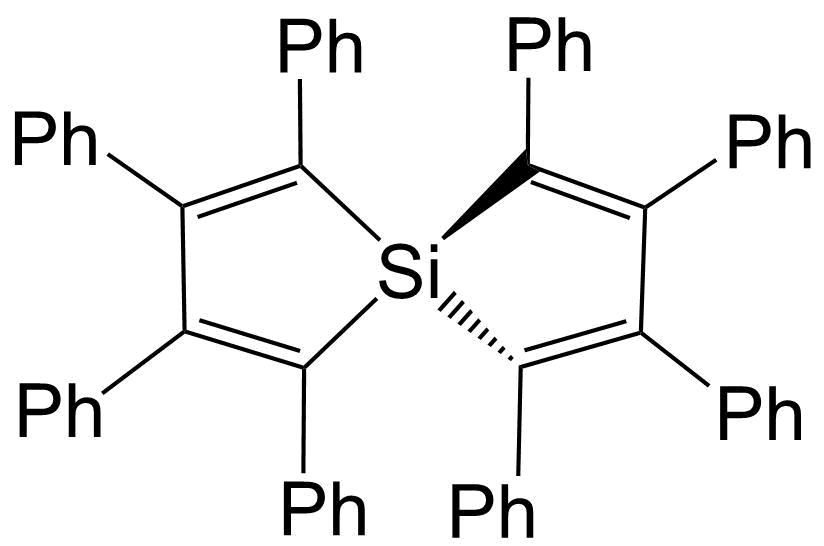

Supplement: Supplementary file 1 [file e-82-00107-sup2.zip › Berry_Guzei_Structures/Structure 6/structure6.png]

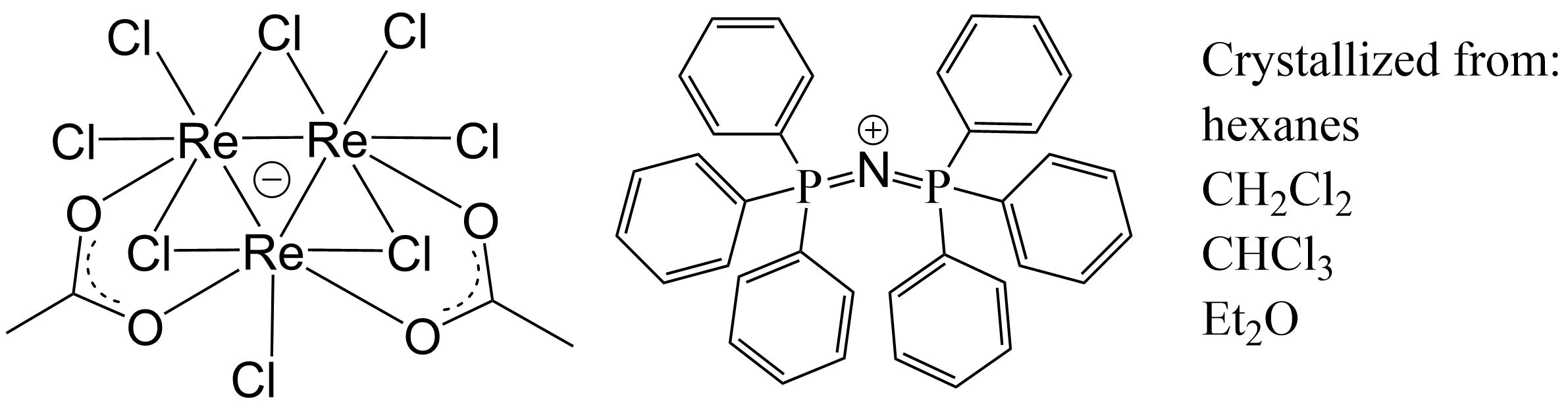

Supplement: Supplementary file 1 [file e-82-00107-sup2.zip › Berry_Guzei_Structures/Structure 7/Structure7.png]

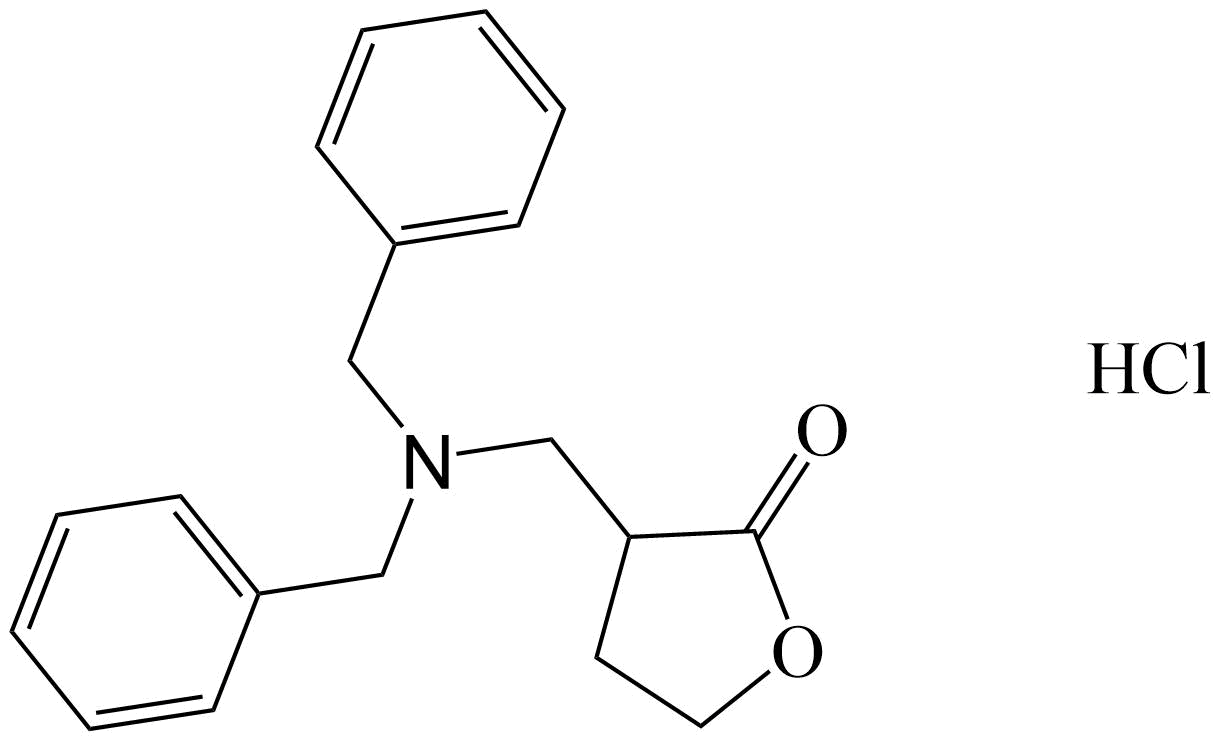

Supplement: Supplementary file 1 [file e-82-00107-sup2.zip › Berry_Guzei_Structures/Structure 8/structure8.png]

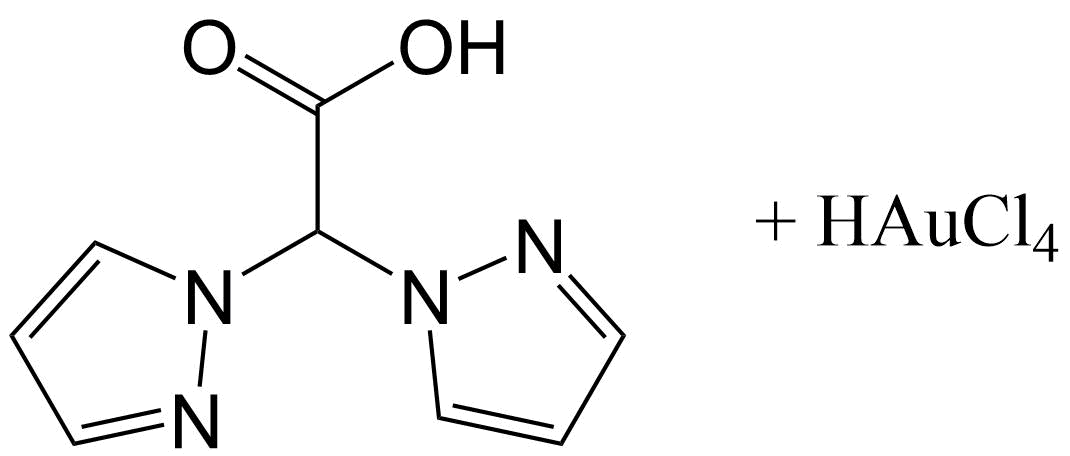

Supplement: Supplementary file 1 [file e-82-00107-sup2.zip › Berry_Guzei_Structures/Structure 9/Structure9.png]

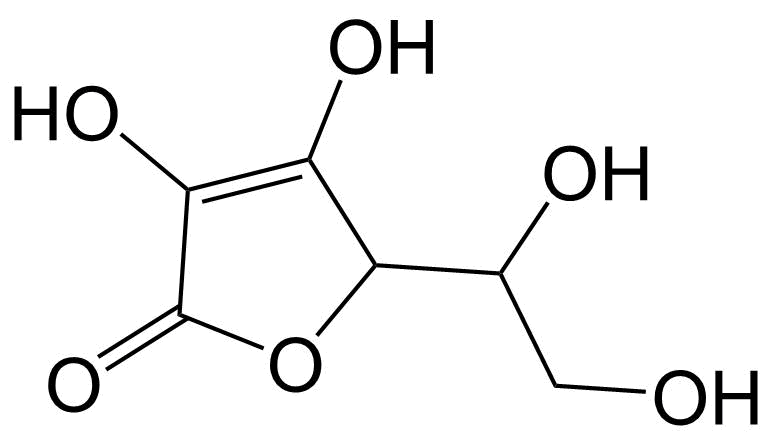

Supplement: Supplementary file 1 [file e-82-00107-sup2.zip › Berry_Guzei_Structures/Vitamin C/VitaminC.png]

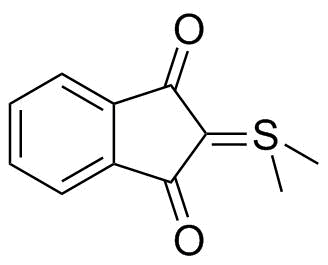

Supplement: Supplementary file 1 [file e-82-00107-sup2.zip › Berry_Guzei_Structures/Ylid twin/ylid.png]

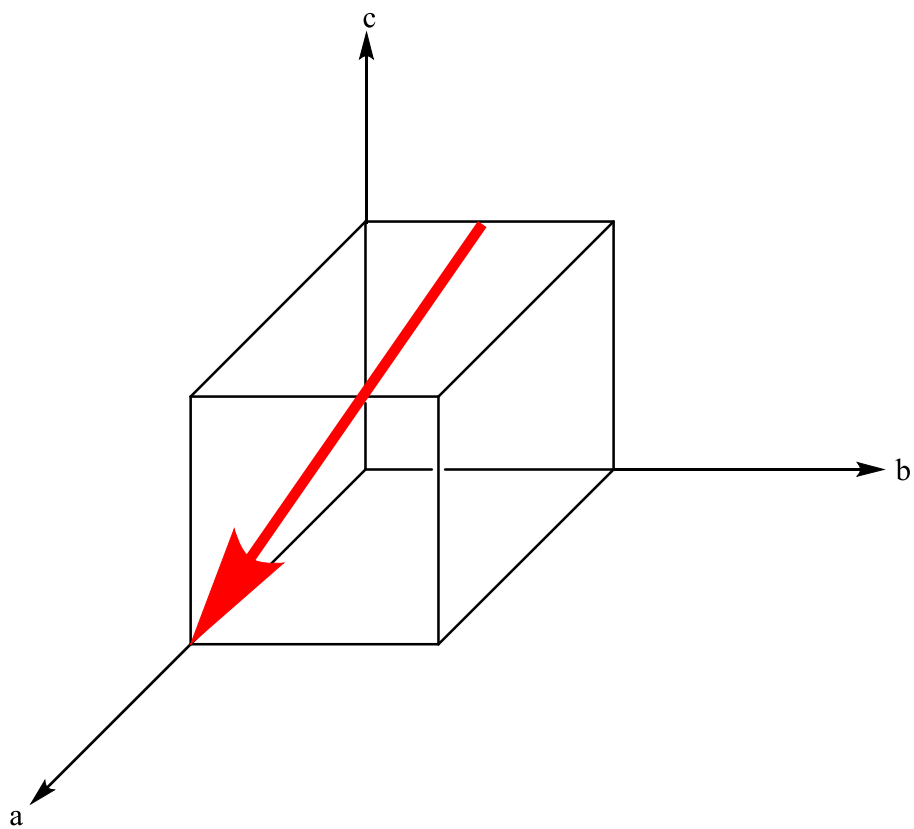

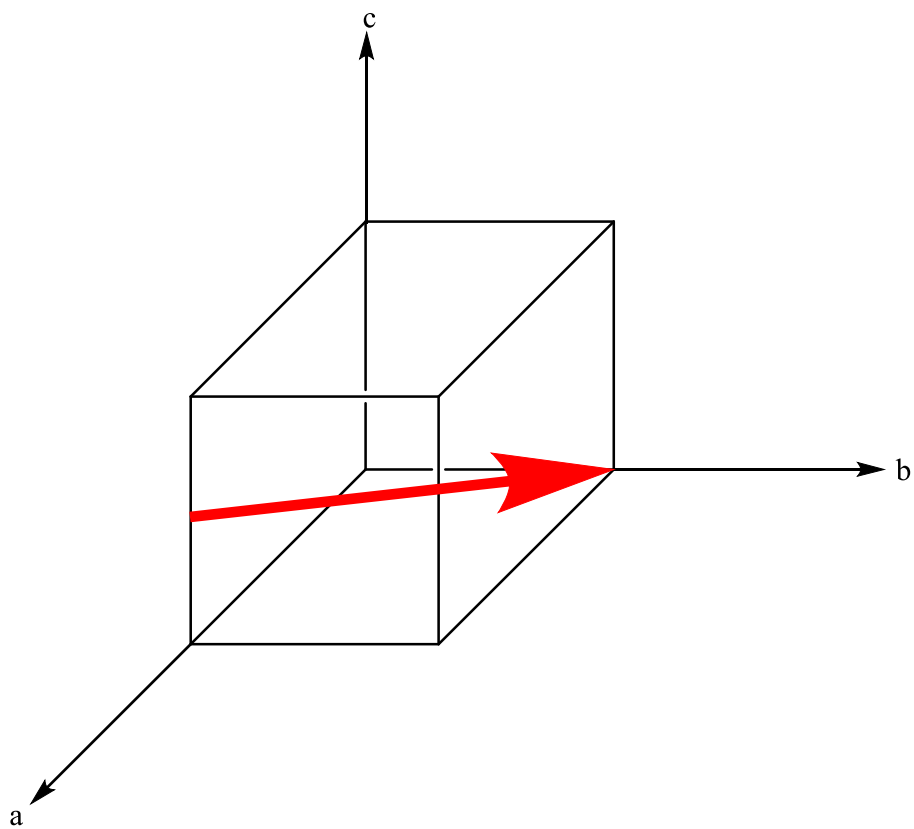

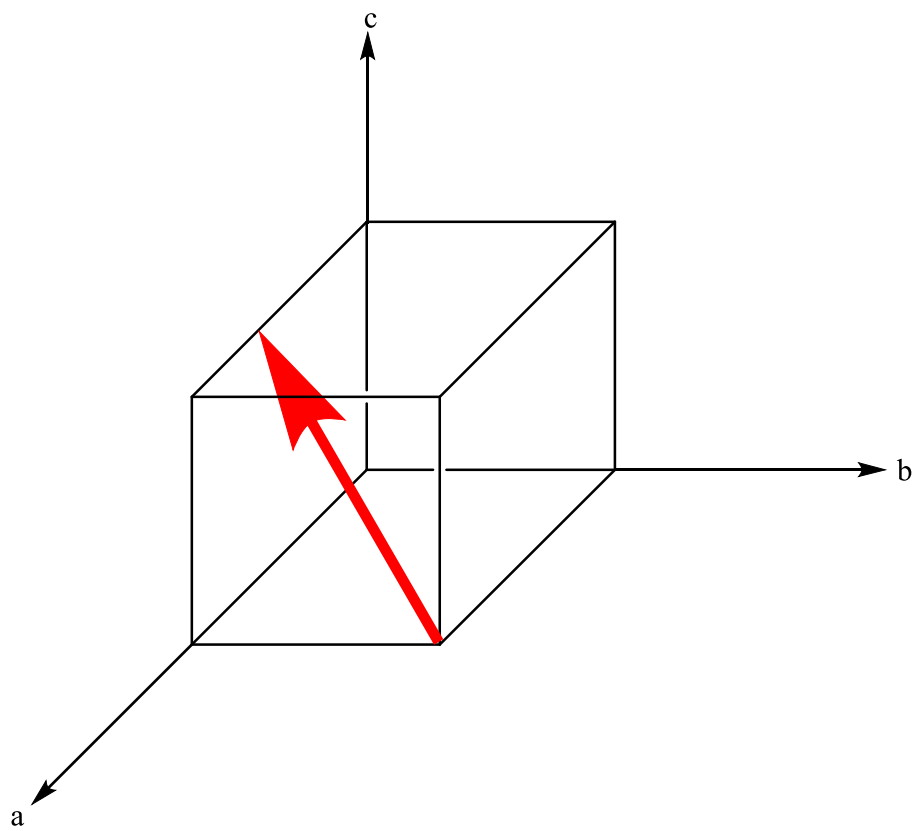

$[2\bar{1}1]$

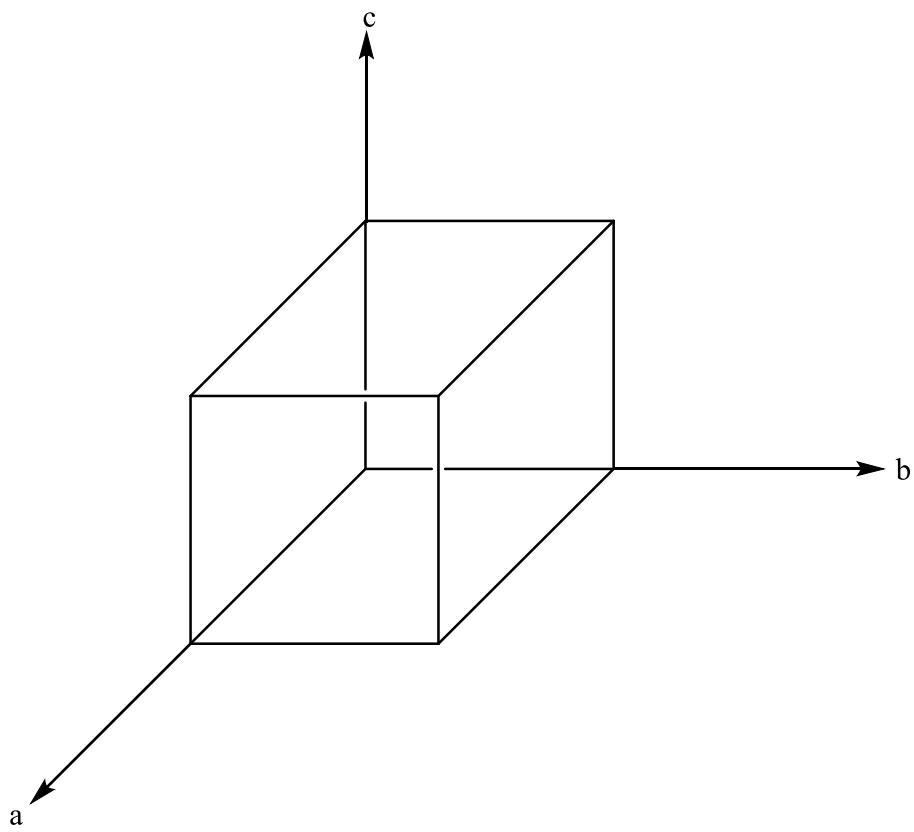

$[1\bar{1}\bar{2}]$

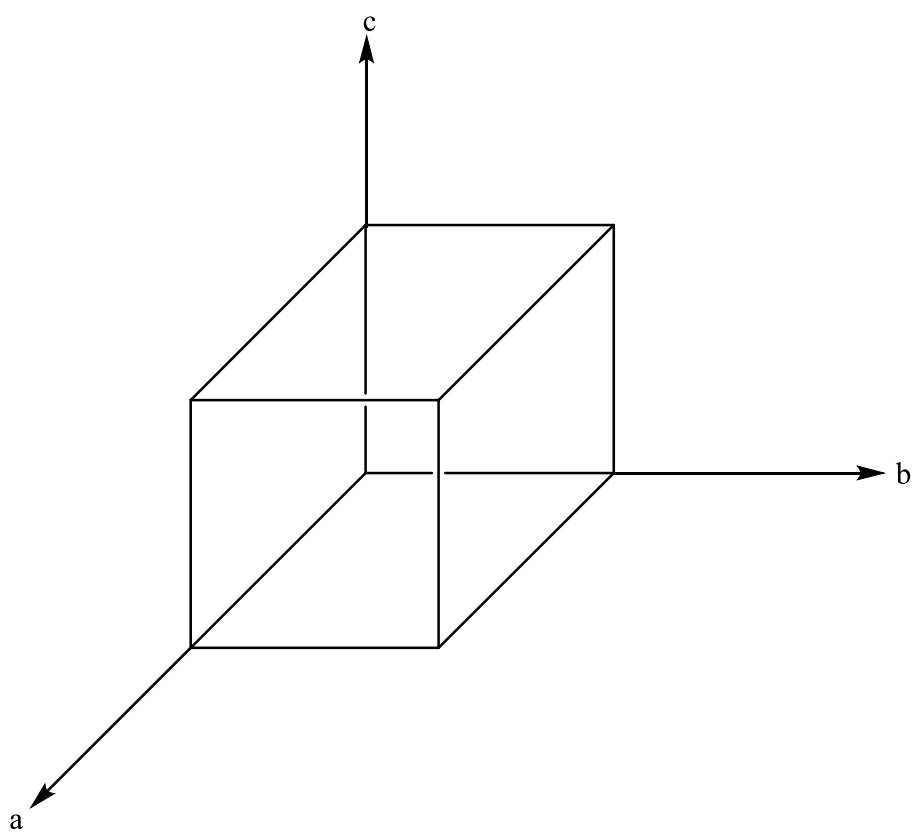

$[\bar{1}2\bar{2}]$

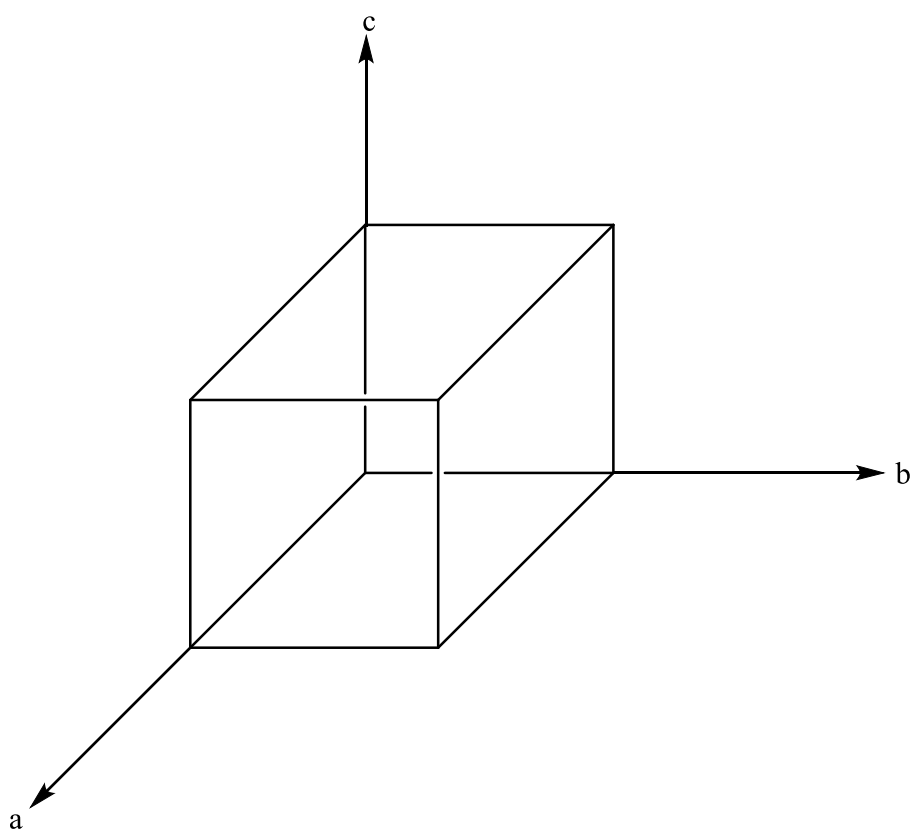

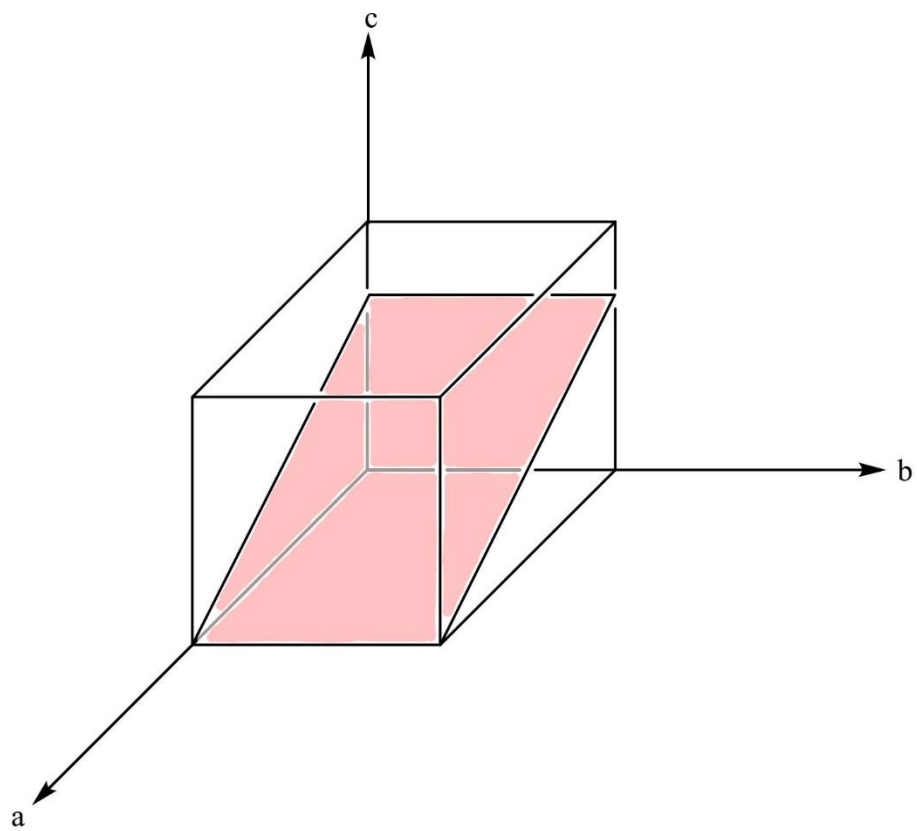

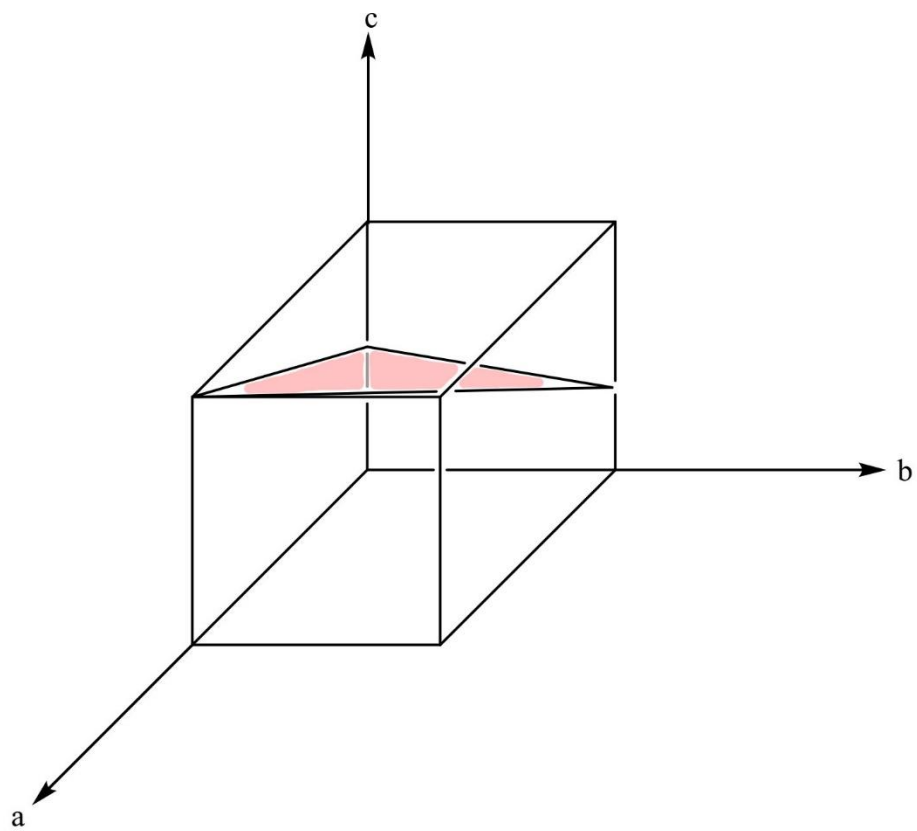

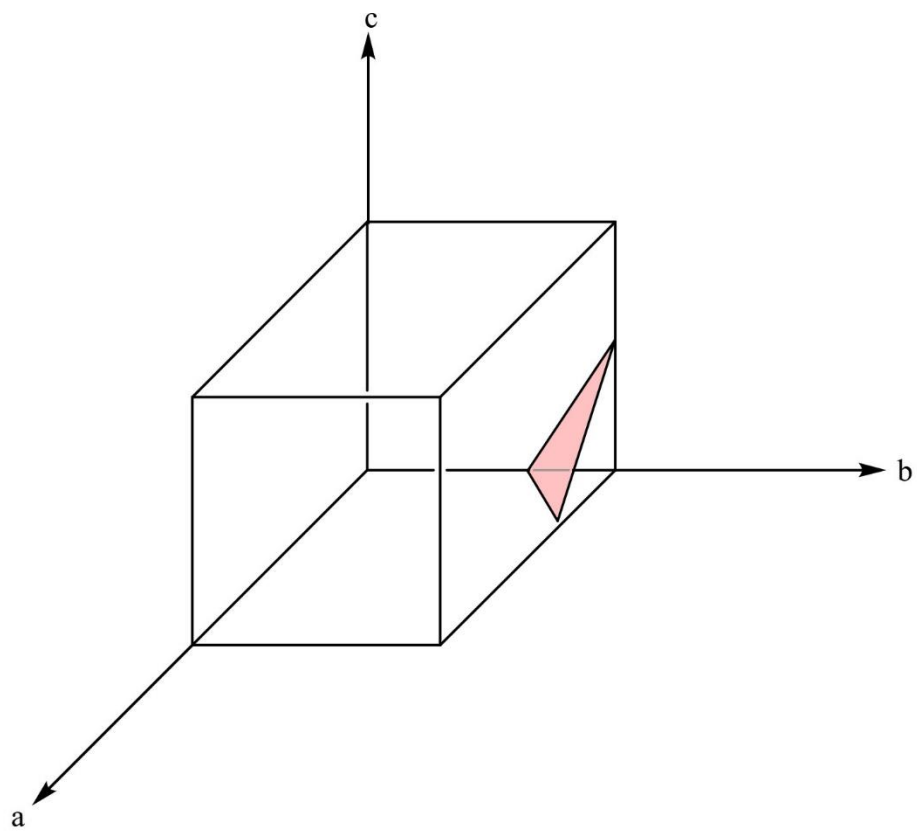

$(2\bar{1}1)$

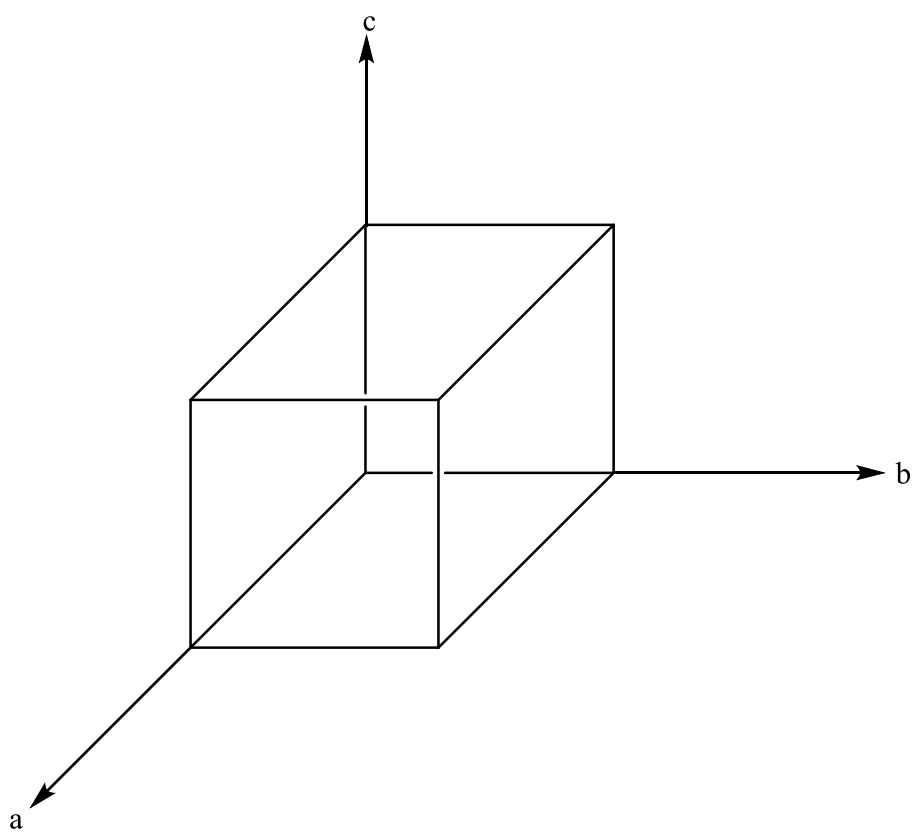

$(1\bar{1}\bar{2})$

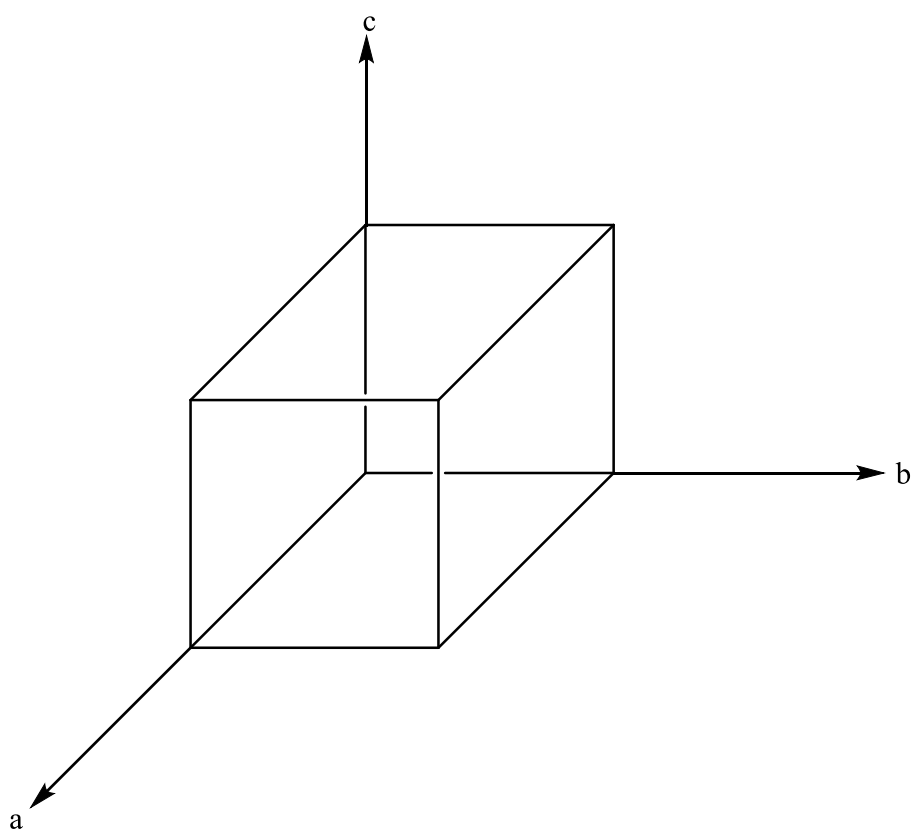

$(\bar{1}2\bar{2})$

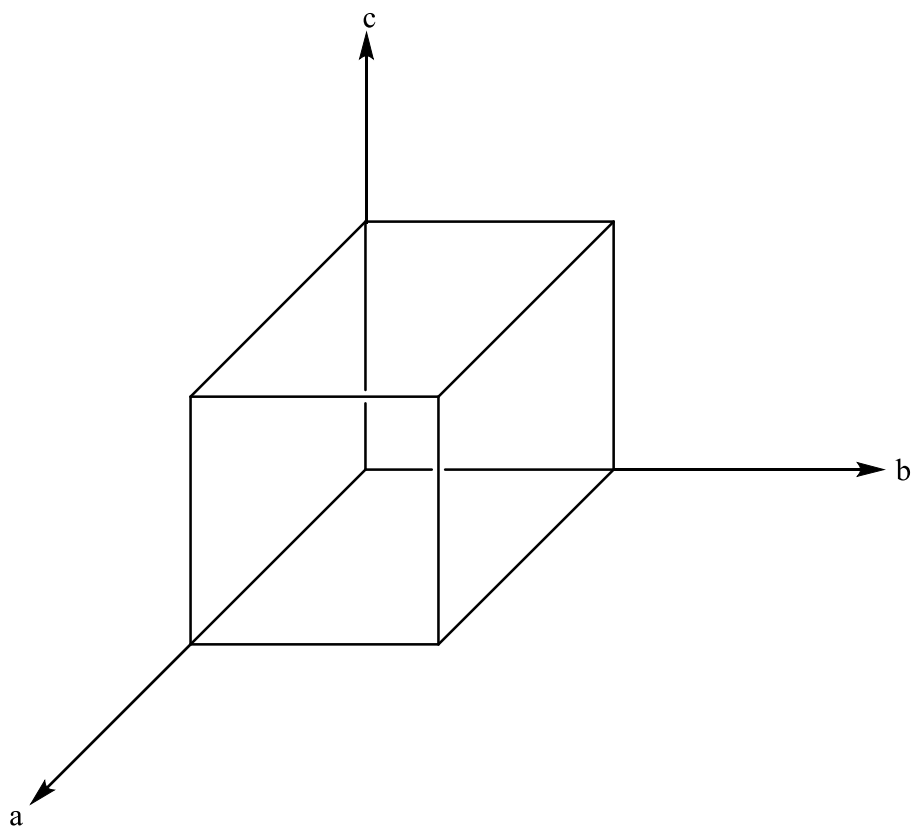

Supplement: Supplementary file 2 [file e-82-00107-sup3.zip › Directions & Planes Exercise.pdf]

### Symmetry Exercises 3

For each ribbon pattern, list all of the symmetry elements that you can find.

1.

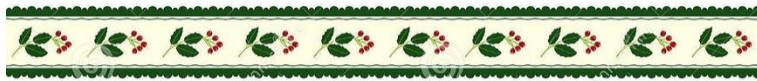

2.

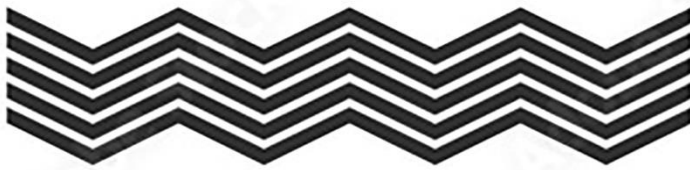

3.

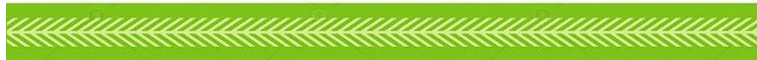

4.

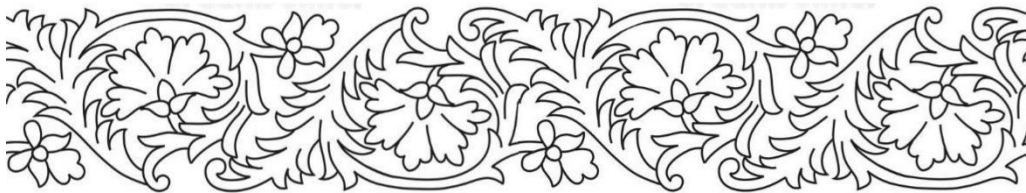

5.

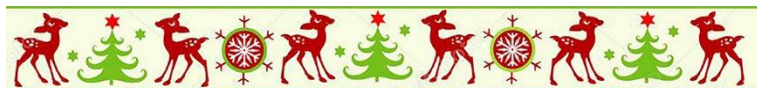

Supplement: Supplementary file 2 [file e-82-00107-sup3.zip › Symmetry Exercises 3.pdf]

## Symmetry Exercises 4

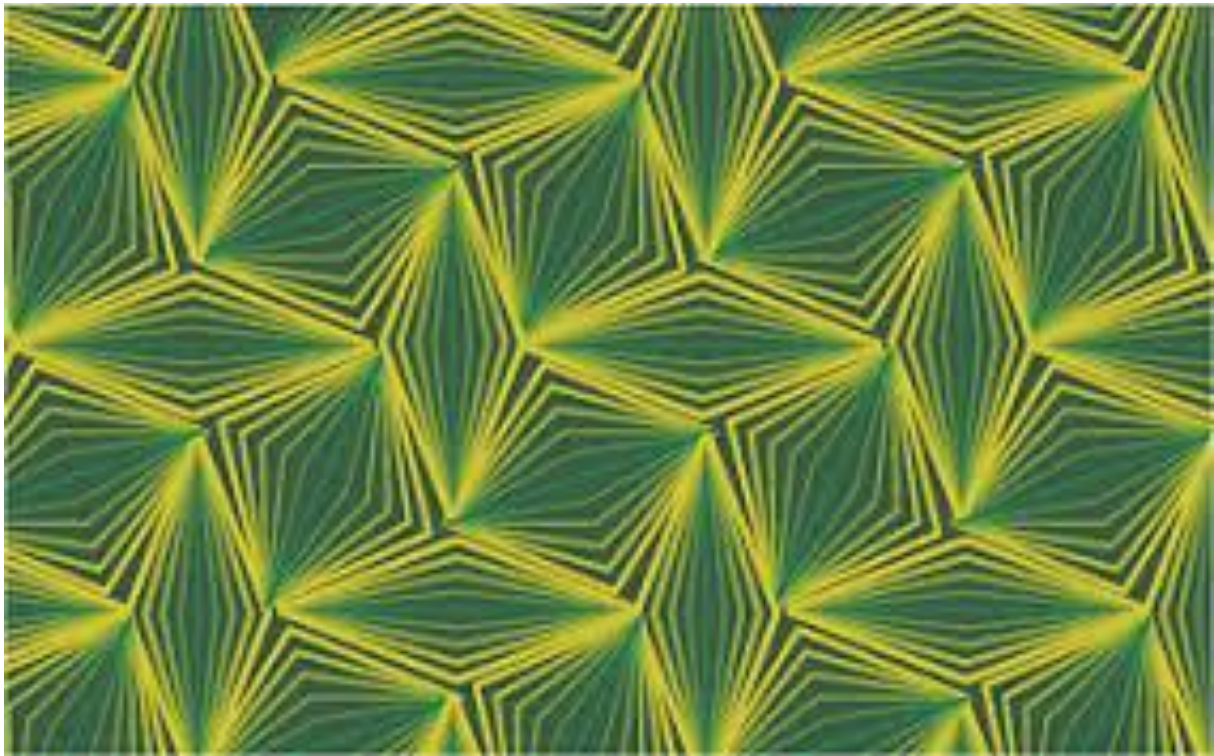

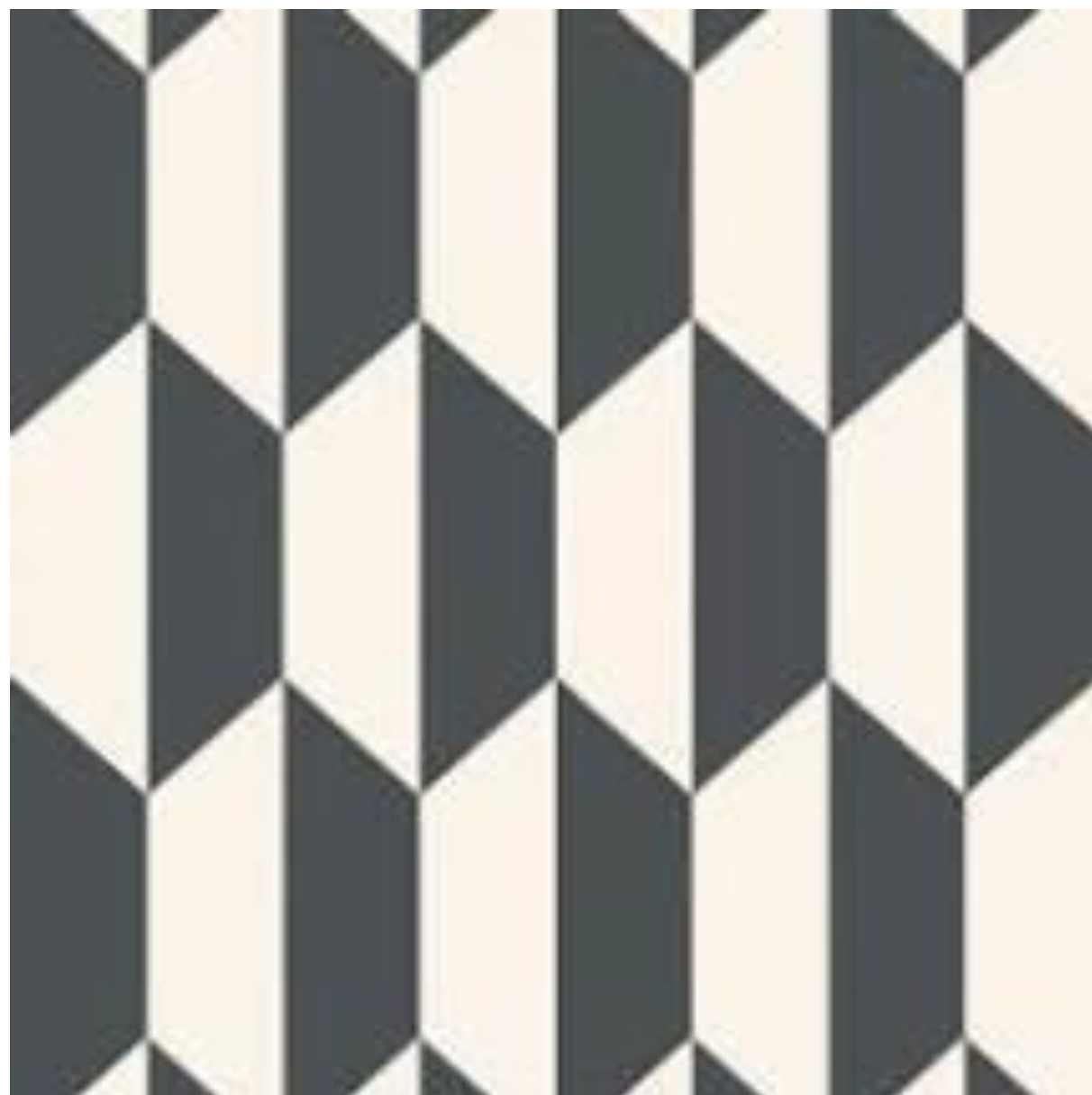

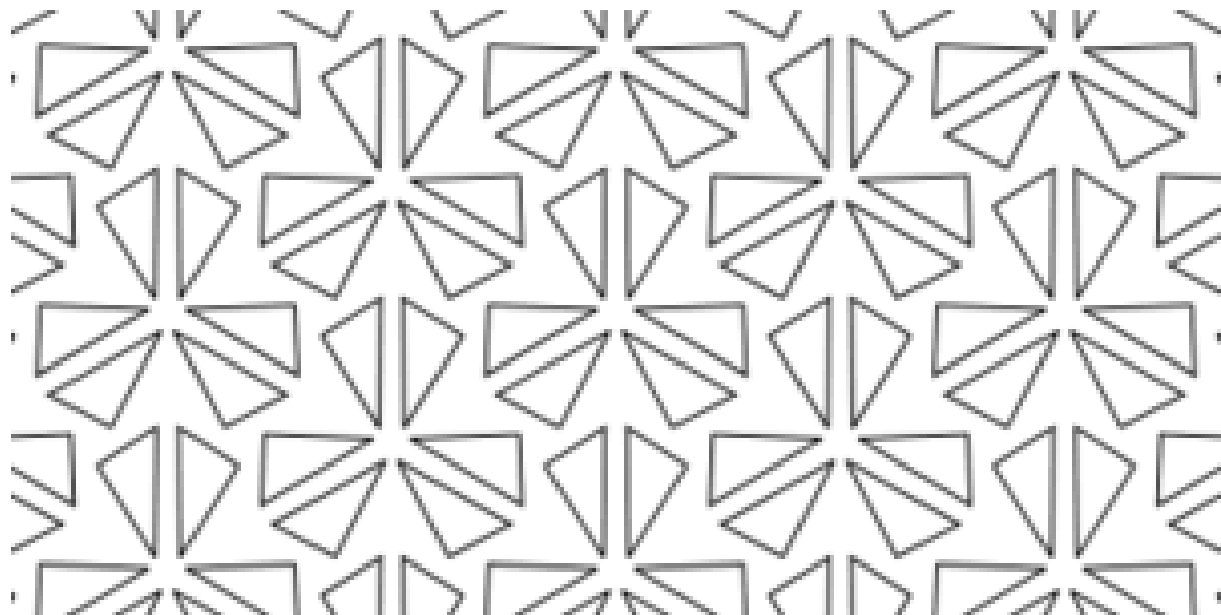

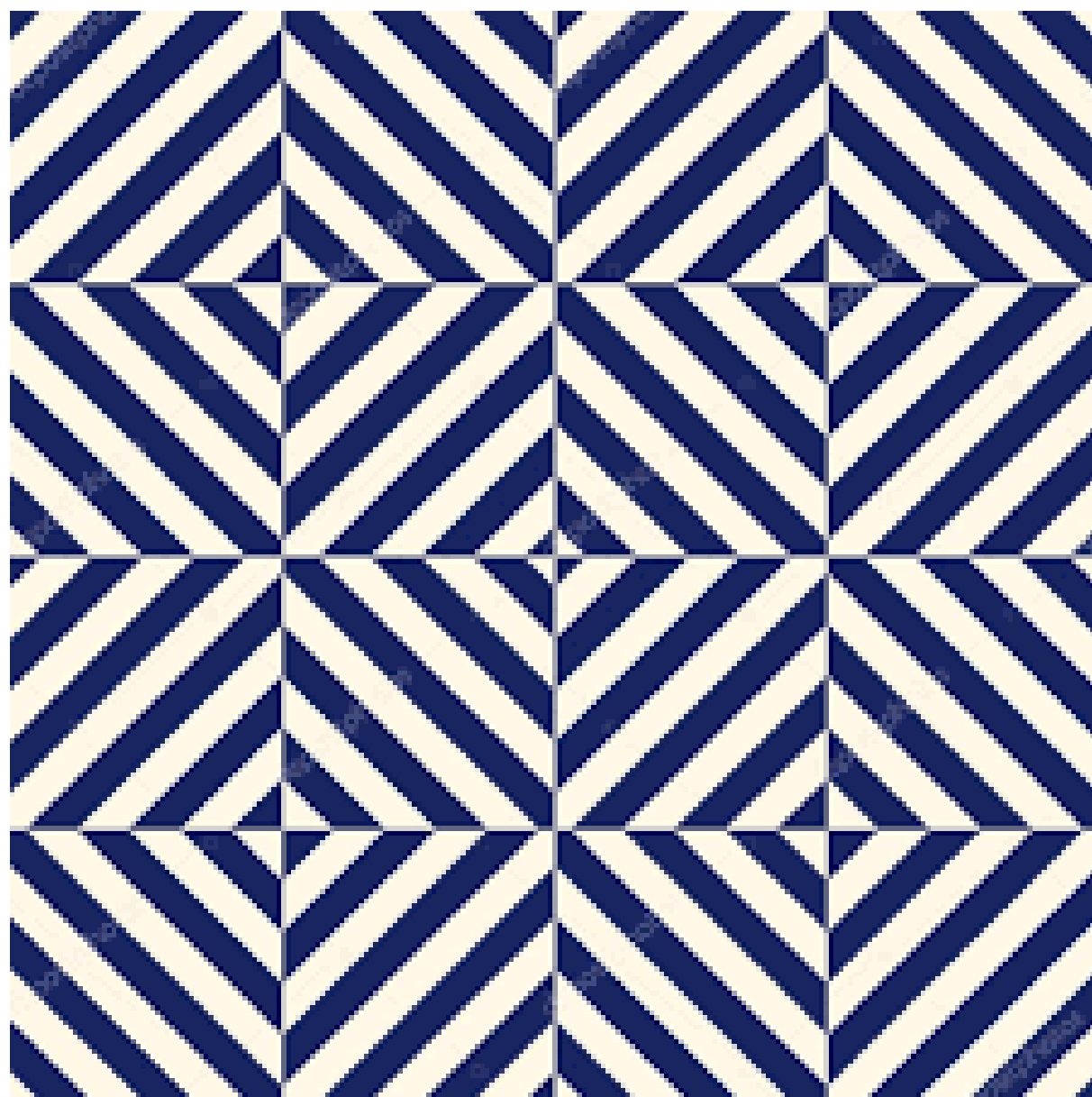

Supplement: Supplementary file 2 [file e-82-00107-sup3.zip › Symmetry Exercises 4.pdf]
