# Supplementary material for: Practical aspects of teaching a graduate-level small-mol­ecule chemical crystallography course
Source: Acta Crystallogr E Crystallogr Commun. 2026 Jan 1;82(Pt 1):107–20. doi: 10.1107/S2056989025010527 (PMC12810306; doi:10.1107/S2056989025010527)

CHEM 613

Vectors & Matrices

John F. Berry

1. Vectors – have magnitude & direction and represent a point in space.

$$\vec{u} = u \cdot \vec{a} + v \cdot \vec{b} + w \cdot \vec{c}$$

$$\vec{u} = \begin{pmatrix} u \\ v \\ w \end{pmatrix}$$

• Magnitude

$$|\vec{u}| = \sqrt{u^2 + v^2 + w^2}$$

• Addition

$$\vec{u}_1 + \vec{u}_2 \neq |\vec{u}_1| + |\vec{u}_2|$$

$$\vec{u}_1 + \vec{u}_2 = \begin{pmatrix} u_1 + u_2 \\ v_1 + v_2 \\ w_1 + w_2 \end{pmatrix}$$

• Multiplication

(a) Dot Product

$$\vec{u}_1 \cdot \vec{u}_2 = |\vec{u}_1| \cdot |\vec{u}_2| \cdot \cos\theta$$

Here, θ is the angle between $\vec{u}_1$ and $\vec{u}_2$.

In an orthonormal coordinate system (one in which the angles between $\vec{a}$, $\vec{b}$, and $\vec{c}$ are all 90°):

$$\vec{u}_1 \cdot \vec{u}_2 = u_1 \cdot u_2 + v_1 \cdot v_2 + w_1 \cdot w_2$$

In a general coordinate system:

$$\vec{u}_1 \cdot \vec{u}_2 = \vec{u}_1^{\mathrm{T}} \cdot \mathbf{M} \cdot \vec{u}_2$$

Here, $\vec{u}_1^{\mathrm{T}}$ is the *transpose* of $\vec{u}_1$:

if $\vec{u}_1 = \begin{pmatrix} u_1 \\ v_1 \\ w_1 \end{pmatrix}$, then $\vec{u}_1^{\mathrm{T}} = \begin{pmatrix} u_1 & v_1 & w_1 \end{pmatrix}$

and the *basis dot product matrix* **M** is:

$$\mathbf{M} = \begin{bmatrix} \vec{a} \cdot \vec{a} & \vec{a} \cdot \vec{b} & \vec{a} \cdot \vec{c} \\ \vec{b} \cdot \vec{a} & \vec{b} \cdot \vec{b} & \vec{b} \cdot \vec{c} \\ \vec{c} \cdot \vec{a} & \vec{c} \cdot \vec{b} & \vec{c} \cdot \vec{c} \end{bmatrix}$$

(b) Cross Product

$$\vec{u}_1 \times \vec{u}_2 = \vec{u}_3$$

The product is a vector. The magnitude of $\vec{u}_3$ is:

$$|\vec{u}_3| = |\vec{u}_1| \cdot |\vec{u}_2| \cdot \sin\theta$$

The direction of $\vec{u}_3$ is perpendicular to the $\{\vec{u}_1, \vec{u}_2\}$ plane, following the right-hand rule.

$$\vec{u}_1 \times \vec{u}_2 = \begin{vmatrix} \vec{a} & \vec{b} & \vec{c} \\ u_1 & v_1 & w_1 \\ u_2 & v_2 & w_2 \end{vmatrix} = (v_1 \cdot w_2 - w_1 \cdot v_2)\vec{a} + (w_1 \cdot u_2 - u_1 \cdot w_2)\vec{b} + (u_1 \cdot v_2 - v_1 \cdot u_2)\vec{c}$$

2. Matrices – We will mostly use 3 x 3 matrices

$$\mathbf{A} = \begin{pmatrix} a_{11} & a_{12} & a_{13} \\ a_{21} & a_{22} & a_{23} \\ a_{31} & a_{32} & a_{33} \end{pmatrix}; \mathbf{B} = \begin{pmatrix} b_{11} & b_{12} & b_{13} \\ b_{21} & b_{22} & b_{23} \\ b_{31} & b_{32} & b_{33} \end{pmatrix}; \mathbf{C} = \begin{pmatrix} c_{11} & c_{12} \\ c_{21} & c_{22} \end{pmatrix}$$

• Addition

$$\mathbf{A} + \mathbf{B} = \begin{pmatrix} a_{11} + b_{11} & a_{12} + b_{12} & a_{13} + b_{13} \\ a_{21} + b_{21} & a_{22} + b_{22} & a_{23} + b_{23} \\ a_{31} + b_{31} & a_{32} + b_{32} & a_{33} + b_{33} \end{pmatrix}$$

• Multiplication

$$\mathbf{AB} = \begin{pmatrix} a_{11}b_{11} + a_{12}b_{21} + a_{13}b_{31} & a_{11}b_{12} + a_{12}b_{22} + a_{13}b_{32} & a_{11}b_{13} + a_{12}b_{23} + a_{13}b_{33} \\ a_{21}b_{11} + a_{22}b_{21} + a_{23}b_{31} & a_{21}b_{12} + a_{22}b_{22} + a_{23}b_{32} & a_{21}b_{13} + a_{22}b_{23} + a_{23}b_{33} \\ a_{31}b_{11} + a_{32}b_{21} + a_{33}b_{31} & a_{31}b_{12} + a_{32}b_{22} + a_{33}b_{32} & a_{31}b_{13} + a_{32}b_{23} + a_{33}b_{33} \end{pmatrix}$$

$$\mathbf{A}\vec{u} = \begin{pmatrix} a_{11} & a_{12} & a_{13} \\ a_{21} & a_{22} & a_{23} \\ a_{31} & a_{32} & a_{33} \end{pmatrix} \begin{pmatrix} u \\ v \\ w \end{pmatrix} = \begin{pmatrix} a_{11}u + a_{12}v + a_{13}w \\ a_{21}u + a_{22}v + a_{23}w \\ a_{31}u + a_{32}v + a_{33}w \end{pmatrix}$$

• Determinants

2 x 2:

$$\det\mathbf{C} = \begin{vmatrix} c_{11} & c_{12} \\ c_{21} & c_{22} \end{vmatrix} = c_{11}c_{22} - c_{12}c_{21}$$

3 x 3:

$$\det\mathbf{A} = \begin{vmatrix} a_{11} & a_{12} & a_{13} \\ a_{21} & a_{22} & a_{23} \\ a_{31} & a_{32} & a_{33} \end{vmatrix} = a_{11}\begin{vmatrix} a_{22} & a_{23} \\ a_{32} & a_{33} \end{vmatrix} - a_{12}\begin{vmatrix} a_{21} & a_{23} \\ a_{31} & a_{33} \end{vmatrix} + a_{13}\begin{vmatrix} a_{21} & a_{22} \\ a_{31} & a_{32} \end{vmatrix}$$

$$= a_{11}a_{22}a_{33} + a_{12}a_{23}a_{31} + a_{13}a_{21}a_{32} - a_{13}a_{22}a_{31} - a_{11}a_{23}a_{32} - a_{12}a_{21}a_{33}$$

3. Matrices for symmetry operations

• Proper Rotations

$$1 \equiv \begin{pmatrix} 1 & 0 & 0 \\ 0 & 1 & 0 \\ 0 & 0 & 1 \end{pmatrix}; 2_a \equiv \begin{pmatrix} 1 & 0 & 0 \\ 0 & \bar{1} & 0 \\ 0 & 0 & \bar{1} \end{pmatrix}; 2_b \equiv \begin{pmatrix} \bar{1} & 0 & 0 \\ 0 & 1 & 0 \\ 0 & 0 & \bar{1} \end{pmatrix}; 2_c \equiv \begin{pmatrix} \bar{1} & 0 & 0 \\ 0 & \bar{1} & 0 \\ 0 & 0 & 1 \end{pmatrix};$$

$$3 \equiv \begin{pmatrix} 0 & \bar{1} & 0 \\ 1 & \bar{1} & 0 \\ 0 & 0 & 1 \end{pmatrix}; 4 \equiv \begin{pmatrix} 0 & \bar{1} & 0 \\ 1 & 0 & 0 \\ 0 & 0 & 1 \end{pmatrix}; 6 \equiv \begin{pmatrix} 1 & \bar{1} & 0 \\ 1 & 0 & 0 \\ 0 & 0 & 1 \end{pmatrix}$$

To use these transformations, we can multiply them by a fractional coordinate vector $\begin{pmatrix} x \\ y \\ z \end{pmatrix}$ to get the new fractional coordinates $\begin{pmatrix} \tilde{x} \\ \tilde{y} \\ \tilde{z} \end{pmatrix}$.

For example, the three-fold rotation acts as follows:

$$\begin{pmatrix} \tilde{x} \\ \tilde{y} \\ \tilde{z} \end{pmatrix} = \begin{pmatrix} 0 & \bar{1} & 0 \\ 1 & \bar{1} & 0 \\ 0 & 0 & 1 \end{pmatrix}\begin{pmatrix} x \\ y \\ z \end{pmatrix} = \begin{pmatrix} \bar{y} \\ x - y \\ z \end{pmatrix}$$

Note that three-fold and six-fold rotations use the trigonal/hexagonal lattice in which γ = 120°.

• Improper Rotations

$$\bar{1} \equiv \begin{pmatrix} \bar{1} & 0 & 0 \\ 0 & \bar{1} & 0 \\ 0 & 0 & \bar{1} \end{pmatrix}; m_{ab} \equiv \begin{pmatrix} 1 & 0 & 0 \\ 0 & 1 & 0 \\ 0 & 0 & \bar{1} \end{pmatrix}; m_{ac} \equiv \begin{pmatrix} 1 & 0 & 0 \\ 0 & \bar{1} & 0 \\ 0 & 0 & 1 \end{pmatrix}; m_{bc} \equiv \begin{pmatrix} \bar{1} & 0 & 0 \\ 0 & 1 & 0 \\ 0 & 0 & 1 \end{pmatrix};$$

$$\bar{3} \equiv \begin{pmatrix} 0 & 1 & 0 \\ \bar{1} & 1 & 0 \\ 0 & 0 & \bar{1} \end{pmatrix}; \bar{4} \equiv \begin{pmatrix} 0 & 1 & 0 \\ \bar{1} & 0 & 0 \\ 0 & 0 & \bar{1} \end{pmatrix}; \bar{6} \equiv \begin{pmatrix} \bar{1} & 1 & 0 \\ \bar{1} & 0 & 0 \\ 0 & 0 & \bar{1} \end{pmatrix}$$

• Travel Symmetry Elements – Consist of a symmetry transform **T**, plus a translation, $\vec{t}$:

$$\begin{pmatrix} \tilde{x} \\ \tilde{y} \\ \tilde{z} \end{pmatrix} = \begin{pmatrix} T_{11} & T_{12} & T_{13} \\ T_{21} & T_{22} & T_{23} \\ T_{31} & T_{32} & T_{33} \end{pmatrix}\begin{pmatrix} x \\ y \\ z \end{pmatrix} + \begin{pmatrix} t_1 \\ t_2 \\ t_3 \end{pmatrix}$$

For example, a 2_1 axis along $\vec{b}$:

$$\mathbf{T} = \begin{pmatrix} \bar{1} & 0 & 0 \\ 0 & 1 & 0 \\ 0 & 0 & \bar{1} \end{pmatrix}; \vec{t} = \begin{pmatrix} 0 \\ 1/2 \\ 0 \end{pmatrix}$$

Under this operation, $\begin{pmatrix} x \\ y \\ z \end{pmatrix}$ transforms to $\begin{pmatrix} \bar{x} \\ y + \frac{1}{2} \\ \bar{z} \end{pmatrix}$.

4. Augmented Matrices for Travel Symmetry Operations

Another way to represent travel symmetry with components **T** and $\vec{t}$ is through the use of 4 x 4 augmented matrices as illustrated below:

$$(\mathbf{T}, \vec{t}) = \mathbb{W} = \left(\begin{array}{ccc|c} & \mathbf{T} & & \vec{t} \\ \hline 0 & 0 & 0 & 1 \end{array}\right)$$

The augmented matrices operate on augmented vectors of the type:

$$\vec{\mathbb{u}} = \begin{pmatrix} x \\ y \\ z \\ \hline 1 \end{pmatrix}$$

Such that $\vec{\mathbb{u}}' = \mathbb{W} \cdot \vec{\mathbb{u}}$.

For example, suppose we wish to find the location of the inversion center that is created by the combination of a 2_1 axis along $\vec{b}$ and a *c* glide plane. We can find the new point $\vec{\mathbb{u}}'$ that is generated starting from the origin ($\vec{\mathbb{u}} = \begin{pmatrix} 0 \\ 0 \\ 0 \\ \hline 1 \end{pmatrix}$) by a combination of the two symmetry elements. The point $\vec{\mathbb{u}}'$ can be determined by:

$$\vec{\mathbb{u}}' = (2_1(\vec{b})) \cdot (c) \cdot \vec{\mathbb{u}}$$

And in full augmented matrix form:

$$\vec{u}' = \left(\begin{array}{ccc|c} \bar{1} & 0 & 0 & 0 \\ 0 & 1 & 0 & 1/2 \\ 0 & 0 & \bar{1} & 0 \\ \hline 0 & 0 & 0 & 1 \end{array}\right) \cdot \left(\begin{array}{ccc|c} 1 & 0 & 0 & 0 \\ 0 & \bar{1} & 0 & 0 \\ 0 & 0 & 1 & 1/2 \\ \hline 0 & 0 & 0 & 1 \end{array}\right) \cdot \left(\begin{array}{c} 0 \\ 0 \\ 0 \\ \hline 1 \end{array}\right)$$

$$= \left(\begin{array}{ccc|c} \bar{1} & 0 & 0 & 0 \\ 0 & \bar{1} & 0 & 1/2 \\ 0 & 0 & \bar{1} & 1/2 \\ \hline 0 & 0 & 0 & 1 \end{array}\right) \cdot \left(\begin{array}{c} 0 \\ 0 \\ 0 \\ \hline 1 \end{array}\right) = \left(\begin{array}{c} 0 \\ 1/2 \\ 1/2 \\ \hline 1 \end{array}\right)$$

Thus, the inversion center transforms $\vec{u} = \begin{pmatrix} 0 \\ 0 \\ 0 \end{pmatrix}$ to $\vec{u}' = \begin{pmatrix} 0 \\ 1/2 \\ 1/2 \end{pmatrix}$. The inversion center is therefore located at $\begin{pmatrix} 0 \\ 1/4 \\ 1/4 \end{pmatrix}$.

In crystallography, centrosymmetric space groups use the inversion center as the origin by convention. Thus, in the space group $P2_1/c$, rather than having the intersection of the 2_1 and *c* glides be at the origin, the origin is shifted by $\begin{pmatrix} 0 \\ 1/4 \\ 1/4 \end{pmatrix}$ so that it contains the inversion center. Thus, in the diagram below, note that the 2_1 axes are at ¼ in the $\vec{c}$ direction, and the *c* glide is at ¼ in the $\vec{b}$ direction.

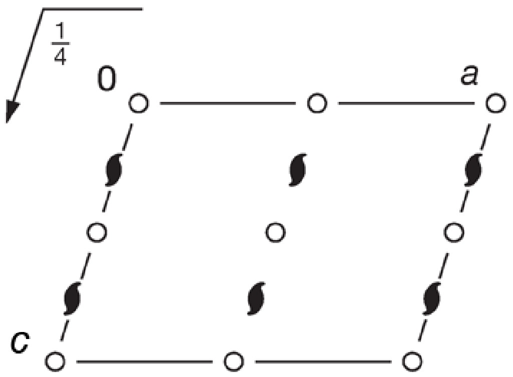

Supplement: Supplementary file 2 [file e-82-00107-sup3.zip › Vectors and Matrices.pdf]
