# Supplementary material for: Practical aspects of teaching a graduate-level small-mol­ecule chemical crystallography course
Source: Acta Crystallogr E Crystallogr Commun. 2026 Jan 1;82(Pt 1):107–20. doi: 10.1107/S2056989025010527 (PMC12810306; doi:10.1107/S2056989025010527)
Supplement: Supplementary file 2 [file e-82-00107-sup3.zip › Complex Numbers.pdf]

CHEM 613

Complex Numbers

John F. Berry

Complex numbers contain $i = \sqrt{-1}$. They have three major forms, as shown in the Argand diagram, Figure 1: a vector form, a polar form, and an exponential form.

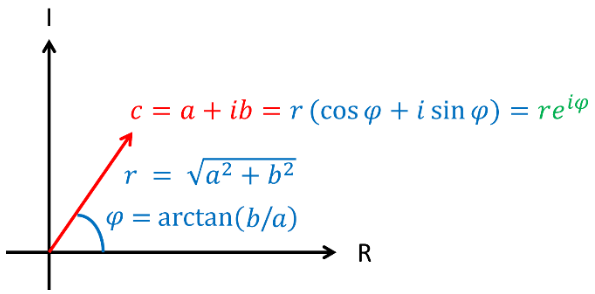

• Forms of complex numbers:

– Vector form: $c = a + ib$
– Polar form: $c = r(\cos\varphi + i\sin\varphi)$
– Exponential form: $c = re^{i\varphi}$

• Addition and subtraction:

– This is easiest in vector form:

$$c_1 + c_2 = a_1 + a_2 + i(b_1 + b_2)$$

$$c_1 - c_2 = a_1 - a_2 + i(b_1 - b_2)$$

• Multiplication:

– Vector form:

$$c_1 \cdot c_2 = a_1 \cdot a_2 - b_1 \cdot b_2 + i(a_1 \cdot b_2 + b_1 \cdot a_2)$$

– Polar form:

$$c_1 \cdot c_2 = r_1 \cdot r_2(\cos(\varphi_1 + \varphi_2) + i\sin(\varphi_1 + \varphi_2))$$

– Exponential form:

$$c_1 \cdot c_2 = r_1 \cdot r_2 \cdot e^{i(\varphi_1 + \varphi_2)}$$

• Division:

– Vector form:

$$\frac{c_1}{c_2} = \frac{a_1 \cdot a_2 + b_1 \cdot b_2}{a_2^2 + b_2^2} + i\frac{a_2 \cdot b_1 - a_1 \cdot b_2}{a_2^2 + b_2^2}$$

– Polar form:

$$\frac{c_1}{c_2} = \frac{r_1}{r_2}(\cos(\varphi_1 - \varphi_2) + i\sin(\varphi_1 - \varphi_2))$$

– Exponential form:

$$\frac{c_1}{c_2} = \frac{r_1}{r_2}e^{i(\varphi_1 - \varphi_2)}$$

- Complex Conjugates:
  - Vector form:

$$c^* = a - ib$$

  - Polar form:

$$c^* = r(\cos\varphi - i\sin\varphi)$$

  - Exponential form:

$$c^* = r \cdot e^{-i\cdot\varphi}$$

- Modulus (length of the vector):

$$|c| = \sqrt{a^2 + b^2}$$

$$|c|^2 = c \cdot c^* = a^2 + b^2$$

$$\left|e^{i\cdot\varphi}\right| = 1$$

$$\left|r \cdot e^{i\cdot\varphi}\right| = r$$

- Conversion from Cartesian to polar coordinates:

$$r = \sqrt{a^2 + b^2};\ \tan\varphi = \frac{b}{a}$$

- Useful Trigonometric relationships:

$$\cos(-\varphi) = \cos\varphi$$

$$\sin(-\varphi) = -\sin\varphi$$
