# Supplementary material for: Practical aspects of teaching a graduate-level small-mol­ecule chemical crystallography course
Source: Acta Crystallogr E Crystallogr Commun. 2026 Jan 1;82(Pt 1):107–20. doi: 10.1107/S2056989025010527 (PMC12810306; doi:10.1107/S2056989025010527)

Data Analysis Exercise

Identify the point symmetry of the diffraction pattern and any reflection conditions giving rise to systematic absences, and use this information to determine the likely space groups of the crystal. The width of each image is 2.00 $Å^{-1}$.

0kl Zone:

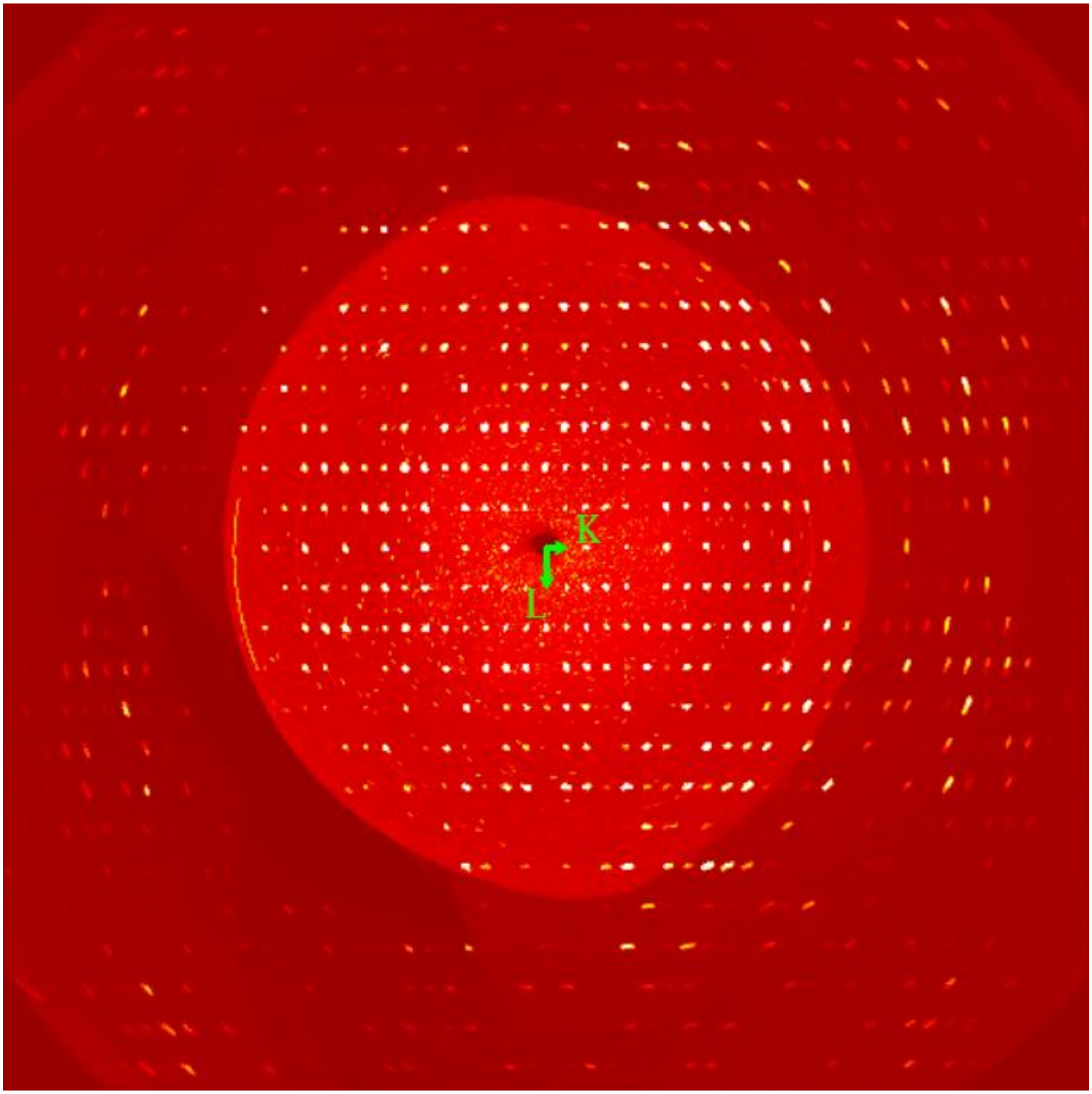

1kl Zone

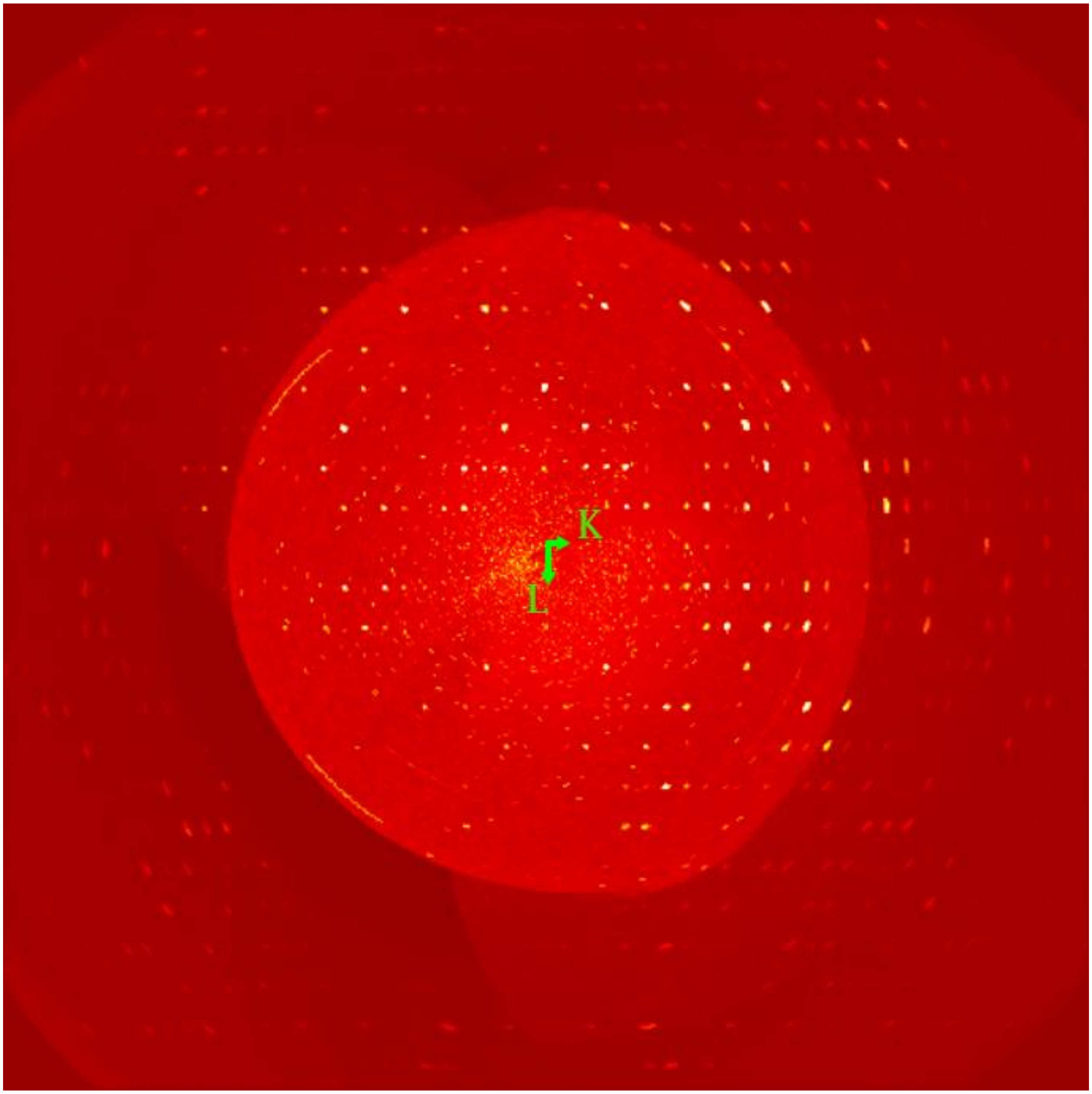

h0l Zone

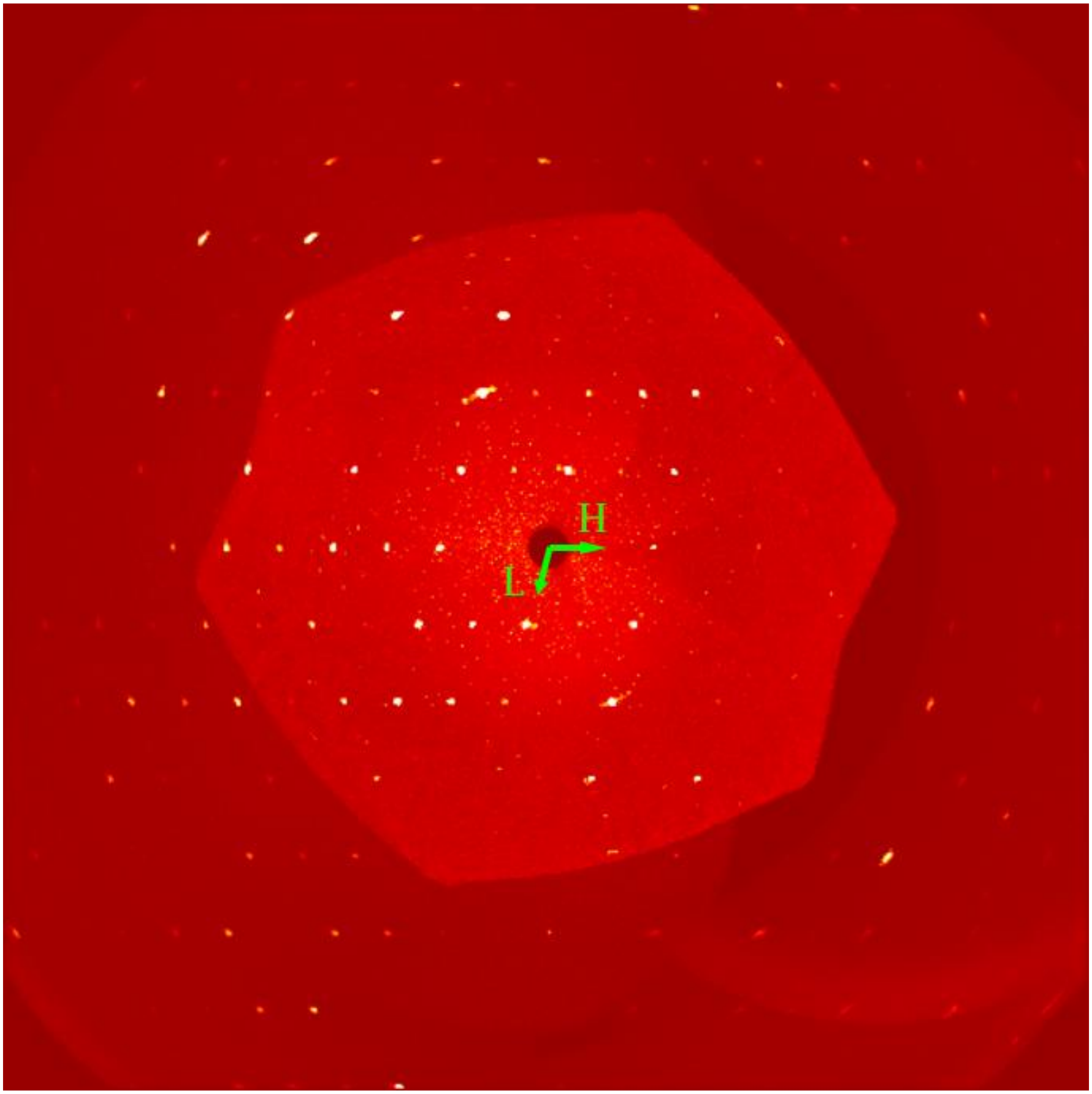

h1l Zone

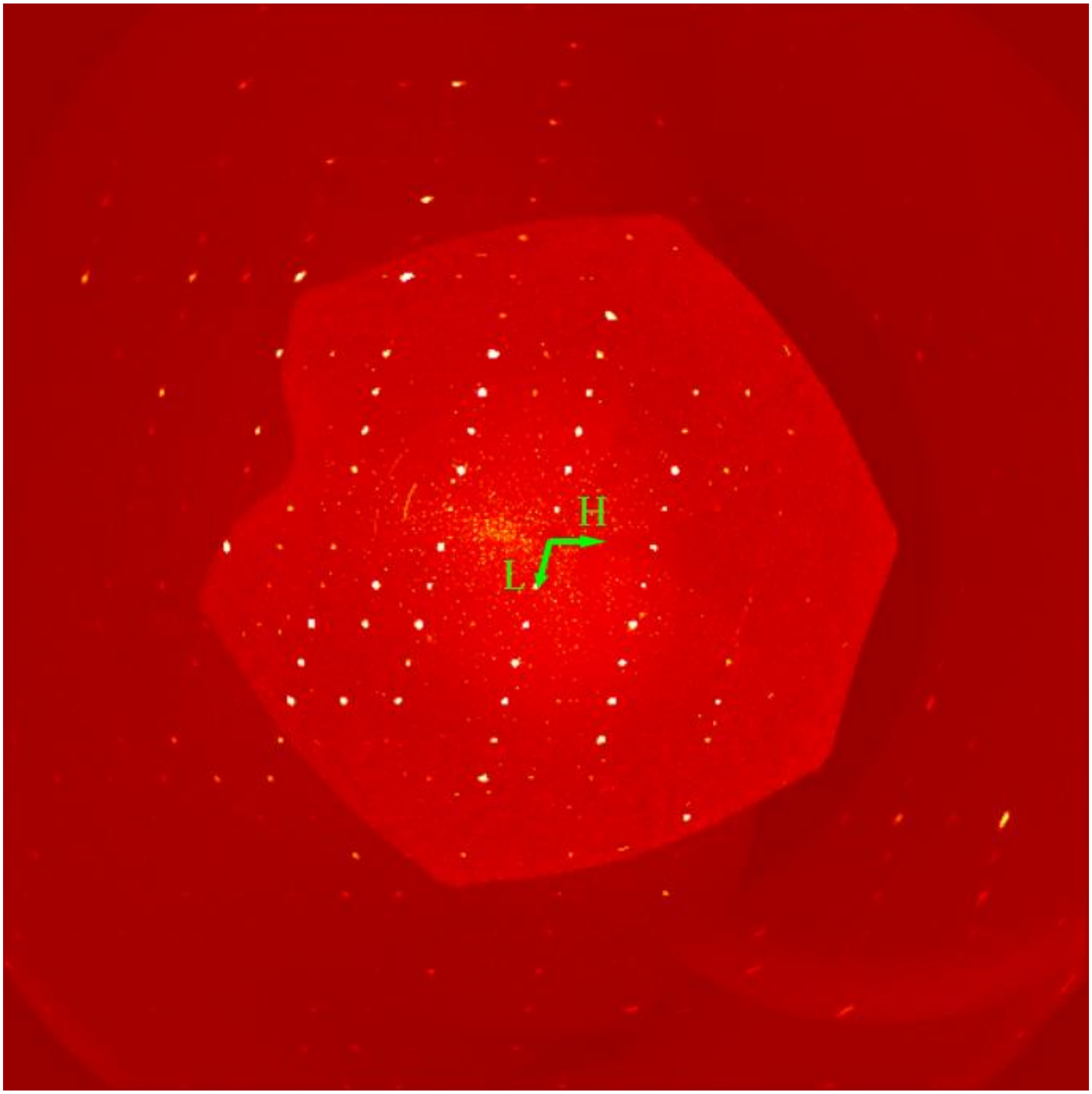

hk0 Zone

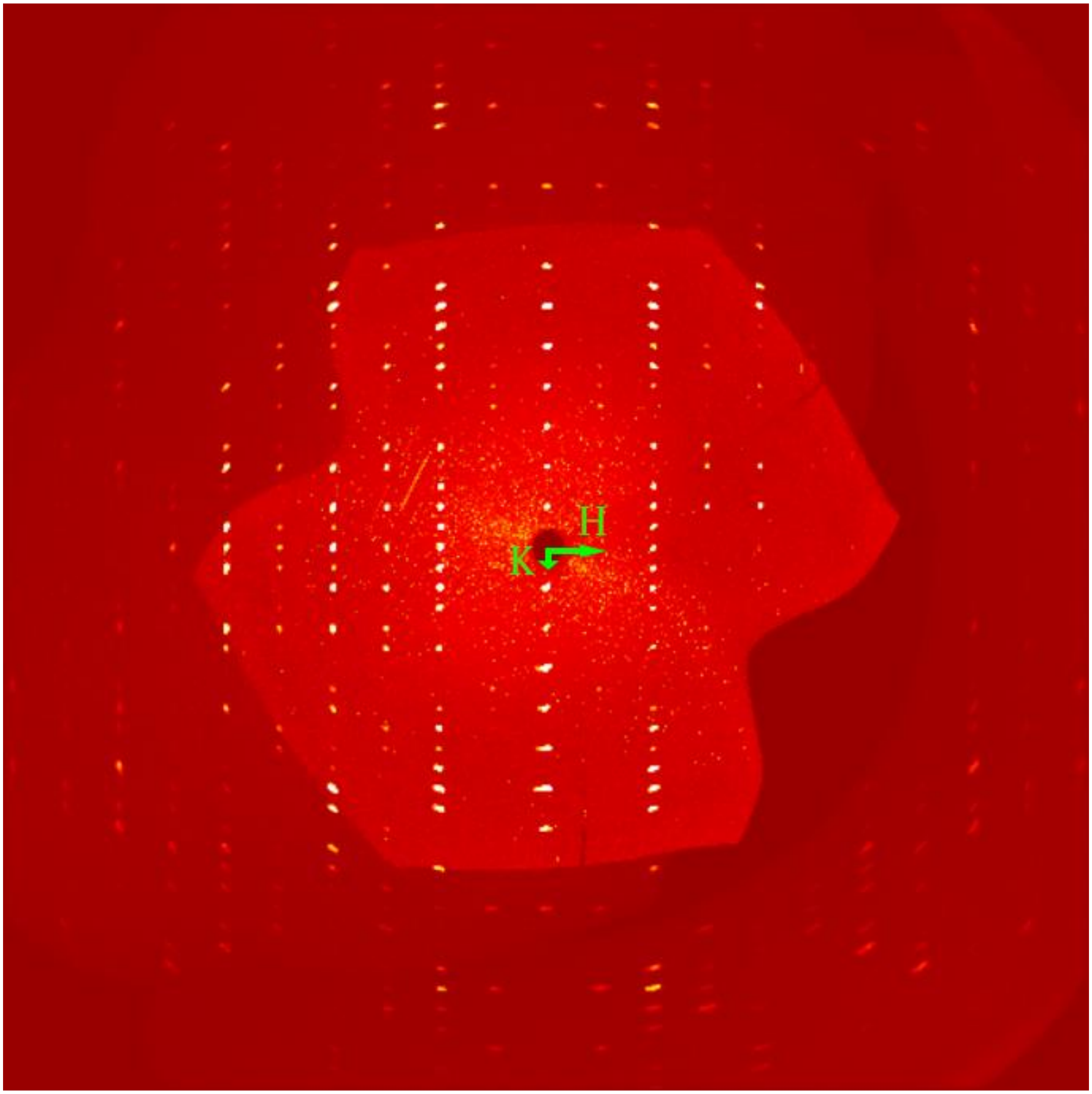

hk1 Zone

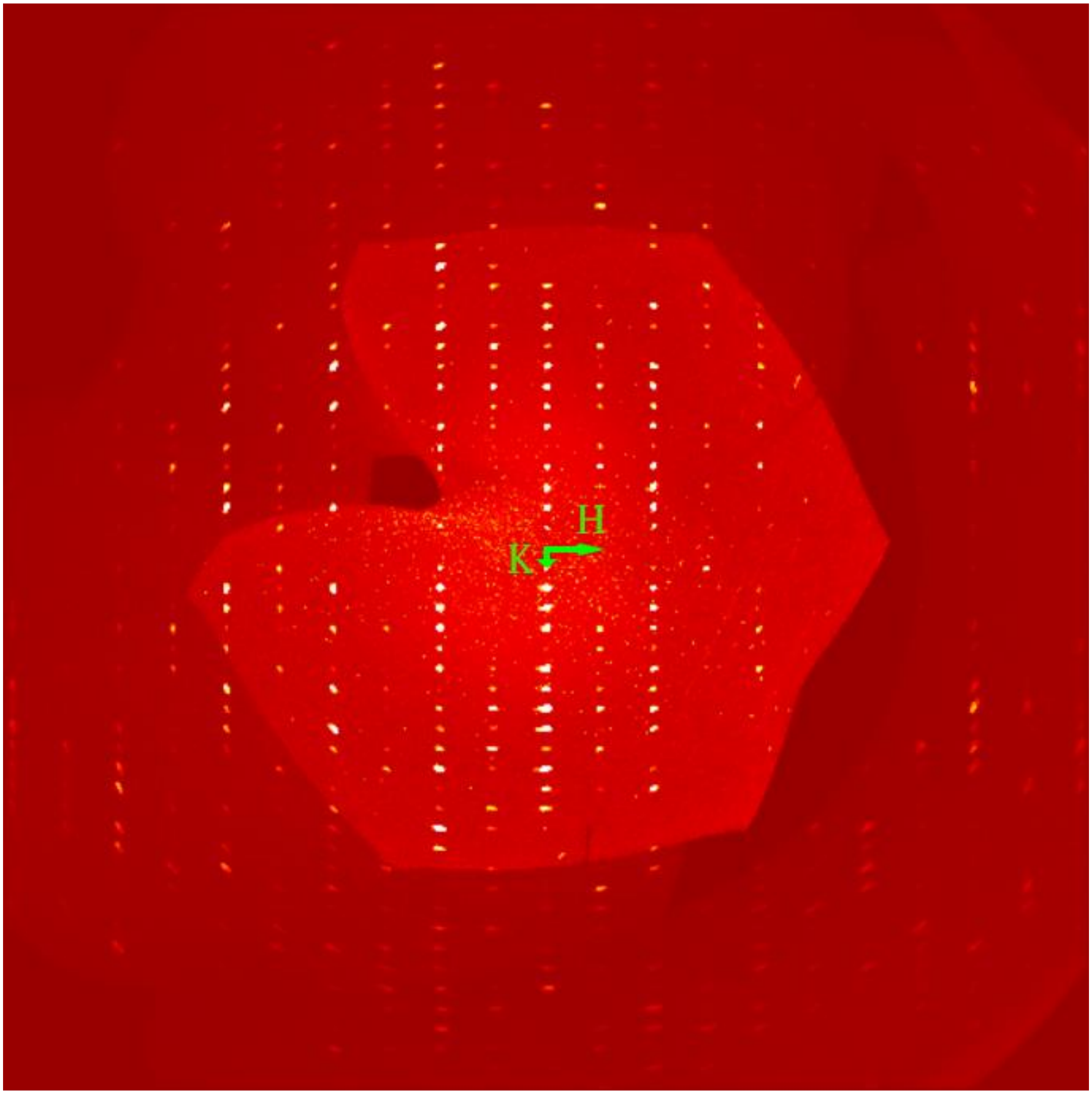

Supplement: Supplementary file 2 [file e-82-00107-sup3.zip › Data Analysis Exercise.pdf]
