# Supplementary material for: Practical aspects of teaching a graduate-level small-mol­ecule chemical crystallography course
Source: Acta Crystallogr E Crystallogr Commun. 2026 Jan 1;82(Pt 1):107–20. doi: 10.1107/S2056989025010527 (PMC12810306; doi:10.1107/S2056989025010527)
Supplement: Supplementary file 2 [file e-82-00107-sup3.zip › Direct Methods Exercise.pdf]

Reflection	$\lvert E \rvert$
$(3,3,1)$	3.74
$(\overline{25},1,4)$	3.49
$(\bar{9},6,7)$	3.25
$(12,0,0)$	3.20
$(\bar{6},9,8)$	3.05
$(\overline{13},1,4)$	2.92
$(18,4,2)$	2.92
$(6,4,2)$	2.86
$(6,0,0)$	2.80
$(9,\bar{3},\bar{1})$	2.44
$(9,7,3)$	2.07
