# Supplementary material for: Practical aspects of teaching a graduate-level small-mol­ecule chemical crystallography course
Source: Acta Crystallogr E Crystallogr Commun. 2026 Jan 1;82(Pt 1):107–20. doi: 10.1107/S2056989025010527 (PMC12810306; doi:10.1107/S2056989025010527)
Supplement: Supplementary file 2 [file e-82-00107-sup3.zip › Powder Exercises.pdf]

**2θ, °**
38.43
44.67
65.02
78.13
82.33
98.93
111.83
116.36

A powder sample of ZrO_2 examined by Cu Kα radiation (λ = 1.540 Å) gives a diffraction pattern in which the peak at 2θ = 28.2° has a full width at half maximum (fwhm) of 0.36°. In the diffraction pattern of a highly crystalline ZrO_2, the same peak has a fwhm of 0.16°. What is the average grain size of the powder sample?
