# Supplementary material for: Practical aspects of teaching a graduate-level small-mol­ecule chemical crystallography course
Source: Acta Crystallogr E Crystallogr Commun. 2026 Jan 1;82(Pt 1):107–20. doi: 10.1107/S2056989025010527 (PMC12810306; doi:10.1107/S2056989025010527)
Supplement: Supplementary file 2 [file e-82-00107-sup3.zip › Structure Factor Exercises 1.pdf]

Consider a crystal of aluminum diboride, AlB_2, with space group $P6/mmm$, which contains an Al atom at the origin and a boron atom at $\left(\frac{1}{3}, \frac{2}{3}, \frac{1}{2}\right)$. What are the intensity and the phase of the reflection $(21\bar{4})$? Assume that the scattering factors for the reflection are: $f_{Al} = 11.2$, $f_B = 4.1$.

$P6/mmm$ D^1_{6h} $6/mmm$ Hexagonal

No. 191 $P\ 6/m\ 2/m\ 2/m$ Patterson symmetry $P6/mmm$

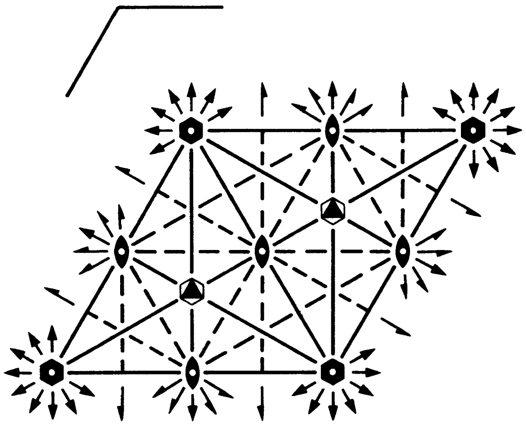

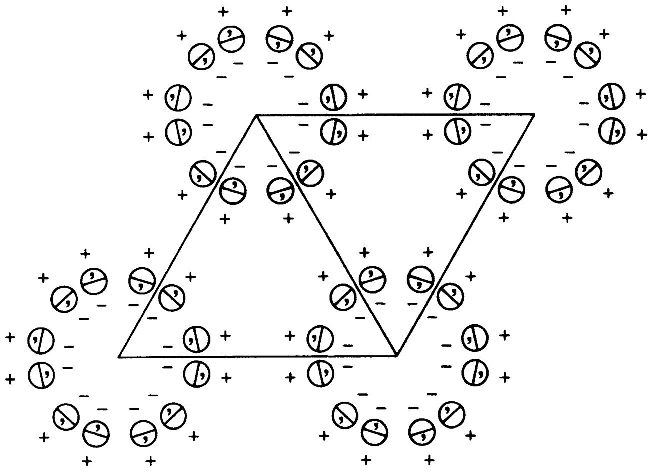

Origin at centre ($6/mmm$)

Asymmetric unit $0 \le x \le \frac{2}{3}$; $0 \le y \le \frac{1}{3}$; $0 \le z \le \frac{1}{2}$; $x \le (1+y)/2$; $y \le x/2$

Vertices $0,0,0$ $\frac{1}{2},0,0$ $\frac{2}{3},\frac{1}{3},0$

$0,0,\frac{1}{2}$ $\frac{1}{2},0,\frac{1}{2}$ $\frac{2}{3},\frac{1}{3},\frac{1}{2}$

Symmetry operations

(1) 1	(2) 3^+ $0,0,z$	(3) 3^- $0,0,z$
(4) 2 $0,0,z$	(5) 6^- $0,0,z$	(6) 6^+ $0,0,z$
(7) 2 $x,x,0$	(8) 2 $x,0,0$	(9) 2 $0,y,0$
(10) 2 $x,\bar{x},0$	(11) 2 $x,2x,0$	(12) 2 $2x,x,0$
(13) $\bar{1}$ $0,0,0$	(14) $\bar{3}^+$ $0,0,z$; $0,0,0$	(15) $\bar{3}^-$ $0,0,z$; $0,0,0$
(16) m $x,y,0$	(17) $\bar{6}^-$ $0,0,z$; $0,0,0$	(18) $\bar{6}^+$ $0,0,z$; $0,0,0$
(19) m $x,\bar{x},z$	(20) m $x,2x,z$	(21) m $2x,x,z$
(22) m x,x,z	(23) m $x,0,z$	(24) m $0,y,z$

Copyright © 2016 International Union of Crystallography

Generators selected (1); $t(1,0,0)$; $t(0,1,0)$; $t(0,0,1)$; (2); (4); (7); (13)

Positions

Multiplicity,	Wyckoff letter,	Site symmetry	Coordinates						Reflection conditions
									General:
24	r	1	(1) x,y,z	(2) $\bar{y},x-y,z$	(3) $\bar{x}+y,\bar{x},z$				no conditions
			(4) $\bar{x},\bar{y},z$	(5) $y,\bar{x}+y,z$	(6) $x-y,x,z$				
			(7) $y,x,\bar{z}$	(8) $x-y,\bar{y},\bar{z}$	(9) $\bar{x},\bar{x}+y,\bar{z}$				
			(10) $\bar{y},\bar{x},\bar{z}$	(11) $\bar{x}+y,y,\bar{z}$	(12) $x,x-y,\bar{z}$				
			(13) $\bar{x},\bar{y},\bar{z}$	(14) $y,\bar{x}+y,\bar{z}$	(15) $x-y,x,\bar{z}$				
			(16) $x,y,\bar{z}$	(17) $\bar{y},x-y,\bar{z}$	(18) $\bar{x}+y,\bar{x},\bar{z}$				
			(19) $\bar{y},\bar{x},z$	(20) $\bar{x}+y,y,z$	(21) $x,x-y,z$				
			(22) y,x,z	(23) $x-y,\bar{y},z$	(24) $\bar{x},\bar{x}+y,z$				
									Special: no extra conditions
12	q	$m\,.\,.$	$x,y,\frac{1}{2}$	$\bar{y},x-y,\frac{1}{2}$	$\bar{x}+y,\bar{x},\frac{1}{2}$	$\bar{x},\bar{y},\frac{1}{2}$	$y,\bar{x}+y,\frac{1}{2}$	$x-y,x,\frac{1}{2}$	
			$y,x,\frac{1}{2}$	$x-y,\bar{y},\frac{1}{2}$	$\bar{x},\bar{x}+y,\frac{1}{2}$	$\bar{y},\bar{x},\frac{1}{2}$	$\bar{x}+y,y,\frac{1}{2}$	$x,x-y,\frac{1}{2}$	
12	p	$m\,.\,.$	$x,y,0$	$\bar{y},x-y,0$	$\bar{x}+y,\bar{x},0$	$\bar{x},\bar{y},0$	$y,\bar{x}+y,0$	$x-y,x,0$	
			$y,x,0$	$x-y,\bar{y},0$	$\bar{x},\bar{x}+y,0$	$\bar{y},\bar{x},0$	$\bar{x}+y,y,0$	$x,x-y,0$	
12	o	$.\,m\,.$	$x,2x,z$	$2\bar{x},\bar{x},z$	$x,\bar{x},z$	$\bar{x},2\bar{x},z$	$2x,x,z$	$\bar{x},x,z$	
			$2x,x,\bar{z}$	$\bar{x},2\bar{x},\bar{z}$	$\bar{x},x,\bar{z}$	$2\bar{x},\bar{x},\bar{z}$	$x,2x,\bar{z}$	$x,\bar{x},\bar{z}$	
12	n	$.\,.\,m$	$x,0,z$	$0,x,z$	$\bar{x},\bar{x},z$	$\bar{x},0,z$	$0,\bar{x},z$	x,x,z	
			$0,x,\bar{z}$	$x,0,\bar{z}$	$\bar{x},\bar{x},\bar{z}$	$0,\bar{x},\bar{z}$	$\bar{x},0,\bar{z}$	$x,x,\bar{z}$	
6	m	$m\,m\,2$	$x,2x,\frac{1}{2}$	$2\bar{x},\bar{x},\frac{1}{2}$	$x,\bar{x},\frac{1}{2}$	$\bar{x},2\bar{x},\frac{1}{2}$	$2x,x,\frac{1}{2}$	$\bar{x},x,\frac{1}{2}$	
6	l	$m\,m\,2$	$x,2x,0$	$2\bar{x},\bar{x},0$	$x,\bar{x},0$	$\bar{x},2\bar{x},0$	$2x,x,0$	$\bar{x},x,0$	
6	k	$m\,2\,m$	$x,0,\frac{1}{2}$	$0,x,\frac{1}{2}$	$\bar{x},\bar{x},\frac{1}{2}$	$\bar{x},0,\frac{1}{2}$	$0,\bar{x},\frac{1}{2}$	$x,x,\frac{1}{2}$	
6	j	$m\,2\,m$	$x,0,0$	$0,x,0$	$\bar{x},\bar{x},0$	$\bar{x},0,0$	$0,\bar{x},0$	$x,x,0$	
6	i	$2\,m\,m$	$\frac{1}{2},0,z$	$0,\frac{1}{2},z$	$\frac{1}{2},\frac{1}{2},z$	$0,\frac{1}{2},\bar{z}$	$\frac{1}{2},0,\bar{z}$	$\frac{1}{2},\frac{1}{2},\bar{z}$	
4	h	$3\,m\,.$	$\frac{1}{3},\frac{2}{3},z$	$\frac{2}{3},\frac{1}{3},z$	$\frac{2}{3},\frac{1}{3},\bar{z}$	$\frac{1}{3},\frac{2}{3},\bar{z}$			
3	g	$m\,m\,m$	$\frac{1}{2},0,\frac{1}{2}$	$0,\frac{1}{2},\frac{1}{2}$	$\frac{1}{2},\frac{1}{2},\frac{1}{2}$				
3	f	$m\,m\,m$	$\frac{1}{2},0,0$	$0,\frac{1}{2},0$	$\frac{1}{2},\frac{1}{2},0$				
2	e	$6\,m\,m$	$0,0,z$	$0,0,\bar{z}$					
2	d	$\bar{6}\,m\,2$	$\frac{1}{3},\frac{2}{3},\frac{1}{2}$	$\frac{2}{3},\frac{1}{3},\frac{1}{2}$					
2	c	$\bar{6}\,m\,2$	$\frac{1}{3},\frac{2}{3},0$	$\frac{2}{3},\frac{1}{3},0$					
1	b	$6/m\,m\,m$	$0,0,\frac{1}{2}$						
1	a	$6/m\,m\,m$	$0,0,0$						

Symmetry of special projections

Along [001] $p6mm$
$\mathbf{a}' = \mathbf{a}$ $\mathbf{b}' = \mathbf{b}$
Origin at $0,0,z$

Along [100] $p2mm$
$\mathbf{a}' = \frac{1}{2}(\mathbf{a}+2\mathbf{b})$ $\mathbf{b}' = \mathbf{c}$
Origin at $x,0,0$

Along [210] $p2mm$
$\mathbf{a}' = \frac{1}{2}\mathbf{b}$ $\mathbf{b}' = \mathbf{c}$
Origin at $x,\frac{1}{2}x,0$

- Derive the relationship between atomic structure factors F_j for two atoms that are related by an inversion center.

- Derive the relationship between overall structure factors F and intensities F^2 for reflections at reciprocal lattice points (hkl) and $(\bar{h}\bar{k}\bar{l})$.
