# Supplementary material for: Practical aspects of teaching a graduate-level small-mol­ecule chemical crystallography course
Source: Acta Crystallogr E Crystallogr Commun. 2026 Jan 1;82(Pt 1):107–20. doi: 10.1107/S2056989025010527 (PMC12810306; doi:10.1107/S2056989025010527)
Supplement: Supplementary file 2 [file e-82-00107-sup3.zip › Structure Factor Exercises 2.pdf]

- Derive the relationship between atomic structure factors F_j for two atoms that are related by an inversion center.

- Derive the relationship between overall structure factors F, phases Φ, and intensities F^2 for reflections at reciprocal lattice points (hkl) and $(\bar{h}\bar{k}\bar{l})$.
