# Supplementary material for: Practical aspects of teaching a graduate-level small-mol­ecule chemical crystallography course
Source: Acta Crystallogr E Crystallogr Commun. 2026 Jan 1;82(Pt 1):107–20. doi: 10.1107/S2056989025010527 (PMC12810306; doi:10.1107/S2056989025010527)
Supplement: Supplementary file 2 [file e-82-00107-sup3.zip › Structure Factor Exercises 3.pdf]

- For a *C*-centered lattice, derive phases for an atom, j, at a general position (x_j, y_j, z_j), and the corresponding atom related by the *C*-centering translations. Use the difference between these phases to determine the conditions for which $F^2_{(hkl)} = 0$.

- Use a similar approach to determine the reflection conditions for two atoms related by a 2_1 screw axis along *c*.
