# Supplementary material for: Practical aspects of teaching a graduate-level small-mol­ecule chemical crystallography course
Source: Acta Crystallogr E Crystallogr Commun. 2026 Jan 1;82(Pt 1):107–20. doi: 10.1107/S2056989025010527 (PMC12810306; doi:10.1107/S2056989025010527)
Supplement: Supplementary file 2 [file e-82-00107-sup3.zip › Syllabus-613-2025.pdf]

Chem 613: Chemical Crystallography, Spring 2025

Instructors: John F. Berry; 6357 Chemistry; 262-7534; berry@chem.wisc.edu
Ilia Guzei; 2124 Chemistry; 263-4694; iguzei@chem.wisc.edu

Classroom: Chemistry Room B355
Lectures: MWF 8:50-9:40 AM
Lab Sessions: Most Fridays 8:00-10:00 AM
Credits: 3 credits
Office hours: By appointment (JFB); by appointment (IG)

Objectives: This course will introduce students to the fundamental and practical aspects of crystallography in sufficient detail to solve the structures of crystalline materials from X-ray diffraction data, and critically evaluate crystal structures reported in the literature.

Organization: The course consists of lectures and lab practical sessions, see the course schedule for more details.

Course materials will be available through the Canvas page, accessible via my.wisc.edu

Topics:

Symmetry and the Crystalline State: Transitional symmetry and the unit cell; notation for lattice planes and directions; symmetry operations; point groups and space groups.

The Theory and Experimental Aspects of X-Ray Diffraction: The geometrical conditions for diffraction; the reciprocal lattice and Ewald sphere; the form and structure factors; the selection and mounting of crystals; diffraction experiments and data analysis.

Fourier Analysis: Fourier transforms; the relationship between diffraction data and the electron density distribution within a crystal; the phase problem; Fourier maps.

Structure Solution and Refinement: Methods of structure solution, including Patterson maps, direct methods, and the charge-flipping algorithm; structure models and their refinement; validation and critique of structure refinements.

Special Topics: Depending on the time available and interest of class, additional topics could include neutron diffraction, electron diffraction, synchrotron radiation, or protein crystallography.

Grading: Grades for this course will be based on the completion of the ten problem sets to be assigned, lab practical assignments, midterm exam, and oral and written components of the final exam.

Ten problem sets	15 points each	150 points
15 practical assignments (you can do more for extra credit!)	10 points each	150 points
Midterm exam		100 points
Oral final presentation		50 points
Written final presentation		50 points
Total		500 points

Final grades are on an absolute scale. If you earn 450 points, you are guaranteed an A. Likewise for the other point totals. You are competing against this scale, and not other students. It is therefore to your benefit to help each other.

A	450 – 500 points	(90 %)
AB	439 – 449 points	(87.7 %)
B	400 – 438 points	(80 %)
BC	389 – 399 points	(77.7 %)
C	350 – 388 points	(70 %)
D	300 – 349 points	(60 %)
F	< 300 points	(< 60 %)

Suggested textbooks:

G. S. Girolami, *X-Ray Crystallography* (University Science Books)
W. Massa, *Crystal Structure Determination* (Springer)

On-line resources useful for Chem 613 include the course page (https://xray.chem.wisc.edu/education), instructional pamphlets of the International Union of Crystallography (http://www.iucr.org/education/pamphlets), and the on-line crystallography course of G. Chapuis at EPFL (http://escher.epfl.ch/eCrystallography/).

Chem 613 Problem Sets

Problem Set 1: Crystallographic Point Groups

Problem Set 2: Plane groups

Problem Set 3: Space groups

Problem Set 4: More Space Symmetry

Problem Set 5: Crystal Data and Crystal Directions

Problem Set 6: Powder Diffraction

Problem Set 7: Structure Factors

Problem Set 8: Space Group Determination

Problem Set 9: Structure Factor and Patterson Analysis

Problem Set 10: Direct Methods

Schedule of Topics, Reading Assignments, and Assignment Due Dates
CHEM 613 – X-Ray Crystallography – Spring 2025

Week	Day	Date	Topic	Reading Assignment	Assignments
1	W	22.1.2025	Overview of crystallography	Campana	
	F	24.1.2025	Point group symmetry	Girolami Chapters 1 and 2	
2	M	27.1.2025	Translational symmetry	Girolami Chapters 4 and 5	
	W	29.1.2025	Lattices	Giroalmi Chapter 6	
	F	31.1.2025	Bravais lattices	Girolami Chapter 7	
3	M	3.2.2025	Plane groups	Girolami, Chapter 8	Problem Set 1
	W	5.2.2025	Space groups	Girolami, Chapters 10, 11, 12	
	F	7.2.2025	Lab – Structure Solution		
4	M	10.2.2025	Vector algebra and matrices	Girolami, Appendix A, B	Problem Set 2
	W	12.2.2025	Diffraction of X-rays, Laue equations	Girolami, Chapters 13, 16, 17, 18	
	F	14.2.2025	Lab – Structure Solution		
5	M	17.2.2025	Reciprocal lattice	Girolami, Chapters 19, 20	Problem Set 3
	W	19.2.2025	Bragg’s law, scattering planes, *d* spacings	Girolami, Chapter 21	
	F	21.2.2025	Lab – Structure Solution		
6	M	24.2.2025	Ewald construction	Girolami, Appendix C	Problem Set 4
	W	26.2.2025	Structure factors	Girolami, Chapter 23	
	F	2.28.2025	Lab – Structure Solution		
7	M	3.3.2025	Phases, Friedel’s law, Laue classes	Girolami, Chapter 24	Midterm Exam
	W	5.3.2025	Systematic absences	Girolami, Chapter 26, 27	
	F	7.3.2025	Lab – Structure Solution		
8	M	10.3.2025	Fourier transform	Girolami, Chapter 28	Problem Set 5
	W	12.3.2025	Structure solution, the Patterson map	Girolami, Chapter 31	
	F	14.3.2025	Lab – Structure solution		
9	M	17.3.2025	Direct methods	Girolami, Chapter 34, 35	Problem Set 6
	W	19.3.2025	Charge flipping	Girolami, Chapter 30	
	F	21.3.2025	Lab – Structure solution		
10	****24.3.2025-28.3.2025 Spring Break****				

11	M	31.3.2025	Modeling the electron density	Girolami, Chapter 36	Problem Set 7
	W	2.4.2025	Refinement	Girolami, Chapter 37	
	F	4.4.2025	Lab – Structure solution		
12	M	7.4.2025	Twinning	Girolami, Chapter 38	Problem Set 8
	W	9.4.2025	Powder diffraction	Girolami, Chapter 41	
	F	11.4.2025	Lab – Structure solution		
13	M	14.4.2025	Neutron diffraction	Girolami, Chapter 42	Problem Set 9
	W	16.4.2025	Synchrotron radiation		
	F	18.4.2025	Lab – Structure solution		
14	M	21.4.2025	Protein crystallography	Girolami, Chapter 33	Problem Set 10
	W	23.4.2025	Student presentations		
	F	25.4.2025	Student presentations		
15	M	28.4.2025	Student presentations		
	W	30.4.2025	Student presentations		
	F	2.5.2025	Student presentations		
