# Supplementary material for: Practical aspects of teaching a graduate-level small-mol­ecule chemical crystallography course
Source: Acta Crystallogr E Crystallogr Commun. 2026 Jan 1;82(Pt 1):107–20. doi: 10.1107/S2056989025010527 (PMC12810306; doi:10.1107/S2056989025010527)
Supplement: Supplementary file 2 [file e-82-00107-sup3.zip › Symmetry Exercises 1.pdf]

Symmetry Exercises 1: Write down all of the symmetry elements that you see.

https://skfb.ly/o7CtY

https://skfb.ly/o7C9n

https://skfb.ly/o7C97

https://skfb.ly/o7C9t

https://skfb.ly/o7C9w

Basic Sketchfab controls:

Right-click and drag: translates the model

Left-click and drag: rotates the model

Scroll-wheel: zoom in/zoom-out
