# Supplementary material for: Practical aspects of teaching a graduate-level small-mol­ecule chemical crystallography course
Source: Acta Crystallogr E Crystallogr Commun. 2026 Jan 1;82(Pt 1):107–20. doi: 10.1107/S2056989025010527 (PMC12810306; doi:10.1107/S2056989025010527)
Supplement: Supplementary file 2 [file e-82-00107-sup3.zip › Symmetry Exercises 2.pdf]

Symmetry Exercises 2: Which point groups are represented?

https://skfb.ly/o7C9z

https://skfb.ly/o7C9B

https://skfb.ly/o7C9D

https://skfb.ly/o7C9F

https://skfb.ly/o7C9K

Basic Sketchfab controls:

Right-click and drag: translates the model

Left-click and drag: rotates the model

Scroll-wheel: zoom in/zoom-out
