# Supplementary material for: Practical aspects of teaching a graduate-level small-mol­ecule chemical crystallography course
Source: Acta Crystallogr E Crystallogr Commun. 2026 Jan 1;82(Pt 1):107–20. doi: 10.1107/S2056989025010527 (PMC12810306; doi:10.1107/S2056989025010527)
Supplement: Supplementary file 2 [file e-82-00107-sup3.zip › Symmetry Exercises 5.pdf]

CHEM 613

Symmetry Exercises 5

How do the following symmetry elements act on a point at a general position $(x, y, z)$.

• *m* plane ⊥ $\vec{a}$ at (0,0,0)

• *m* plane ⊥ $\vec{b}$ at $(0, \frac{1}{4}, 0)$

-----

• 2 ‖ $\vec{b}$

• 3 ‖ $\vec{c}$

• 4 ‖ $\vec{c}$

• 6 ‖ $\vec{c}$

-----

• *a* glide ⊥ $\vec{c}$ at $(0,0,0)$

• *n* glide ⊥ $\vec{a}$ at $\left(\frac{1}{4}, 0, 0\right)$

-----

$2_1$ along $\vec{b}$

$3_1$ along $\vec{c}$
