# Supplementary material for: Practical aspects of teaching a graduate-level small-mol­ecule chemical crystallography course
Source: Acta Crystallogr E Crystallogr Commun. 2026 Jan 1;82(Pt 1):107–20. doi: 10.1107/S2056989025010527 (PMC12810306; doi:10.1107/S2056989025010527)
Supplement: Supplementary file 2 [file e-82-00107-sup3.zip › Symmetry Exercises 7.pdf]

Symmetry Exercises #6

CHEM 613

Identify the symmetry elements present and name the space group:

1. https://skfb.ly/oq67T

2. https://skfb.ly/oqqGO

3. https://skfb.ly/oq6qw

4. https://skfb.ly/oqqGp
