# Supplementary material for: Practical aspects of teaching a graduate-level small-mol­ecule chemical crystallography course
Source: Acta Crystallogr E Crystallogr Commun. 2026 Jan 1;82(Pt 1):107–20. doi: 10.1107/S2056989025010527 (PMC12810306; doi:10.1107/S2056989025010527)
Supplement: Supplementary file 2 [file e-82-00107-sup3.zip › Symmetry Exercises 8.pdf]

CHEM 613

Symmetry Exercises #8

1. What new symmetry element is generated by the combination of a mirror plane $\perp \vec{a}$ and an *n* glide $\perp \vec{c}$? Where is it located in the unit cell?

2. Use augmented matrices to derive the six equivalent points associated with a 6_1 screw axis along $\vec{c}$.

3. Show that a body-centered monoclinic lattice can be re-indexed as a *c*-centered monoclinic lattice. Derive the transformation matrix that relates the two lattices. How many lattice points does each setting contain?
