# Supplementary material for: Practical aspects of teaching a graduate-level small-mol­ecule chemical crystallography course
Source: Acta Crystallogr E Crystallogr Commun. 2026 Jan 1;82(Pt 1):107–20. doi: 10.1107/S2056989025010527 (PMC12810306; doi:10.1107/S2056989025010527)
Supplement: Supplementary file 3 [file e-82-00107-sup4.pdf]

Obtain or Synthesize the Substance

Purify the Substance

Grow Crystals

Determine Crystal Stability

Choose the X-ray Wavelength (Mo vs Cu)

Select a Suitable Crystal under the Microscope

Mount the Crystal on the Diffractometer

Optically Align the Crystal in the X-ray Beam

Evaluate Crystal Quality by Examining the Diffraction Pattern

Obtain Unit Cell Parameters

Determine Preliminary Symmetry Information from the Diffraction Pattern

Measure Intensity Data

Integrate the Intensity Data

Perform an Absorption Correction

Assign the Lattice Type

Determine the Space Group

Solve the Structure

Assign the Identities of all Atoms in the Structure

Refine the Structure

Interpret the Structure Chemically

Write a .cif

Use CheckCIF/Platon to Validate the Structure

Publish Your Results
